# Side-by-Side Comparison of Different Thiol Bioconjugation Strategies for the Chemoselective Radiolabeling of Human Serum Albumin with Zirconium-89

**DOI:** 10.1021/acsbiomedchemau.6c00002

**Published:** 2026-04-20

**Authors:** Julia Kronberger, Barbora Neuzilova, Anja Federa, Manuel Tieber, Amando Palombo, Marie R. Brandt, Petra Heffeter, Christian R. Kowol, Milos Petrik, Thomas L. Mindt

**Affiliations:** † Institute of Inorganic Chemistry, Faculty of Chemistry, 27258 University of Vienna, Währinger Straße 42, Vienna 1090, Austria; ‡ Vienna Doctoral School in Chemistry, University of Vienna, Währinger Straße 42, Vienna 1090, Austria; § Joint Applied Medicinal Radiochemistry Facility, University of Vienna, Medical University of Vienna, Vienna 1090, Austria; ∥ Institute of Molecular and Translational Medicine, Faculty of Medicine and Dentistry, 48207 Palacký University, Olomouc 779 00, Czech Republic; ⊥ Center for Cancer Research and Comprehensive Cancer Center, 27271 Medical University of Vienna, Borschkegasse 8a, Vienna 1090, Austria; # Institute of Molecular and Translational Medicine, University Hospital, Olomouc 779 00, Czech Republic; ∇ Czech Advanced Technology and Research Institute, Palacký University, Olomouc 779 00, Czech Republic

**Keywords:** human serum albumin, positron emission tomography, thiol bioconjugation, Zirconium-89, DFO*, PET imaging

## Abstract

Different bioconjugation strategies are available for the cysteine (Cys)-specific functionalization of proteins with different payloads, including imaging probes and (pro-)­drugs. Most commonly applied linkers include maleimides (mal); however, because of the sometimes-observed instability of the formed thiosuccinimidyl linkage, its suitability for in vivo applications has been challenged. Consequently, several alternatives have been developed and compared to mal as a benchmark, yet examples of a direct comparison among new methodologies are scarce. We herein report a comparison of the use of mal, phenyloxadiazole methyl sulfone (PODS), and vinylketone (VK) as functional groups for the thiol-specific functionalization of human serum albumin (HSA) via its available free Cys^34^. Bifunctional chelating agents (BFCA) based on DFO*, identical in all regards but the functional group for bioconjugation, were prepared and conjugated to HSA and the obtained DFO*-HSA conjugates were radiolabeled with Zirconium-89 (^89^Zr) for positron emission tomography (PET). The efficiency of the conjugation of DFO*-X (X = mal, PODS, VK) to HSA differed significantly, with mal > PODS > VK. Stability studies of the ^89^Zr-labeled HSA-conjugates indicated good stability for [^89^Zr]­Zr-DFO*malHSA **11** and [^89^Zr]­Zr-DFO*-POD-HSA **12** in blood serum but only the latter was found stable in cell culture medium. [^89^Zr]­Zr-DFO*VK-HSA **13** was excluded from biological experiments due to its surprisingly low stability in all media tested. [^89^Zr]­Zr-DFO*-POD-HSA **12** was further investigated in CT26-tumor-bearing mice by PET/CT imaging and biodistribution studies. Specific uptake of radioactivity in tumors was high (up to 17% ID/g) and the tumors could be clearly visualized by PET at all time points with excellent tumor-to-background signal (tumor-to-blood ratio 3.2 ± 1.0 after 48 h p.i.). Unexpectedly, the uptake of radioactivity in bones was observed for [^89^Zr]­Zr-DFO*-POD-HSA **12**. Overall, the in vivo performance of [^89^Zr]­Zr-DFO*-labeled HSA obtained by mal chemistry is the most promising candidate as a companion diagnostic PET imaging probe for the stratification of patients for therapies based on HSA-binding (pro-)­drugs.

## Graphical abstract

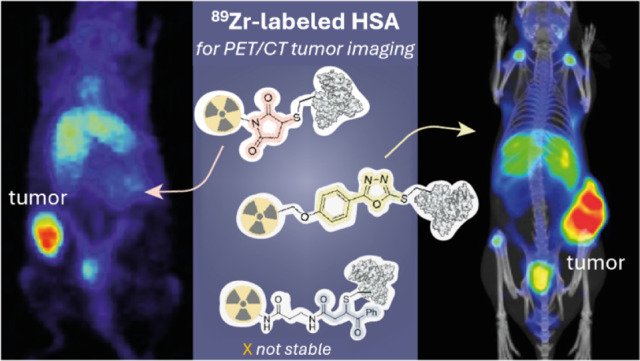

## Introduction

Human serum albumin (HSA) is the most abundant plasma protein, with a concentration of 35–50 mg/mL in human blood plasma and an exceptionally long plasma half-life of about 19 days.^1^ HSA has been shown to accumulate in solid tumors^2,3^ based on the enhanced permeability and retention (EPR) effect as well as increased consumption of the protein by tumor cells as nutrition source.^4–7^ In more detail, in healthy cells, the neonatal Fc receptor (FcRn) allows for constant recycling of HSA from lysosomes back to the bloodstream by binding and thereby protecting it from intracellular degradation.^8–10^ Tumor cells often lack FcRn, which leads to degradation of albumin and consumption of its amino acids.^11,12^ The long plasma half-life of albumin is leveraged as a drug-delivery system to extend the plasma circulation time, stability, and tumor uptake of payloads by implementing albumin-binders into their structure. The latter can bind either covalently (e.g., maleimide (mal)) or noncovalently to the protein (e.g., fatty acids).^13,14^ For example, Aldoxorubicin is a doxorubicin-releasing prodrug, which binds selectively to albumin via a mal moiety upon iv administration.^15^ It has been tested for the treatment of soft tissue sarcoma in a clinical phase 3 trial (NCT2049905). Clinical examples exploiting noncovalent HSA binding are the long-acting GLP-1 analogs semaglutide (e.g., Ozempic) and liraglutide (e.g., Saxenda) which are FDA-approved for the treatment of type 2 diabetes and chronic weight management.^16^

HSA offers an ideal platform to investigate chemoselective cysteine (Cys) modifications of proteins. In HSA, only one (Cys^34^) out of its 35 Cys is available for modifications, while the others form disulfide bridges important for the secondary and tertiary structure of the protein.^17^ Accordingly, modification of this residue results in homogeneous and well-defined bioconjugates. Cys^34^ is accessible in reduced form (thiol) in about 70–80% of the HSA population in healthy young adults.^18^ The others are oxidized to sulfenic or sulfinic acids or form disulfides with other thiols, such as glutathione.^19,20^ The most commonly used Cys-based bioconjugation method is Michael addition of mal. The 1,4-addition occurs rapidly (*k*_s_ ∼ 10^6^ M^–1^ min^–1^ for small-molecule systems,^21^ *k*_s_ ∼ 10^2^–10^4^ M^–1^ min^–1^ for reactions with peptides or proteins^22,23^ ) under physiological conditions,^21^ but stability issues of the resulting thiosuccinimidyl linkage are known. Even though mal-based antibody-drug conjugates perform well in vivo, retro-Michael additions can occur.^24,25^ Also, the thiosuccinimidly bond is known to undergo hydrolysis, which results in an altered, yet still intact and stable linkage.^26^

As a result, several alternatives to mal for bioconjugations via Cys-residues have been developed.^27,28^ Among other examples, Bernardim et al. reported vinyl ketones (VK) to form selective and stable bonds with Cys by Michael addition and the late Barbas and coworkers published on phenyloxadiazolyl methyl sulfone (PODS) as a class of compounds, reacting selectively with thiols of Cys by nucleophilic aromatic substitution (S_N_Ar) and resulting in a phenyloxadiazole (POD) linkage after release of the methyl sulfone group.^29,30^ While new, thiol-selective reactive groups for bioconjugations are usually compared to mal as a benchmark, reports of a direct side-by-side assessment among such new reagents are still scarce.^31–34^

In a previous study, we investigated [^89^Zr]­Zr-DFO*malHSA **11** as a potential companion diagnostic nuclear imaging probe for HSA-binding drugs and prodrugs in a personalized medicine approach.^35^ The radiotracer was prepared by conjugating HSA via mal-chemistry to the DFO*, an octadentate chelator which provides, in contrast its predecessor the hexadentate chelator DFO, complexes with ^89^Zr of high stability in vivo.^36,37^ DFO* belongs to a class of novel, octadentate ligand systems increasingly employed successfully in ^89^Zr-PET applications.^38–40^ [^89^Zr]­Zr-DFO*malHSA **11** was found stable in vivo, and cancer lesions could be clearly visualized by positron emission tomography (PET) in different mouse models for colorectal cancer. During this work, we observed low stability of [^89^Zr]­Zr-DFO*malHSA **11** in vitro, especially in commonly used cell culture media, which might provide an explanation why in vitro experiments with mal-(bio)­conjugates are often not described in the literature. Although in vitro testing of compounds may have limited translational value in some cases, it remains a vital step in the development of new drugs. Skipping this stage of drug development would significantly increase reliance on animal experiments, contravening the principles of the 3Rs—replacement, reduction, and refinement—which guide the ethical and responsible use of animals in research.^41^

We herein describe the synthesis of novel thiol-reactive VK and PODS derivatives of the ^89^Zr-chelator DFO*.^36,40^ This study includes a side-by-side assessment of their reactivity toward Cys^34^ of HSA, efficiency of ^89^Zr-radiolabeling and stability of ^89^Zr-labeled HSA conjugates. The new derivatives and their HSA-conjugates were compared to the previously reported mal-derivative [^89^Zr]­Zr-DFO*malHSA **11**. The most promising new candidate, [^89^Zr]­Zr-DFO*-POD-HSA **12**, was applied in Balb/c mice bearing CT26-tumors for PET/CT imaging (up to 6 days p.i.) and ex vivo biodistribution studies.

## Results and Discussion

### Chemical Synthesis

The ^89^Zr-chelator, [DFO*NH_2_]­TFA **1**, was synthesized following a previously published optimized procedure (Supporting Information (SI), Figures S1–S6).^42^ HOOC-mal **2** was purchased from a commercial supplier. The synthesis of HOOC-PODS **3** was adapted from literature^43^ (Scheme S1a). Briefly, 3-iodo-1-propanol was reacted with ethylparabene to obtain OH-Ph-COOEt S1, which was then reacted with N_2_H_2_·H_2_O to yield OH-Ph-hydrazide S2. Next, the reaction to form the 1,3,4-oxadiazole moiety was achieved using CS_2_ yielding OH-POD-SH S3 and the resulting thiol was methylated with iodomethane to obtain OH-POD-SMe S4. For optimal yield, selective oxidation of the aliphatic alcohol and methyl sulfide moieties was carried out. First a Jones oxidation was performed to synthesize carboxylic acid COOH-POD-SMe S5, followed by sulfur oxidation using H_2_O_2_ with catalytic ammonium molybdate, affording COOH-PODS **3** in 25% yield over six steps. HOOC-VK **4** was synthesized, starting with a peptide coupling reaction of (*E*)-4-oxo-4-phenylbut-2-enoic acid and *tert*-butyl 3-aminopropanoate·HCl, using isobutyl chloroformate as the coupling reagent (Scheme S1b). *Tert*-butyl deprotection with TFA afforded HOOC-VK **4** in 74% yield over two steps. The intermediates were then attached to DFO* **1** via amide coupling in DMF using microwave-assisted heating and the products DFO*-PODS **6** and DFO*VK **7** characterized by HPLC, NMR and HRMS (Scheme S1, Figures S7–S18). In the case of compound **6**, a prominent side product with formation of a terminal vinyl ketone (DFO*shortVK **S7**) (Scheme S2, Figures S19–S24) was isolated, likely arising from base-induced elimination of the PODS group.

The bifunctional chelating agents (BFCA) DFO*mal **5**, DFO*-PODS **6** and DFO*VK **7** were designed to be structurally identical, differing only in the respective thiol-selective functional group. This allows for a direct comparison of the influence of the bioconjugation strategy on the radiolabeling properties, stability and biological behavior of the respective HSA-adduct (Figure 1). The novel DFO*-based BFCA DFO*PODS **6** and DFO*VK **7** were obtained by-multistep syntheses in moderate overall yields (9% and 27% yield, respectively) and high purity (>98%, HPLC).

**Figure 1:**
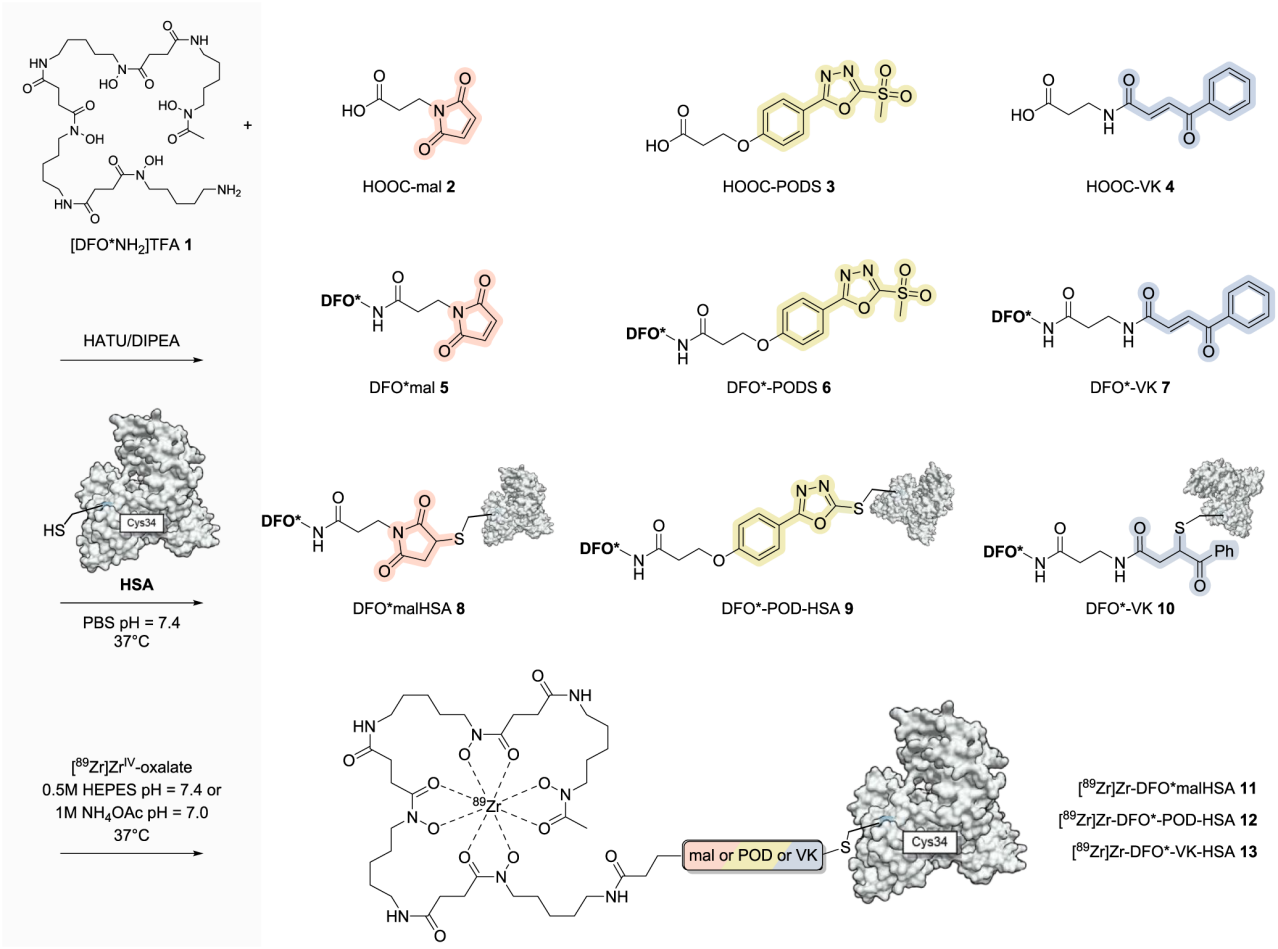
Overview of the compounds investigated. Compounds **2**, **5**, **8**, and **11** were previously published and are included in this study for comparison.^35^ The structure of HSA was rendered from the protein database (PDB) entry 1AO6^17^ using Mol*.^44^

### Reactivity of Mal, PODS, and VK toward a Cys-Containing Model Peptide

Because of the known limited solubility of DFO* and its derivatives, we had to resort to a model system for the assessment and comparison of the reactions kinetics. Thus, the more soluble carboxylic acid derivatives **2**–**4** were incubated at 37 °C in equimolar amounts (0.25 mM in 50 mM NaOAc buffer) with Ac-QQCPF-NH_2_ **14**, a short *N*-acetylated fragment of the HSA amino acid sequence containing the Cys^34^ (amino acids 32–36) (Figures S25–S26). Samples were analyzed by HPLC and RP-HPLC-MS (Figures S27–S30).

As expected, mal **2** reacted with peptide **14** very quickly and quantitative conversion to conjugation product Ac-QQC­(mal)­PF-NH_2_ **15** was achieved already after 10 min. The reactions of substrates **3** and **4** to the respective products Ac-QQC­(POD)­PF-NH_2_ **16** and Ac-QQC­(VK)­PF-NH_2_ **17** were fast during the first 10 min as well but then slowed down. For oxadiazole **3**, it took 30 min for completion of the reaction and in the case of VK **4**, the formation of minor amounts of a nonidentified, unreactive side product was observed. Related reported studies compare the reaction rates of simple, small-molecule derivatives of mal vs PODS^45^ and mal vs VK^29^ reacting with *N*-acetyl-l-cysteine methyl ester and methyl thiolate, respectively. Our data are in line with findings of these studies in terms of the faster reaction of thiols with mal than PODS. However, the reported comparable reactions rates of mal and VK could not be confirmed.

From these results, we concluded that while mal reacted faster than PODS and VK, the reaction kinetics of all three functional groups are suitable for bioconjugation reactions with Cys-containing proteins in vitro.

### Bioconjugation

Conjugation of the novel DFO*-based BFCA to HSA was investigated in NaOAc buffer (pH 5.5) or PBS (pH = 7.4). These buffers were chosen based on the integrity of the protein structure and reactivity of the thiol of Cys.^18,34,46,47^ Thus, a 4-fold molar excess of the BFCA **5**, **6**, and **7** was dissolved in DMSO and diluted with the buffers, followed by the addition of HSA in 0.9% NaCl (final concentrations BFCA = 0.2 mM, HSA = 0.05 mM). The solutions were incubated at 37 °C for 0.75–1.5 h. The bioconjugates were purified via PD-10 columns and spin filtration. For some reactions, 1 equiv of tris­(2-carboxyethyl)­phosphine (TCEP) was used to liberate more Cys^34^ and achieve a higher thiol-to-protein ratio of 0.53 (vs 0.24 without TCEP). The thiol-to-protein ratio was determined before bioconjugation reactions via 5,5-dithio-bis­(2-nitrobenzoic acid) (DTNB) assays, and size-exclusion chromatography (SEC) was performed to verify the integrity of DFO*malHSA **8**, DFO*-POD-HSA **9**, and DFO*VK-HSA **10** (Figures S31–S33).

To determine the chelator-to-protein ratio, products were analyzed further by MS and isotopic dilution assays.^48^ The ratio represents the percentage of HSA molecules that are available for cysteine-selective modification. The first, qualitative method has been previously applied successfully to DFO*malHSA **8**,^35^ but was inconclusive for conjugates **9** and **10**. On the other hand, the isotopic dilution assays using an excess of ^nat^Zr, spiked with ^89^Zr, gave good and reproducible chelator-to-protein ratios for all conjugates. The obtained values were compared to the available thiol groups of HSA as determined by the DTNB assay beforehand (Table 1 and Table S1).

**Table 1:** Representative Examples of Bioconjugation Reactions of **8**, **9**, and **10** in NaOAc Buffer or PBS at pH 5.5 or pH 7.4, Respectively, with Obtained Chelator-to-Protein Ratios Relative to Available Thiols per HSA Molecule

				chelator-to-protein ratio^b^		
buffer (pH)	chelator [equiv]	*t* [h]	available thiol per HSA^a^	8	9	10
NaOAc (5.5)	4	0.75	0.53	-	0.01	0.02
NaOAc (5.5)	4	1.5	0.53	-	0.01	0.00
PBS (7.4)	4	0.75	0.53	0.53	0.19	0.26
PBS (7.4)	4	1.5	0.24	0.23	0.05	0.67^c^
PBS (7.4)	4	3.5	0.24	0.37^c^	0.10	0.39^c^

a Determined by DTNB assays.

b Determined by isotopic dilution assays.

c Values higher than available free thiols indicate unselective reaction of the BFCA with other amino acid residues.

BFCA **5** reacted quantitatively with the free thiol of Cys^34^ of HSA in PBS at pH 7.4 to give DFO*malHSA **8** within 0.75 h. At the 3.5 h time point unselective reaction with other amino acid residues occurred as indicated by a chelator-to-protein ratio exceeding the possible value of SH/HSA. It is known that mal can react, although at a factor 1000 slower, with primary amines in the absence of available thiols.^49^ The bioconjugation reaction of DFO*-PODS **6** with HSA to yield DFO*-POD-HSA **9** in PBS (pH 7.4) showed low conversion of the available thiol of HSA even at a prolonged reaction time. A previous study with PODS-DFO for conjugation to an antibody successfully used similar conditions (PBS pH = 7.2, 4 equiv, 1.5 h).^33^ However, DFO was “protected” by its Fe-complex, and thus, these data are not suitable for a direct comparison. DFO*-VK **7** seemed to react unselectively with HSA in PBS at longer reaction times required for good conversion of its Cys^34^, as indicated by the chelator-to protein ratios of conjugate **10** higher than theoretically possible. These results are not in accordance with published data on the VK moiety, which reported Cys-selective bioconjugation reactions at 37 °C in buffer at pH 7.0–8.0.^50^ The bioconjugation reactions of HSA-adducts **9** and **10** by PODS- and VK-derivatives of DFO* **6** or **7** respectively were also performed in NaOAc buffer at pH 5.5. Bioconjugation reactions of DFO*mal **5** were not performed in NaOAc since PBS already worked well for this reaction.^35^ The buffer was chosen based on our kinetic studies. However, the BFCAs **6** and **7** did not react with HSA in this buffer.^18,46,47^

Since the chelator-to-protein ratios did not improve over time for DFO*-POD-HSA **9** in PBS, we decided to choose 0.75 h as a sufficient reaction time for the bioconjugation reactions. For DFO*VK-HSA **10**, 0.75 h was also chosen as the preferred reaction time, since unselective modification of amino acid residues other than Cys^34^ was thereby minimized. For radiopharmaceutical development, optimization of the specific molar activity A_S_ [Bq/mol] of ^89^Zr-labeled HSA is not as crucial as it is for other radiotracers due to immediate dilution after intravenous injection by naturally abundant HSA in the blood. We therefore considered the achieved ratio of DFO* conjugated to HSA as sufficient for our purpose. Despite comparable reaction rates of BFCAs **5**, **6**, and **7** in the kinetic studies with a model peptide, bioconjugation with HSA gave significantly different results, probably due to steric effects or varying reactivity of Cys^34^ at different pH.

### Radiolabeling

Radiolabeling of conjugates DFO*malHSA **8**, DFO*-POD-HSA **9**, and DFO*VK-HSA **10** with [^89^Zr]­Zr-oxalate was performed as previously described.^35^ Due to the limited stability of radioconjugates [^89^Zr]­Zr-DFO*-POD-HSA **12** and [^89^Zr]­Zr-DFO*VK-HSA **13** in 4-(2-hydroxyethyl)-1-piperazineethanesulfonic acid (HEPES) buffer (pH = 7.4), 1 M ammonium acetate (pH = 7.0) was investigated as well.^40^ In brief, the conjugates (0.5 mg) were incubated in the respective buffer at 37 °C with 5–25 MBq of [^89^Zr]­Zr^4+^ for 3–5 h to yield [^89^Zr]­Zr-DFO*mal-HSA **11**, [^89^Zr]­Zr-DFO*-POD-HSA **12**, and [^89^Zr]­Zr-DFO*VK-HSA **13**. To increase the radiochemical yield (RCY), higher amounts of precursor and radioactivity as well as longer reaction times were applied. The crude radiolabeling solution was purified and thereby reformulated in 0.9% NaCl in two steps: First, via a PD-10 column and then by preparative spin filtration. The quality control comprised three methods: instant thin-layer chromatography (iTLC), SEC, and spin filter analysis (Figure S34). With iTLC, the amount of free radionuclide ([^89^Zr]­Zr^4+^) was quantified (*r*_f_ = 1), but not the fraction of radiolabeled chelator ([^89^Zr]­Zr-DFO* and [^89^Zr]­Zr-DFO*-HSA, both *r*_f_ = 0), the latter of which could potentially be released from the protein. SEC provided qualitative information about the successful radiolabeling of the protein and detection of aggregates. A reliable determination of the radiochemical purity (RCP) ^89^Zr-labeled HSA was only possible via spin filtration analysis in addition to iTLC and SEC as previously reported (Figures S34–S37).^43^ The molecular weight cutoff of the used spin filters (30 kDa) enabled the separation of low-molecular weight species (free [^89^Zr]­Zr^4+^ and [^89^Zr]­Zr-DFO*) from the protein. Unspecific binding of [^89^Zr]­Zr^4+^ and complexed ^89^Zr to the filter membrane was verified to be <1%.

The novel radiotracers [^89^Zr]­Zr-DFO*-POD-HSA **12** and [^89^Zr]­Zr-DFO*VK-HSA **13** were obtained in moderate RCY in both buffers investigated (Table 2). Unlike in the case of [^89^Zr]­Zr-DFO*malHSA **11**, the RCY for radiolabeled conjugates **12** and **13** could not be increased by extending the incubation time. For [^89^Zr]­Zr-DFO*-POD-HSA **12**, the lower RCY compared to that of mal derivative **11** is due to its lower chelator-to-protein ratio (Table 1). This leads to lower RCY as indicated by the high amount of free [^89^Zr]­Zr^4+^ in the crude radiolabeling solution. On the other hand, the instability of the VK-linkage of [^89^Zr]­Zr-DFO*VK-HSA **13** is accountable for the low RCY, as indicated by the low levels of free [^89^Zr]­Zr^4+^ and increased amounts of [^89^Zr]­Zr-DFO* (Figure S34). NH_4_OAc buffer was used for the synthesis of the radiolabeled conjugates **12** and **13** due to their higher stability compared to that of HEPES buffer. Despite moderate RCY, all radiotracers were obtained after purification with a final RCP of >95% and sufficient A_s_ for biological experiments.

**Table 2:** Radiolabeling Results of [^89^Zr]­Zr-DFO*malHSA **11**,^35^ [^89^Zr]­Zr-DFO*-POD-HSA **12**, and [^89^Zr]­Zr-DFO*VK-HSA **13** at Different Conditions (Amount of Protein, Amount of Radioactivity, Buffer) Investigated for the Radiolabeling Reactions Including the Final RCY and Analytical Data of the Crude Radiolabeling Solutions (before Purification) Are Given (RCP and Amount of Free [^89^Zr]­Zr^4+^)

			***a*** ^b^ **[MBq]**		**RCY [%]**		**RCP (crude) [%]**		**Free [** ^**89**^ **Zr]Zr** ^**4+**^ **(crude) [%]**	
product	buffer^a^	HSA [mg]	MEAN ± SD	*n*	MEAN ± SD	*n*	MEAN ± SD	*n*	MEAN ± SD	*n*
**11**	HEPES	0.5	16.8 ± 2.0	6	75 ± 9	6	80 ± 10	6	3 ± 2	6
	HEPES	1.0	163.5	1	70	1	73	1	1	1
**12**	HEPES	0.5	17.3 ± 5.5	3	33 ± 9	3	62	1	44	1
	NH_4_OAc	0.5	15.8 ± 5.8	5	36 ± 22	5	53 ± 28	5	52 ± 28	5
	NH_4_OAc	1.0	134.6	1	27	1	24	1	69	1
**13**	HEPES	0.5	21.0 ± 1.4	2	24 ± 1	2	27	1	0	1
	NH_4_OAc	0.5	20.0	1	19	1	24	1	0	1

a HEPES = 0.5 M HEPES buffer, pH 7.4, NH_4_OAc = 1 M ammonium acetate, pH = 7.0.

b Amount of radioactivity used for the radiolabeling reaction. RCP of the purified radiotracer was always >95%.

### Stability

The stability of radiolabeled HSA conjugates **11**–**13** was assessed in different media at 37 °C. At different time points for up to 4 or 7 d (relevant time span for in vitro and in vivo experiments, respectively), samples were analyzed by spin filter analysis. A notable limitation of the method is its inability to detect reformed conjugates that could potentially occur after a Retro-Michael reaction and subsequent reaction with another protein.^51^

Results for the stability of the radiotracers over 4 h and 7 days are summarized in Figure 2. Data for [^89^Zr]­Zr-DFO*malHSA **11**, except for buffers, have been published previously and are included for comparison.^35^ The tracer remained stable (>95%) over 4 h in all tested media, except for RPMI-1640 (>70%), for 7 d in 0.9% NaCl and largely in in human blood serum (>85%). [^89^Zr]­Zr-DFO*-POD-HSA **12** remained stable over 4 h in all media (>90%) except in HEPES buffer (<40%). Over 5 d, the compound was stable in 0.9% NaCl and serum (>90%) and after 7 d, >85% stayed intact. [^89^Zr]­Zr-DFO*VK-HSA **13** was found to be unstable in any medium tested, and degradation products resulting from hydrolysis or reactions with components of cell culture medium were detected already after 1 h, even in 0.9% NaCl. Over several days, the amount of intact compound **13** stabilized at approximately 60%, in both 0.9% NaCl and serum, probably because the equilibrium of the Retro-Michael reaction is reached. In a recently published study on antibody fragments labeled with the β-emitter rhenium-188 (^188^Re), using the same VK linker, instability of the radioconjugate in 0.9% NaCl was also noted.^52^ However, the reported stability of the ^188^Re-labeled protein conjugate in human plasma at 37 °C for 2 d could not be confirmed for our ^89^Zr-labeled HSA-VK conjugate. Due to the insufficient stability of [^89^Zr]­Zr-DFO*VK-HSA **13**, this compound was not further investigated in biological experiments.

**Figure 2:**
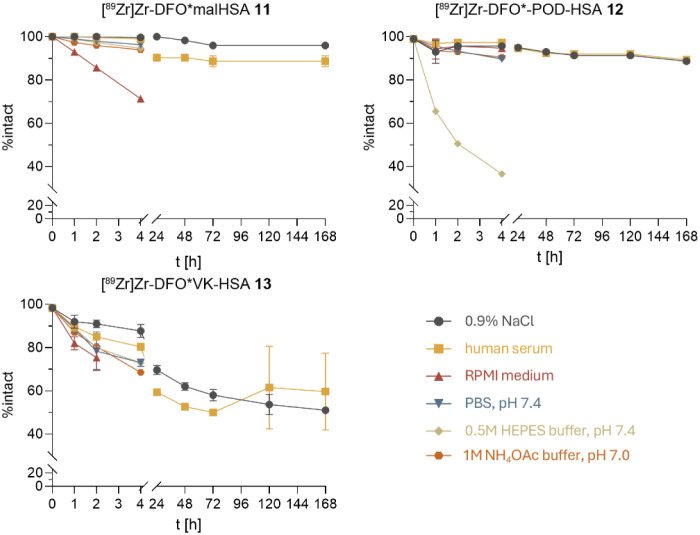
Stability of [^89^Zr]­Zr-DFO*malHSA **11**, [^89^Zr]­Zr-DFO*-POD-HSA **12**, and [^89^Zr]­Zr-DFO*VK-HSA **13** over 4 h and 7 d in different media: human blood serum, 0.9% NaCl as formulation for in vivo experiments, cell culture medium RPMI-1640 as used for cell experiments, PBS buffer as mobile phase used in SE-HPLC and the buffers HEPES and NH_4_OAc for investigation of the radiolabeling conditions. Data for **11**, except for the buffers, have been published previously and are included for comparison.^35^ Results are mean values of 3 ± SD. Some symbols and curves are overlapping.

In general, the stability of ^89^Zr-labeled DFO*-HSA conjugates in different media varied depending on the bioconjugation chemistry. While [^89^Zr]­Zr-DFO*malHSA **11** and [^89^Zr]­Zr-DFO*-POD-HSA **12** showed promising serum stability suitable for in vivo experiments, only the latter is preferred for in vitro experiments on cells because of its stability in cell culture medium.

Besides the chemical stability of the linkage between the ^89^Zr-complex and the protein, the chemical structure could also affect the physicochemical and biological properties of a radiometal-based radiotracer. For example, Vugts et al. reported the possibility of a thiourea linkage, derived from using DFO*-pPhe-NCS for conjugation to antibodies, to interfere with the complexation of ^89^Zr by DFO* by additional, nonproductive interactions of the sulfur atom with the metal.^37,53^ Donnelly and coworkers postulated the octadentate coordination of [^89^Zr]­Zr^4+^ through the carbonyl oxygens of the squaramide ester functionality as rationally incorporated in the BFCA used in their study.^54^ Similarly, Denat and coworkers reported the influence of the bioconjugation linker on the stability of ^89^Zr-labeled antibodies, particularly in the case of radiolysis.^55^ Thus, the selected functional group of a BFCA could not be an innocent bystander but may affect the stability of the ^89^Zr-complex. In the future, this observation should be considered in the design of BFCA, e.g., by including longer linkers that separate the radiometal complex from the linkage to (bio)­molecules of interest.

### Cell Culture Experiments

We have previously published results from in vitro cell culture experiments with [^89^Zr]­Zr-DFO*malHSA **11**, which revealed a very low and nonreproducible cell-binding and uptake of radioactivity in CT26 colorectal cancer cells, which did not reflect the reported cell uptake of fluorescently labeled HSA.^35^ Due to the instability of the mal linkage, short-time experiments were performed in Hanks’ balanced salt solution instead of preferred cell culture media like RPMI-1640, in which cells can survive for longer time periods. With the stable [^89^Zr]­Zr-DFO*-POD-HSA **12** in hand, we repeated the in vitro experiments in RPMI-1640 medium using again CT26 and additionally SW480 colorectal cancer cells. However, even with the new system of improved stability, no significant cellular uptake of radiolabeled HSA in CT26 and SW480 cells could be observed at different concentrations of HSA (Figure S38).^56^ These findings confirm our previous rationalization that, likely among other factors, the EPR effect and the tumor microenvironment play pivotal roles in the mechanism of the tumor uptake of HSA, which cannot be mimicked easily by our 2D cell models.

### In Vivo PET/CT Imaging

PET/CT imaging was performed in two Balb/c mice bearing sc CT26-allografts that received 10.4 and 9.0 MBq of [^89^Zr]­Zr-DFO*-POD-HSA **12** corresponding to 266 and 231 μg of protein, respectively. It should be noted that the amount of injected HSA was 3–10 times higher than in the previous study with [^89^Zr]­Zr-DFO*malHSA **11** in order to inject a similar dose of radioactivity of [^89^Zr]­Zr-DFO*-POD-HSA **12**. We selected this allograft model to compare the results to our previous study with [^89^Zr]­Zr-DFO*malHSA **11**.^35^ The animals were repeatedly imaged by PET/CT at 1, 2, 3, and 6 d p.i. of the radiotracer (Figure 3 and Figure S39). Overall, the PET/CT images were similar to those obtained with [^89^Zr]­Zr-DFO*-mal-HSA **11**. [^89^Zr]­Zr-DFO*-POD-HSA **12** showed moderately rapid pharmacokinetics with major accumulation in the excretory organs, tumor, highly perfused organs in the early (1 d p.i.), and joints in later (2–6 d p.i.) time points. After 1 day, there was no more radioactivity visible in highly perfused organs, indicating good blood clearance of [^89^Zr]­Zr-DFO*-POD-HSA **12** and promising potential for favorable tumor-to-background ratios. Since [^89^Zr]­Zr-DFO*-POD-HSA **12** accumulated highly in the tumors, it could be clearly visualized at all imaging time points, even after 6 d.

**Figure 3:**
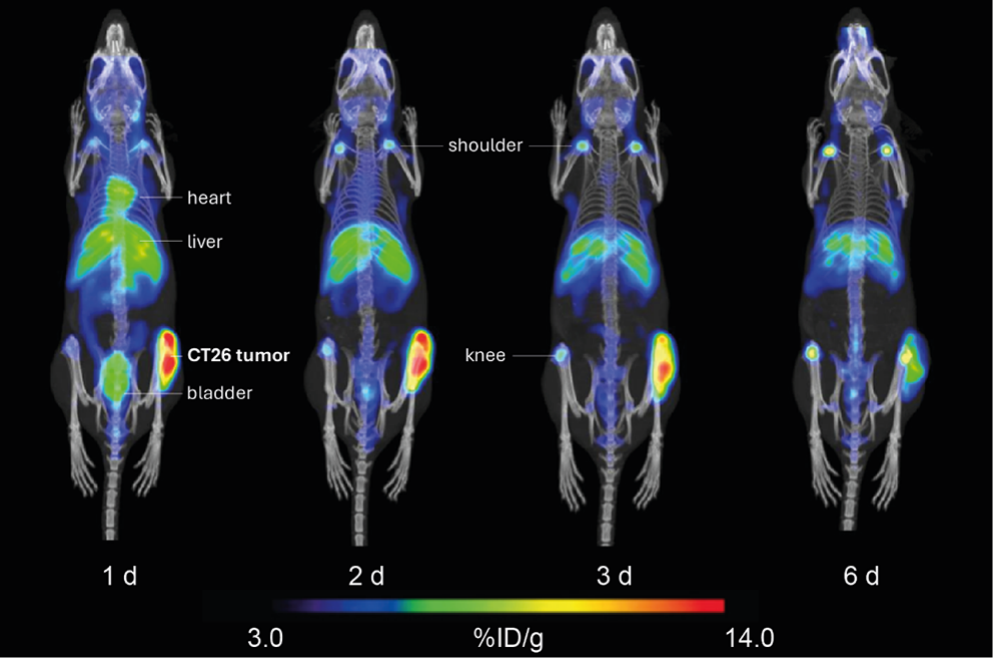
Maximum intensity projection (MIP) PET images fused with CT of Balb/c mouse no. 2 bearing a subcutaneous CT26 tumor are shown for 1, 2, 3, and 6 d p.i. of 9.0 MBq [^89^Zr]­Zr-DFO*-POD-HSA **12.** PET data are decay-corrected for the start of acquisition (data for mouse no. 1 can be found in the SI).

Quantification of the imaging data can be found in the SI (Figures S40 and S41). [^89^Zr]­Zr-DFO*-POD-HSA **12** accumulated to a high degree in the tumor, and the radioactivity was retained for 3 d (8.0 ± 1.1% ID/g 1 d p.i., 9.0 ± 1.1% ID/g 2 d p.i., 8.5 ± 1.6% ID/g 3 d p.i.) but decreased 6 d p.i. (5.5 ± 0.2% ID/g). As expected, the uptake of radioactivity in well-perfused organs and sites of metabolization lowered over time. Unexpectedly, radioactivity also accumulated in the joints over time (knees and shoulders, ∼5–7% ID/g), which is an indication of the release of osteophilic [^89^Zr]­Zr^4+^. Overall, the novel [^89^Zr]­Zr-DFO*-POD-HSA **12** yielded high image contrast for up to 6 d, the longest point reported for PET imaging of tumors with radiolabeled HSA. Studies conducted with primates and a [^89^Zr]­Zr-DFO*-labeled antibody demonstrated the benefit of PET imaging beyond 2–3 physical half-lives of the radioisotope.^57^

### Ex Vivo Biodistribution

For a more precise quantitative assessment of the pharmacokinetic properties of [^89^Zr]­Zr-DFO*-POD-HSA **12**, ex vivo biodistribution experiments with γ-counting of collected organs were also performed. As this is a more sensitive method, a lower amount of radioactivity compared to PET/CT imaging studies could be administered. Thus, eight Balb/c mice bearing CT26-allografts were injected each with 0.4 MBq of [^89^Zr]­Zr-DFO*-POD-HSA **12** (8.5 μg of protein) and groups of animals (*n* = 4) were sacrificed after 1 and 2 d p.i. The biodistribution data for all animals, including previously obtained data for [^89^Zr]­Zr-DFO*malHSA **11** for comparison, are summarized in Figure 4 and Table S2.

**Figure 4:**
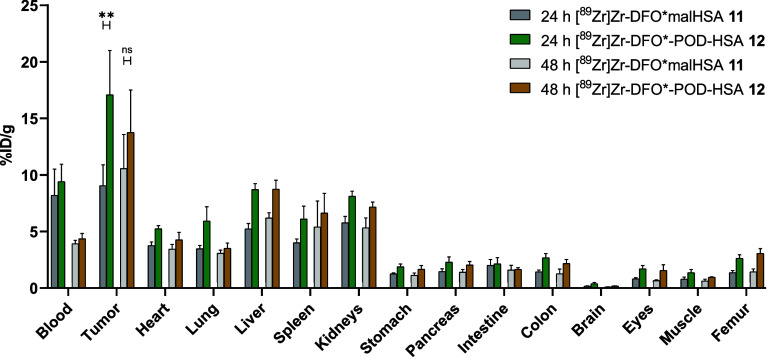
Biodistribution data of [^89^Zr]­Zr-DFO*malHSA **11** and [^89^Zr]­Zr-DFO*POD-HSA **12** in Balb/c mice bearing a subcutaneous CT26 tumor model obtained at 24 and 48 h via γ-counter measurement and calculation of %ID/g tissue. Decay correction was performed for the time of injection. Data for [^89^Zr]­Zr-DFO*malHSA **11** was taken from previously published manuscript to visualize the direct comparison.^35^ Statistical analysis was performed via *t*-test (*p* ≥ 0.05 = ns, *p* < 0.05 = *, *p* < 0.01 = **, *p* < 0.001 = ***, *p* < 0.0001 = ****).

Overall, biodistribution results followed a pattern similar to that observed by in vivo PET imaging (compare Figures 4, S40 and S41). Significant uptake of radioactivity was observed in the blood and highly perfused organs, as well as the excretory organs, which decreased over time. We saw very high tumor uptake of 17.1 ± 3.4% ID/g 1 d p.i. and 13.8 ± 3.2% ID/g 2 d p.i. (*n* = 4 each, ns). Compared to the biodistribution data of [^89^Zr]­Zr-DFO*malHSA **11**, we observed overall a higher uptake of [^89^Zr]­Zr-DFO*-POD-HSA **12** in the majority of the organs. Tumor uptake was significantly higher after 1 d (9.1 ± 1.8 (**11**) vs 17.1 ± 3.4% ID/g (**12**), *n* = 4, **=*p* < 0.01) and similar after 2 d (10.6 ± 3.0 (**11**) vs 13.8 ± 3.2% ID/g (**12**), *n* = 4, ns). Higher uptake in the femur confirmed the observed uptake of radioactivity in joints by PET imaging in the case of [^89^Zr]­Zr-DFO*-POD-HSA **12**. Tumor/tissue ratios were similar with better tumor/blood ratios for the novel radiotracer **12** (Figure S42).

Tumor visualization with radiolabeled albumin by the nuclear imaging modalities PET and SPECT has been reported by different groups.^56,58–64^ Even though the results of the published studies vary, tumor uptake (approximately 4–9% ID/g p.i.) was sufficient for imaging in all cases. The studies differ in terms of the used radionuclide, chelator, bioconjugation strategy, or animal model. Therefore, a direct comparison with our work should be done with care.

The higher tumoral uptake of [^89^Zr]­Zr-DFO*-POD-HSA **12** compared to that of [^89^Zr]­Zr-DFO*malHSA **11** could be attributed to the higher stability of the former in serum (in vitro). Conversely, the argument of higher stability does not correlate with the observed unspecific resorption of released osteophilic radiometal [^89^Zr]­Zr^4+^ in bones and joints.^65^ The stability of [^89^Zr]­Zr-DFO* in vivo has been demonstrated multiple times^38^ and, therefore, a possible explanation for the observed uptake of radioactivity in bones could be that the POD linkage between the radiometal complex and HSA decreases it is in vivo stability.

Analogous to our previous report,^35^ we determined the in vivo plasma stability of [^89^Zr]­Zr-DFO*-POD-HSA **12** in murine blood serum via spin filtration assay. Thereby, no low-molecular weight fraction (<30 kDa) of radiolabeled compounds was observed, indicating an intact radiotracer in blood after 24 and 48 h p.i. Thus, the POD-linkage of the radioconjugate appears stable in vivo (no detectable [^89^Zr]­Zr-DFO*-complex), however, free ^89^Zr^4+^ is released nevertheless, likely due to interference of the POD moiety with the complexation of the radiometal.

## Conclusion

Three different Cys-specific bioconjugation strategies for the chemoselective and, in the case of HSA site-selective, ^89^Zr-radiolabeling of the protein were investigated and compared side-by-side. Suitable BFCAs of the ^89^Zr-chelator DFO* incorporating thiol-reactive mal, PODS, and VK were synthesized, conjugated to Cys^34^ of HSA, and radiolabeled with ^89^Zr-oxalate. While the reactivity of the three functional groups toward thiols was comparable with model compounds in vitro, their bioconjugation efficiency to HSA differed significantly even under optimized reaction conditions, with mal > PODS > VK. However, the achieved ratio of chelator-to-protein was sufficient for radiotracer development in all cases. Radiolabeling of the three DFO*-HSA conjugates, identical in all respects except the linkage to HSA, with [^89^Zr]­Zr-oxalate was achieved under optimized ^89^Zr-labeling conditions. The achieved RCY and A_S_ varied, which reflected the chelator-to-protein ratios of the [^89^Zr]­Zr-DFO*-HSA conjugates. All ^89^Zr-labeled conjugates were obtained with a RCP > 95% suitable for biological experiments. Stability studies of the [^89^Zr]­Zr-DFO*-HSA conjugates revealed good stability of the mal- and POD-conjugates in serum, but only [^89^Zr]­Zr-DFO*-POD-HSA **12** was stable in cell culture medium and therefore suitable for in vitro experiments. Unlike published data on VK-based bioconjugates, the stability of [^89^Zr]­Zr-DFO*VK-HSA **13** was found to be insufficient for biological experiments. [^89^Zr]­Zr-DFO*-POD-HSA **12** was investigated in Balb/c mice bearing CT26-allografts by PET/CT imaging and biodistribution studies and compared with previously published data on [^89^Zr]­Zr-DFO*malHSA **11**. The two radiotracers showed comparable characteristics in vivo with good uptake of radioactivity in tumors (up to 17% ID/g) and low background at >1 d p.i. (tumor-to-blood ratio 3.2 ± 1.0 at 2 d p.i.) resulting in clear visualization of tumors by PET imaging up to 6 d p.i. However, in the case of [^89^Zr]­Zr-DFO*-POD-HSA **12**, an undesirable uptake of radioactivity in bones was observed. The reason for this issue is the subject of investigations currently ongoing in our laboratories. Our study shows that the bioconjugation method should be chosen with care and based on the intended use of the conjugate. In the case of ^89^Zr-labeled HSA, utilizing PODS chemistry is more suitable for in vitro studies, whereas the mal-chemistry-derived conjugates perform better in vivo. In summary, the in vivo performance of [^89^Zr]­Zr-DFO*-labeled HSA obtained by mal chemistry remains so far the most promising candidate identified for the development of a clinical companion diagnostic PET imaging probe for the stratification of patients for therapies based on HSA-binding drugs and prodrugs.

## Experimental Procedures

### Synthesis of BFCAs

Synthesis procedures are provided in the SI.

### Kinetic Studies

5 mM stock solutions of **2**, **3**, and **4** were prepared using DMSO. A 5 mM stock solution of **14** was prepared using water. 10 μL of the linker solution was diluted with 180 μL 50 mM NaOAc (pH = 5.5). After an equimolar addition of 10 μL of **14**, the solutions were filtered (0.22 μm syringe filter) and transferred to an HPLC vial. The vial was placed immediately into the autosampler at 37 °C. Measurements were conducted every 10 min.

### Bioconjugation

**8** was prepared as previously described by us.^35^ For bioconjugates **9** and **10** the respective DFO* derivative **6** or **7** was prepared as a 2 mM stock solution in DMSO. 100 μL (200 nmol, 4 equiv) was propounded in a 2 mL low-binding plastic tube. PBS (pH = 7.4) or NaOAc (pH = 5.5) was added to yield a final DMSO concentration of <10%w/w and mixed thoroughly. At last, HSA in PBS (50 nmol, 66 mg/mL, 1 equiv) was added and the reaction was kept at 37 °C for 0.75–1.5 h. Every 15–30 min, the tubes were inverted to ensure sufficient mixing of the reactants. In a first step, the crude reaction solution was purified via PD-10 column to 3 mL water or 0.9% NaCl, according to manufacturer’s instructions. As a second purification step, the product was washed three times via spin filtration (Amicon 4, 30 kDa MWCO, 4000 *g*, 15 min, 20 °C) using water or 0.9% NaCl. If the bioconjugate was reformulated in water, it was lyophilized and obtained as white solid with >85% yield. In case of reformulation of the bioconjugate in 0.9% NaCl, aliquots were kept frozen at −20 °C. The conjugates were analyzed via SEC and an isotopic dilution assay (see SI).

### Radiolabeling

Radiolabeling of the HSA conjugates **8**, **9**, and **10** was performed according to modified procedures.^37^ 5–25 MBq [^89^Zr]­Zr­(oxalate)_2_ in 1 M oxalic acid were diluted to achieve a total volume of 50 μL 1 M oxalic acid. 22.5 μL of 2 M Na_2_CO_3_ was used to neutralize the solution, which was then further diluted with the respective radiolabeling buffer (0.5 M HEPES pH = 7.4 or 1 M NH_4_OAc pH = 7.0), followed by 0.5 mg of conjugate (in 0.9% NaCl), to achieve a total volume of 500 μL. The reaction was incubated at 37 °C while shaking at 300 rpm for 3–24 h. 30 μL of 25 mg/mL EDTA was added and after 5 min the crude radiolabeling solution was purified in a first step via PD-10 column to 3 mL 0.9% NaCl. Subsequently, the radiotracer was further purified via three circles of spin filtration (Amicon 4, 30 kDa MWCO, 4000 *g*, 15 min, 20 °C) in 0.9% NaCl to obtain **11**, **12** and [^89^Zr]­Zr- **13**. For animal experiments, 134.6 MBq of [^89^Zr]­Zr­(oxalate)_2_ in 1 M oxalic acid was used for 1 mg of **9**. The volumes of all used solutions were doubled, the reaction was kept for 16 h, and the purification was done analogously to the other radiolabeling reactions. Quality control included iTLC (performed on silica gel impregnated paper with 50 mM EDTA solution as mobile phase), SEC and a spin filtration assay for the determination of the RCP.^37^ For the latter, 2–10 μL of radiolabeling solution was diluted to 100 μL with washing buffer, consisting of 0.9% NaCl and 5% DMSO. The solution was pipetted into a centrifugal filter unit (Microcon Centrifugal Filter Unit with Ultracel-30 membrane and 30 kDa MWCO), which was subsequently centrifuged at 14000 *g* for 7 min. Afterward, the filter was washed two times with 100 μL of washing buffer. The combined filtrates and filter were counted separately in a γ-counter and corrected for background and decay. The radiochemical purity was determined as the ratio of counts detected for the filter to the total number of counts.

### Stability

To 0.3–1 MBq of radiotracer solution 0.9% NaCl was added to yield a total volume of 50 μL. The solution was added to 450 μL of 0.9% NaCl, human blood serum (Dunn Labortechnik), RPMI-1640 cell culture medium, PBS, 0.5 M HEPES buffer (pH 7.4), or 1 M NH_4_OAc buffer (pH = 7.0), respectively. The solutions containing 0.9% NaCl and human blood serum were kept at 37 °C over the course of 7 d (*n* = 3). Samples were taken after 1 h, 2 h, 4 h, 1 d, 2 d, 3 d, and 7 d and analyzed for their RCP via spin filter analysis. All other investigated solutions were kept at 37 °C over the course of 4 h (*n* = 3). Samples were taken after 1 h, 2 h, and 4 h and analyzed likewise.

### Animal Experiments

Female, 12–15 weeks old Balb/c mice (Envigo, Horst, The Netherlands) were used for the animal experiments in this study. All animals were acclimatized to laboratory conditions for at least 1 week prior to the experiments. The animals were housed under standard laboratory conditions in individually ventilated cages on sawdust with free access to animal feed and water. Their general health and body conditions were monitored throughout the experiments. The number of animals used in all in vivo experiments was reduced as much as possible (*n* = 4 or 2 per group and time point). To minimize suffering of the animals and reduce movement artifacts, injections and imaging studies were performed under 2% isoflurane anesthesia (FORANE, Abbott Laboratories, Abbott Park, IL, USA). All animal experiments were conducted in accordance with the regulations and guidelines of the Czech Animal Protection Act (No. 246/1992) and were approved by the Czech Ministry of Education, Youth and Sports (MSMT-24421/2021-4 and MSMT-20283/2024-3) and the Institutional Animal Welfare Committee of the Faculty of Medicine and Dentistry at Palacký University Olomouc. For the allografts, CT26 cells (5 × 10^5^ in 100 μL of RPMI medium) were subcutaneously injected into the right flank of female Balb/c mice (*n* = 10). After 8 days of tumor growth, animals were randomized and used for experiments including ex vivo biodistribution study or PET/CT imaging.

### Longitudinal PET/CT Imaging

Two mice bearing sc CT26 allografts (tumor volume 200–300 mm^3^, as determined by caliper measurement) were injected iv with 10.4 (266 μg HSA) and 9.0 MBq (231 μg HSA), respectively, of **12** in 0.9% NaCl. The animals were put under 2% isoflurane anesthesia and placed in the prone position in a Mediso NanoScan PET/CT small animal imaging system (Mediso Medical Imaging Systems, Budapest, Hungary). Static imaging was performed 1, 2, 3, and 6 d after the administration of **12**. Single field of view (FOV) (98.5 mm) 10 min PET scan was performed, followed by whole body helical CT scan (50 kVp/980 μA, 720 projections). Image reconstruction was performed by using Mediso Tera-Tomo 3D PET iterative reconstruction software (Mediso Medical Imaging Systems, Budapest, Hungary). Image visualization, analysis, processing, and quantification were performed using Mediso InterView FUSION software (Mediso Medical Imaging Systems, Budapest, Hungary). All scans were normalized to injected activity and animal weight and decay-corrected for the start of the acquisition. Quantitative analysis of activity distribution in joints and organs was performed by measuring the percentage injected dose per gram (%ID/g) within a region of interest (ROI). The ROIs were drawn based on the anatomical structures visualized by CT scans. The results were expressed as %ID/g.

### Ex Vivo Biodistribution

Eight mice bearing sc CT26 allografts (mean tumor volume 200–300 mm^3^, as determined by caliper measurement) were injected iv with 0.4 MBq of **12** in 0.9% NaCl via the tail vein. The injection volume did not exceed 150 μL per intravenous application. The animals were sacrificed by cervical dislocation at different time points (24 and 48 h p.i., *n* = 4 per time point). The organs were collected, weighed, and measured in a γ-counter. Counts per minute were decay-corrected and normalized to weight and injected dose, and the biodistribution data were expressed as the percentage of the injected dose per gram of tissue (%ID/g). Blood samples of all animals were taken directly after sacrifice and collected in Tween80 coated 1.5 mL tubes. Samples were weighted, and the radioactivity was measured in a γ-counter, analogous to all other biodistribution samples.

### Statistical and Data Analyses

All of the statistical analyses were performed using GraphPad Prism 8. Data were analyzed using either *t*-tests (comparison of different radiotracers) or paired *t-*tests (comparison of different time points). All of the presented graphs include error bars that denote the standard deviation of the mean values.

## Supplementary Material



## Notes

General materials and methods. Synthesis procedures of compounds **1**, **6**, **7**, **14**, and **S1–S7**, including RP-HPLC-MS chromatograms and 1D and 2D NMR spectra (Scheme S1, Figures S1–S25). Results of kinetic studies (Figures S26–S29). Procedures for DTNB assay, reduction of HSA, and isotopic dilution assay. SE-chromatograms of bioconjugates **8–10** (Figures S30–S32) and radiotracers **11–13** (Figures S34–S36). Additional data of isotopic dilution assays (Table S1). Schematic representation of the quality control methods required for comprehensive characterization of HSA-based radiotracers (Figure S33). Stability data for up to 7d of incubation (Figure S37). Cellular uptake of compound **12** (procedure and Figure S38). Additional data on animal experiments (Figures S39–S42, Table S2) (PDF)
