# Supplementary material for: Side-by-Side Comparison of Different Thiol Bioconjugation Strategies for the Chemoselective Radiolabeling of Human Serum Albumin with Zirconium-89
Source: ACS Bio Med Chem Au. 2026 Apr 20;6(3):282–92. doi: 10.1021/acsbiomedchemau.6c00002 (PMC13281002; doi:10.1021/acsbiomedchemau.6c00002)
Supplement: Supplementary file 1 [file bg6c00002_si_001.pdf]

Supporting Information

Side-by-side comparison of different thiol bioconjugation strategies for the chemo-selective radiolabeling of human serum albumin with zirconium-89

Julia Kronberger[a,b,c], Barbora Neuzilova[d], Anja Federa[a,b], Manuel Tieber[a], Amando Palombo[a], Marie R. Brandt[a,c], Petra Heffeter[e], Christian R. Kowol[a], Milos Petrik[d,f,g], Thomas L. Mindt*[a,c]

[a] Institute of Inorganic Chemistry, Faculty of Chemistry, University of Vienna, Währinger Straße 42, 1090 Vienna, Austria

[b] Vienna Doctoral School in Chemistry, University of Vienna, Währinger Straße 42, 1090 Vienna, Austria

[c] Joint Applied Medicinal Radiochemistry Facility, University of Vienna, Medical University of Vienna, 1090 Vienna, Austria

[d] Institute of Molecular and Translational Medicine, Faculty of Medicine and Dentistry, Palacký University, 779 00 Olomouc, Czech Republic

[e] Center for Cancer Research and Comprehensive Cancer Center, Medical University of Vienna, Borschkegasse 8a, 1090 Vienna, Austria

[f] Institute of Molecular and Translational Medicine, University Hospital, 779 00 Olomouc, Czech Republic

[g] Czech Advanced Technology and Research Institute, Palacký University, 779 00 Olomouc, Czech Republic

* Corresponding author: Thomas L. Mindt, e-mail: thomas.mindt@univie.ac.at

Table of Contents

I. General. S3

II. Mass spectrometry. S3

III. Chromatography. S3

IV. Chemical synthesis S4

V. Kinetics S23

VI. DTNB assay. S25

VII. Reduction of Cys34 of HSA. S25

VIII. Isotopic dilution assay. S25

IX. Size exclusion chromatograms of DFO*-HSA derivatives S26

X. Additional data of isotopic dilution assay S27

XI. Schematic representation of the quality control methods S27

XII. Size exclusion chromatograms of radiolabeled DFO*-HSA derivatives S28

XIII. Cell culture. S29

XIV. Cellular uptake of radiolabeled DFO*-HSA derivatives S29

XV. Animal data S30

I. General.

For the syntheses of all precursor compounds and DFO* derivatives all chemicals were purchased from commercial suppliers (Acros Organics, Alfa Aesar, Fisher Scientific, Fluka, Sigma Aldrich, TCI and VWR) and used without purification. Solvents were obtained by Merck or Fisher Scientific. Silica gel (particle size 40−63 µm) for column chromatography was purchased from VWR. DFO*mal was obtained from ABX (Radeberg, Germany). HSA (Albunorm©) was purchased from Octapharma (Lachen, Switzerland) and reformulated to PBS (pH 7.4, Mephisto) via PD-10 column before use (1 mL of 200 mg/mL stock solution applied, collected in 3 mL PBS). All radiolabeling buffers were prepared using Millipore water pre-treated with Chelex© resin (Chelex© 100 sodium form (50–100 mesh, Sigma Aldrich), to remove trace metals, for 15 min and then filtered to remove the Chelex© beads (50 g/L). All pipette tips and reaction tubes (Eppendorf®) used for handling or storing protein samples containing <0.5 mg/mL protein were pre-coated with 0.1% Tween® 80 in 0.9% NaCl (Braun). Quantification of protein concentration was determined using a microvolume UV/VIS spectrophotometer (NanoDrop One^{C}, Thermo Fisher Scientific). The protein concentration was calculated by the measured absorbance at λ = 280 nm, HSA molecular weight of 66 437 Da and the molar extinction coefficient of ε = 35 495 $M^{-1}cm^{-1}$.[1] Gamma counting was performed on a 2480 Wizard2 1-Detector γ-counter with an energy window of 500 - 1000 keV (crystal: NaI (Tl), 80 mm in height, 75 mm in diameter; Perkin Elmer). Sample radioactivity was measured with a VDC-405 dose calibrator V3.26 (Veenstra) calibrated for ^{89}Zr. Nuclear magnetic resonance (NMR) measurements were recorded at 25°C on a Bruker Avance III 500 MHz spectrometer, Bruker AV NEO 500 spectrometer, Bruker FT-NMR AV III 600 spectrometer or Bruker AV III HD 700 spectrometer. All NMR spectra were measured in deuterated dimethyl sulfoxide (DMSO-d6 purchased from Eurisotop). The respective residual solvent peak was used as internal reference for the chemical shifts (ppm). Spin multiplicities are abbreviated as follows: s=singlet, d=doublet, t= triplet, q=quartet, m=multiplet, bs=broad signal. The values of the coupling constants (J) are given in Hertz (Hz). High-resolution mass spectra (HRMS) were measured on an Orbitrap Exploris™ 120 mass spectrometer. For centrifugation a ROTINA 380/380R from Hettich was used. Microwave reactions were performed with a Biotage Initiator+ system. The device has a range of power up to 400 W and needed about 1 min to heat to the desired temperature.

II. Mass spectrometry.

HR-ESI-MS spectra (m/z 50–1900) were obtained on a maXis UHR ESI-Qq-TOF mass spectrometer (Bruker Daltonics, Bremen, Germany) in the positive-ion mode by direct infusion. The sum formulas of the detected ions were determined using Bruker Compass DataAnalysis 4.1 based on the mass accuracy (Δm/z≤5 ppm) and isotopic pattern matching (SmartFormula algorithm). Mass spectra of protein conjugates were obtained using LTQ Orbitrap Velos mass spectrometer (Thermo Fisher Scientific, Bremen, Germany) equipped with a nanospray ion source, coupled to the nano HPLC-system (UltiMate 3000, Dionex).

III. Chromatography.

Reversed phase high-performance liquid chromatography (RP-HPLC) runs were performed using a Waters Acquity UPLC® BEH C18 column (130Å, 1.7 µm, 3 mm x 50 mm) on a Dionex Thermo Scientific UltiMate 3000 HPLC system, equipped with an HPG-3400RS binary pump and a DAD-3000 UV-VIS detector. Milli-Q water (mobile phase A) and acetonitrile (ACN, mobile phase B), both containing 0.1% TFA, were used as eluents. The flow rate was consistent at 0.6 mL/min for all measurements. Chromatograms were evaluated at 220 nm wavelength. RP-HPLC-MS analyses were conducted using the same Waters Acquity UPLC® BEH C18 column on an Agilent 1260 Infinity II system equipped with a Flexible pump, a 1260

VWD UV-Vis detector and the LC–MSD system. Mobile phases A (Milli-Q) and B (acetonitrile, ACN) were containing 0.1% formic acid (FA) each. The flow rate was consistent at 0.6 mL/min for all measurements. Chromatograms were evaluated at 220 nm wavelength. For preparative RP-HPLC, a Waters XBridge BEH C18 OBD Prep Column (130 Å, 10 µm, 19 mm x 250 mm) was used on an Agilent 1200 Series system. The methods used are described in the respective protocol. SEC was performed on an NGC Quest 10 Plus Chromatography System (Bio-Rad), with integrated single wavelength UV detector (255 or 280 nm) and conductivity unit, additionally equipped with a HERM LB 500 NaI γ-detector (Berthold). Delay volume between UV detector and radiodetector was 0.5 mL. Protein samples were eluted on a BioSep SEC-s2000 size-exclusion column (Phenomenex) with PBS (pH 7.4, Mephisto) at a flowrate of 1 mL/min. Radio-TLC was performed on silica gel impregnated paper (Agilent) with 50 mM EDTA solution as mobile phase. After the development of the TLC plate, it was dried with a heating gun and the radioactivity on the plate was measured with a miniGITA dual device (Elysia-raytest) with 2 mm collimator height, detecting 500 – 1000 keV (measuring time: 1-8 min) or by cutting the TLC plates in half and measuring the radioactivity in γ-counter.

IV. Chemical synthesis

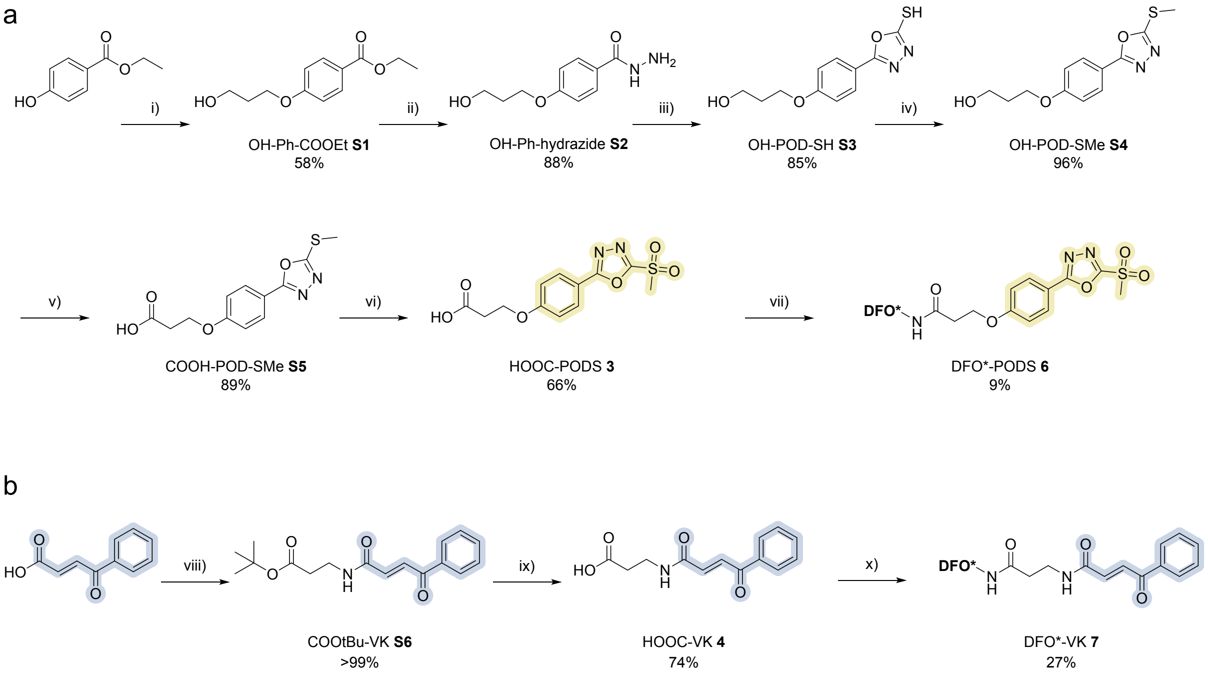

Scheme S 1 Reaction schemes of the synthesis of novel cysteine-selective DFO* derivatives: a) DFO*-PODS **6** and b) DFO*-VK **7**. Conditions: i) 3-iodo-1-propanol, K_2CO_3 in dry DMF, 75°C (yield: 58%); ii) $N_2H_2 \cdot H_2O$ in EtOH, reflux (yield: 88%); iii) CS_2, KOH in dry DMF/EtOH (2:1), 100°C (yield: 85%); iv) CH_3I, NEt_3 in dry THF, 0°C–r.t. (yield: 96%); v) Jones reagent (CrO_3, H_2SO_4, H_2O) in acetone, 0°C–r.t. (yield: 89%); vi) $(NH_4)_6Mo_7O_{24} \cdot 4H_2O$, H_2O_2 in EtOH, 0°C–r.t. (yield: 66%); vii) [DFO*NH_2]TFA, HATU, DIPEA in DMF, r.t.–50°C microwave (yield: 9%); viii) *tert*-butyl 3-aminopropanoate·HCl, isobutyl chloroformate, *N*-methylmorpholine in dry dimethoxyethane/DMF (1:1), 0°C–r.t. (yield: quant.); ix) TFA in DCM, r.t. (yield: 74%); x) [DFO*NH_2]TFA, HATU, DIPEA in DMF, r.t.–50°C microwave (yield: 27%).

*[DFO*NH$_2$]TFA* ***1**.*

[DFO*NH$_2$]TFA was synthesized as described earlier.[2] Adaptions were made for the purification of the final product. Instead of purifying the final product via SepPak cartridges, we used preparative HPLC to obtain larger quantities. The method used a flow rate of 6 mL/min, mobile phase A=H_2O+0.1% TFA, mobile phase B=MeOH+0.1% TFA, 44% B isocratic, 17.1 min product peak). The fractions containing the product were pooled and lyophilized to yield [DFO*NH$_2$]TFA **1** as a white solid (20% yield, 97-99% (HPLC)). The final product was characterized by RP-HPLC-MS, ^{1}H-, ^{13}C-, COSY-, HSQC- and HMBC-NMR. NMR data was included as the signal assignment has not been published yet.

RP-HPLC-MS: Gradient = 0.5-6.0 min 5-95% B, UV (λ=220nm) product peak at 2.79 min, m/z: $[M+H]^+$ calcd for $C_{34}H_{64}N_8O_{11}$ 761.5; found 761.6.

^{1}H-NMR (500.10 MHz, DMSO-d6) δ 7.79 (q, J=5.1 Hz, 3H), 3.52–3.42 (m, 8H), 2.99 (q, J=6.6 Hz, 6H), 2.75 (t, J=7.5 Hz, 2H), 2.58 (dt, J=10.3, 5.0 Hz, 6H), 2.36–2.20 (m, 6H), 1.96 (s, 3H), 1.50 (q, J=
7.9 Hz, 10H), 1.37 (m, 6H), 1.31–1.12 (m, 8H).

^{13}C-NMR (151 MHz, DMSO, DEPTq135) δ 171.93, 171.25, 170.09, 47.04, 46.80, 46.74, 40.03, 39.89, 39.75, 38.87, 38.39, 29.85, 29.75, 28.78, 27.52, 27.42, 26.92, 25.99, 25.71, 23.46, 22.86, 20.32.

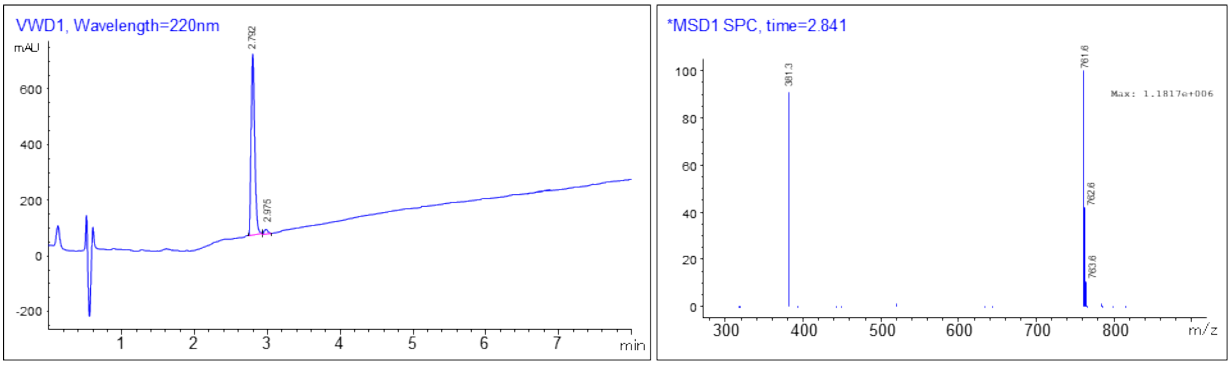

Figure S 1 RP-HPLC-MS of [DFO*NH$_2$]TFA **1**. Gradient: 0.5-6.0 min 5-95% B. Left: UV-chromatogram (λ=220nm). Right: MS spectrum of product peak.

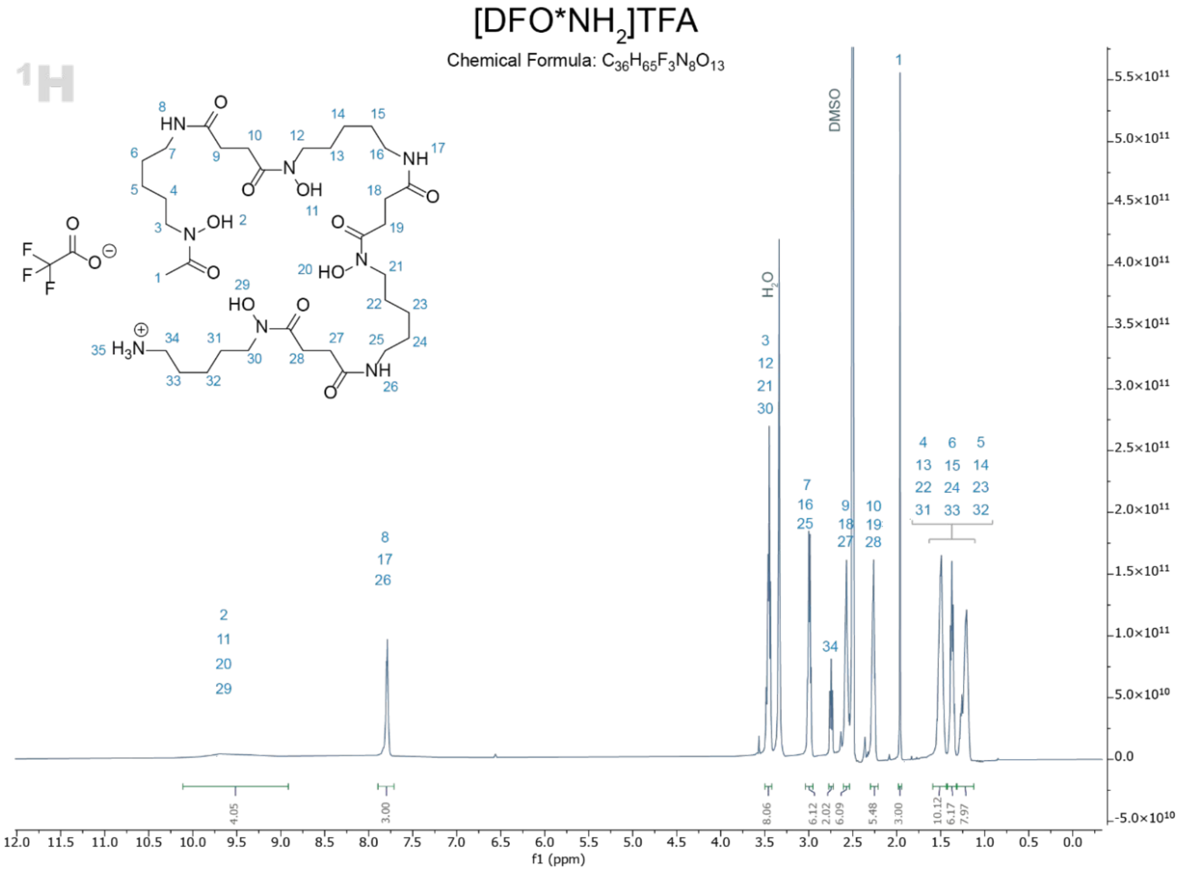

Figure S 2 ^{1}H-NMR (500.10 MHz, DMSO-d6) spectrum of [DFO*NH$_2$]TFA **1**.

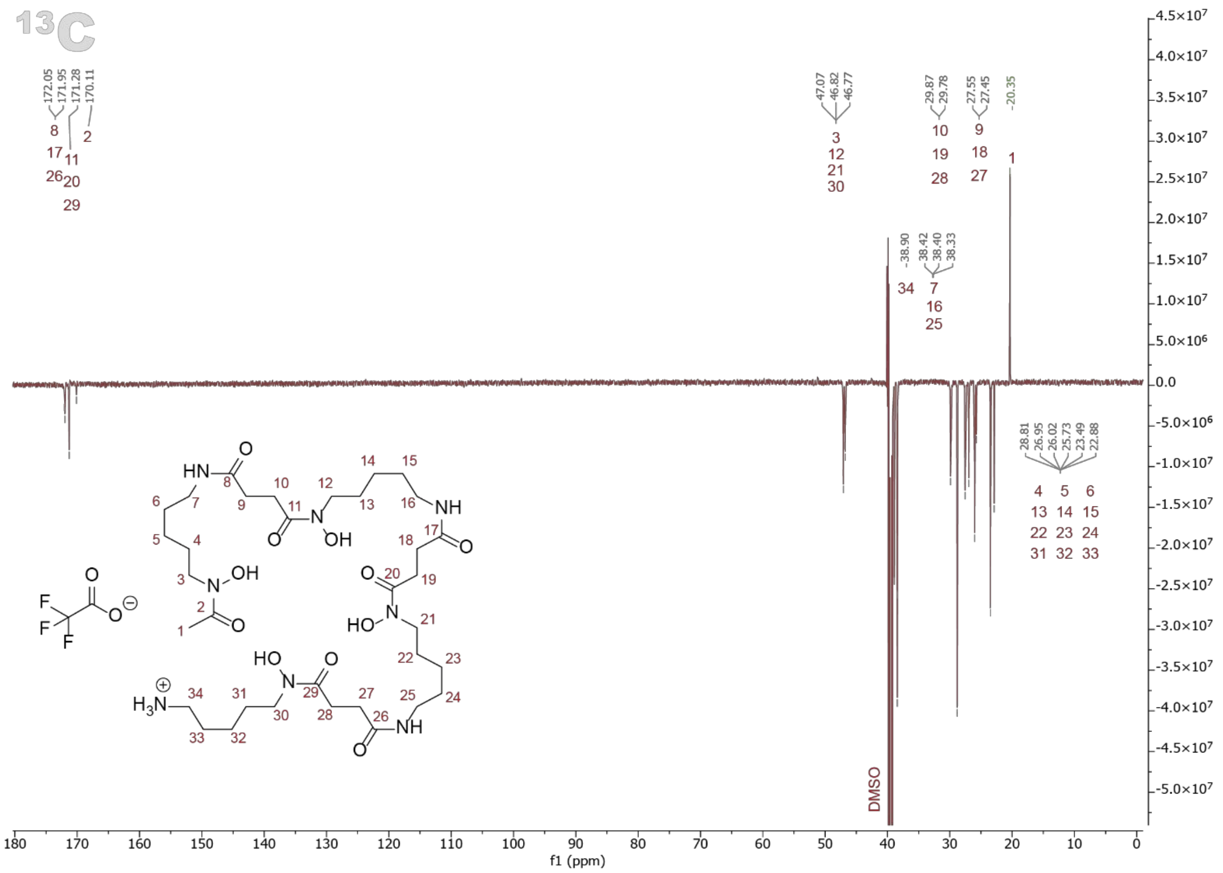

Figure S 3 ^{13}C-NMR (151 MHz, DMSO, DEPTq135) spectrum of [DFO*NH$_2$]TFA **1**.

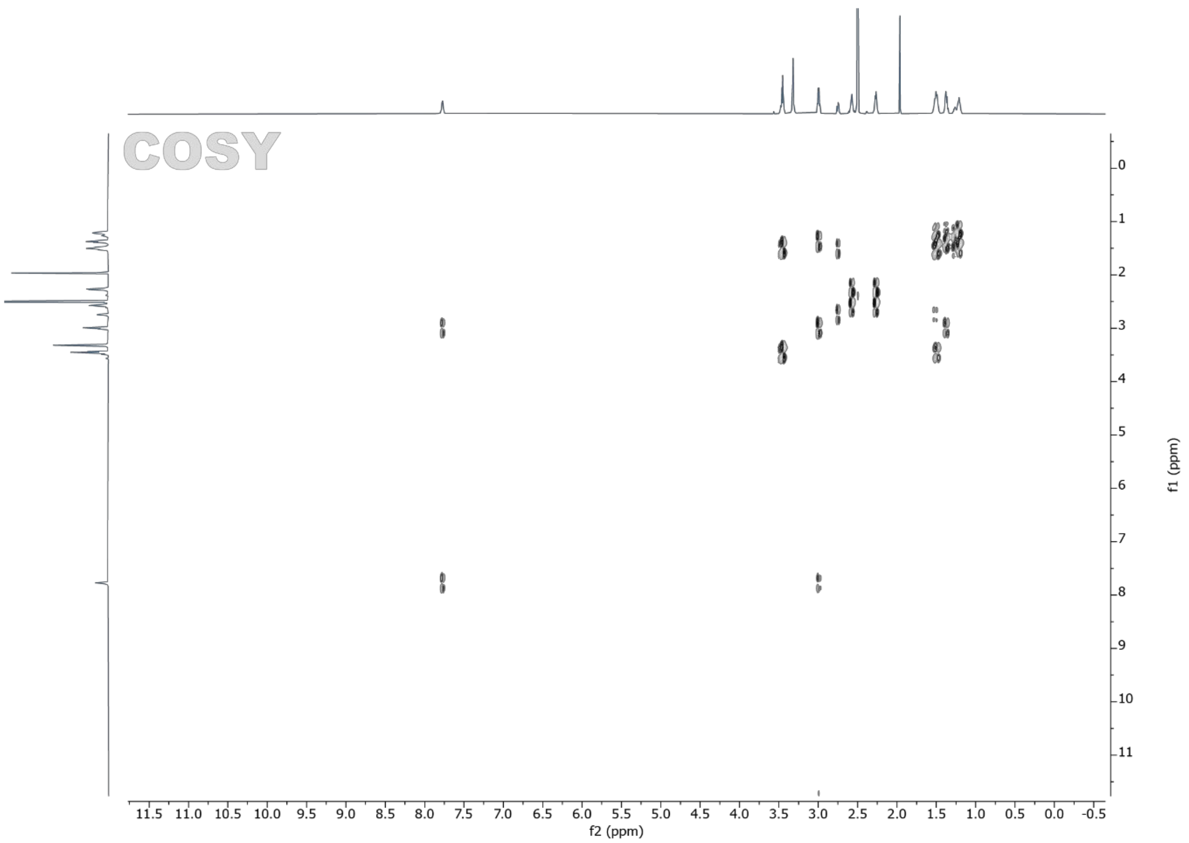

Figure S 4 COSY-NMR (DMSO-d6) spectrum of [DFO*NH_2]TFA **1**.

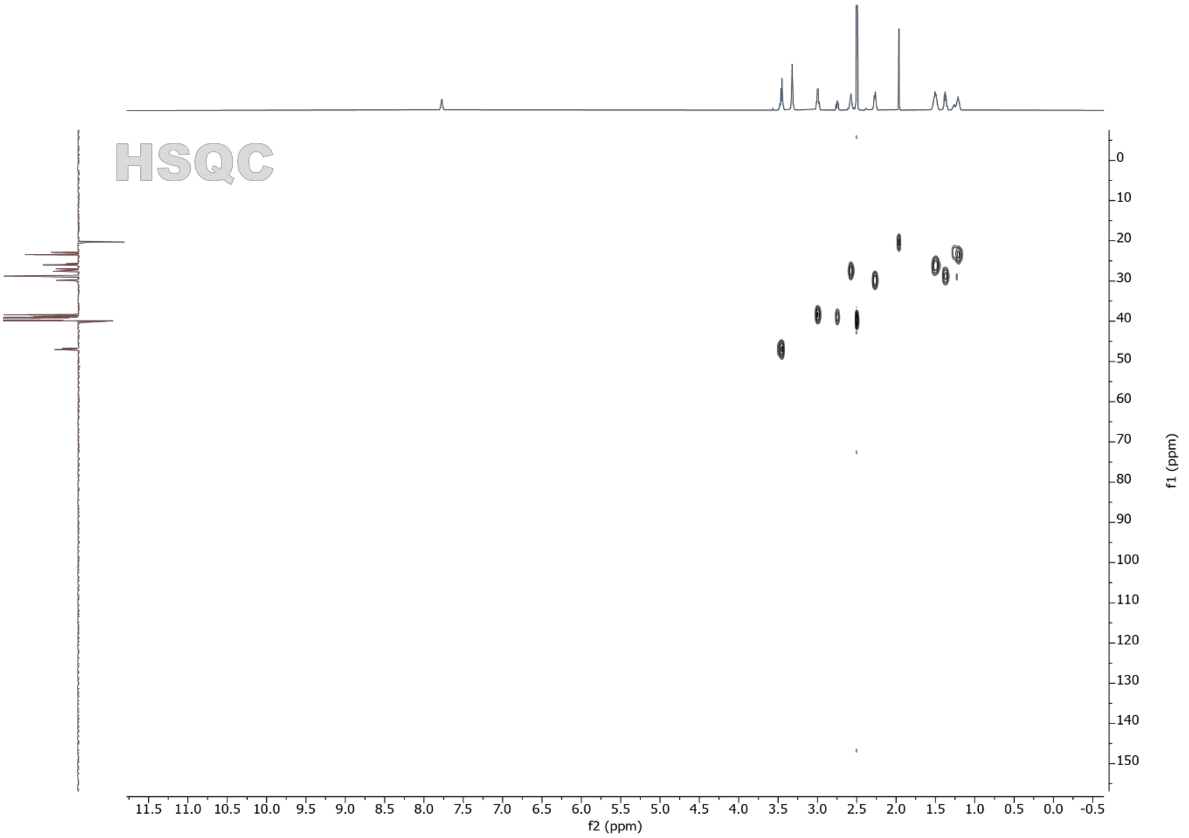

Figure S 5 HSQC-NMR (DMSO-d6) spectrum of [DFO*NH_2]TFA **1**.

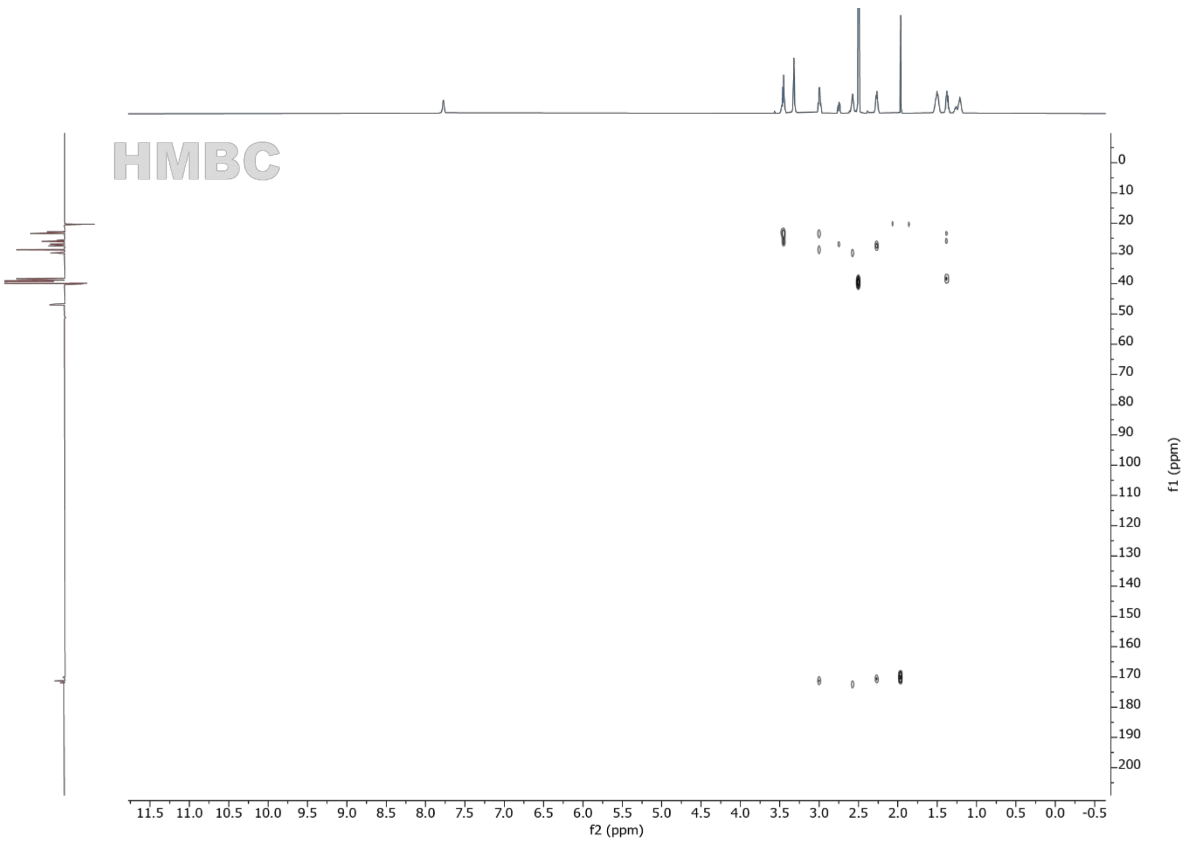

Figure S 6 HMBC-NMR (DMSO-d6) spectrum of [DFO*NH$_2$]TFA **1**.

*OH-Ph-COOEt **S1**.*

Ethyl 4-hydroxybenzoate (350 mg, 2.09 mmol, 1 equiv.) and K_2CO_3 (728 mg, 5.21 mmol, 2.5 equiv.) were suspended in 16 mL dry DMF and 3-iodo-1-propanol (400 mg, 2.09 mmol, 1 equiv.) was added. The mixture was heated to 75°C and stirred for 24 h. After cooling to room temperature, the mixture was poured over 40 mL ice water and extracted 3x with 40 mL diethylether. The combined organic phases were dried over $MgSO_4$ and concentrated *in vacuo* to give a crude yellow oil, which was purified *via* silica column chromatography using a 1:1 mixture of EtOAc and n-hexane as the eluent. After solvent evaporation, the product OH-Ph-COOEt **S1** was obtained as a clear oil (269 mg, 1.20 mmol, 58% yield). ^{1}H NMR (500 MHz, DMSO-d_6): δ 7.93–7.86 (m, 2H), 7.07–6.99 (m, 2H), 4.57 (t, J = 5.2 Hz, 1H), 4.27 (q, J = 7.1 Hz, 2H), 4.11 (t, J = 6.4 Hz, 2H), 3.55 (q, J = 5.8 Hz, 2H), 1.87 (p, J = 6.3 Hz, 2H), 1.30 (t, J = 7.1 Hz, 3H).

*OH-Ph-hydrazide **S2**.*

OH-Ph-COOEt **S1** (268 mg, 1.19 mmol, 1 equiv.) was dissolved in 4 mL EtOH and hydrazine monohydrate (1.25 mL, 25.25 mmol, 21 equiv.) was added. The mixture was stirred at reflux for 48 h, then all volatiles were removed *in vacuo*. The residue was triturated in a 1:1 mixture of EtOAc and n-hexane, filtered and the filter washed 3x with the same mixture. The product OH-Ph-hydrazide **S2** was obtained as a white solid (221 mg, 1.05 mmol, 88% yield). ^{1}H NMR (500 MHz, DMSO-d_6): δ 9.59 (s, 1H), 7.78 (d, J = 8.2 Hz, 2H), 6.96 (d, J = 8.3 Hz, 2H), 4.55 (t, J = 4.5 Hz, 1H), 4.42 (s, 2H), 4.07 (t, J = 5.9 Hz, 2H), 3.61–3.49 (m, 2H), 1.86 (p, J = 6.0 Hz, 2H).

*OH-POD-SH **S3**.*

OH-POD-hydrazide **S2** (218 mg, 1.04 mmol, 1 equiv.) and potassium hydroxide (69 mg, 1.04 mg, 1 equiv.) were dissolved in 2 mL dry EtOH and 1 mL dry DMF and carbon disulfide

(251 µL, 4.15 mmol, 4 equiv.) was added. The mixture was stirred at room temperature for 15 min, then heated to 100°C and stirring was continued for 21 h. All volatiles were removed in vacuo and the residue suspended in 10 mL dH_2O. The pH was adjusted to 1 using 1 M HCl and the aqueous phase subsequently extracted 3x with 15 mL EtOAc. The combined organic phases were dried over Na_2SO_4 and concentrated *in vacuo*. The residue was triturated in a 1:1 mixture of EtOAc and n-hexane, filtered and the filter washed with the same mixture. The product OH-POD-SH **S3** was obtained as an ochre-colored solid (222 mg, 0.88 mmol, 85% yield). ^{1}H NMR (500 MHz, DMSO-d_6): δ 14.61 (s, 1H), 7.81 (d, *J* = 8.7 Hz, 2H), 7.12 (d, *J* = 8.8 Hz, 2H), 4.57 (bs, 1H), 4.13 (t, *J* = 6.3 Hz, 2H), 3.56 (t, *J* = 6.1 Hz, 2H), 1.88 (p, *J* = 6.2 Hz, 2H).

*OH-POD-SMe **S4**.*

OH-POD-SH **S3** (218 mg, 0.86 mmol, 1 equiv.) was dissolved in 10 mL dry THF and triethylamine (145 µL, 1.04 mmol, 1.2 equiv.) was added. The mixture was cooled to 0°C and stirred for 10 min. Iodomethane (60 µL, 0.95 mmol, 1.1 equiv.) was added and the mixture was stirred for another 10 min in the cold, then at room temperature for 3 h. 15 mL of dH_2O were added and the product was extracted 3x with 15 mL EtOAc. The combined organic phases were dried over Na_2SO_4 and concentrated *in vacuo*. The product OH-POD-SMe **S4** was obtained as an ochre-colored solid (222 mg, 0.83 mmol, 96% yield). ^{1}H NMR (500 MHz, DMSO-d_6): δ 7.89 (d, *J* = 8.7 Hz, 2H), 7.12 (d, *J* = 8.8 Hz, 2H), 4.58 (t, *J* = 5.1 Hz, 1H), 4.13 (t, *J* = 6.3 Hz, 2H), 3.56 (q, *J* = 5.7 Hz, 2H), 2.75 (s, 3H), 1.88 (p, *J* = 6.3 Hz, 2H).

*COOH-POD-SMe **S5**.*

OH-POD-SMe **S4** (180 mg, 0.67 mmol, 1 equiv.) was weighed into four separate flasks so that each one contained ~45 mg. Each equivalent was dissolved in 10 mL acetone and cooled to 0°C. Then, a total of 100 µL Jones reagent was added to each flask in 10 µL aliquots over 3 h in 20 min intervals. After the last addition, the mixtures were stirred at room temperature for 10 min, then 20 mL dH_2O were added to each one. The flask contents were combined and extracted 3x with 80 mL EtOAc. The combined organic phases were washed with 80 mL brine, dried over $MgSO_4$ and concentrated *in vacuo*. The product COOH-POD-SMe **S5** was obtained as a white solid (168 mg, 0.60 mmol, 89% yield). ^{1}H NMR (500 MHz, DMSO-d_6): δ 12.41 (bs, 1H), 7.93–7.86 (m, 2H), 7.16–7.10 (m, 2H), 4.26 (t, *J* = 6.0 Hz, 2H), 2.75 (s, 3H), 2.73 (t, *J* = 6.0 Hz, 2H) ppm.

*COOH-PODS **3**.*

COOH-POD-SMe **S5** (166 mg, 0.59 mmol, 1 equiv.) was suspended in 5 mL EtOH and ammonium molybdate tetrahydrate (146 mg, 0.12 mmol, 0.2 equiv.) was added. The mixture was cooled to 0°C and stirred for 5 min, then H_2O_2 50% solution (101 µL, 1.78 mmol, 3 equiv.) was added. The mixture was warmed to room temperature and stirred for 1.5 h, then another 3 equiv. H_2O_2 were added. The mixture was stirred for 1 h, then for a third time 3 equiv.. H_2O_2 were added (total amount of H_2O_2: 303 µL in three aliquots). The mixture was stirred for 20 h, 45 mL dH_2O were added and the product was extracted 3x with 50 mL EtOAc. The combined organic phases were washed with 50 mL brine, dried over $MgSO_4$ and concentrated *in vacuo* to give a crude white solid, which was purified *via* silica column chromatography using a gradient of 1–10% MeOH in dichloromethane as the eluent. After solvent evaporation, the product COOH-PODS **7** was obtained as a white solid (122 mg, 0.39 mmol, 66% yield). ^{1}H NMR (500 MHz, DMSO-d_6): δ 12.45 (bs, 1H), 8.03 (d, *J* = 8.9 Hz, 2H), 7.20 (d, *J* = 8.9 Hz, 2H), 4.29 (t, *J* = 6.0 Hz, 2H), 3.70 (s, 3H), 2.75 (t, *J* = 6.0 Hz, 2H) ppm. HRMS (ESI-Q-Orbitrap)

pos. mode m/z: [2M + Na]+ calcd for $C_{12}H_{12}N_2O_6S$ 647.0724; found 647.0722; neg. mode m/z: [2M – H]− calcd for $C_{12}H_{12}N_2O_6S$ 623.0759; found 623.0757.

*DFO*PODS **6**.*

COOH-PODS **3** (11.7 mg, 0.034 mmol, 1.2 equiv.) and 3-oxo-1*H*-3λ^5-[1,2,3]triazolo[4,5-b]pyridine-1-carboximidamidium hexafluorophosphate (HATU) (10.9 mg, 0.028 mmol, 1.0 equiv.) were dissolved in 3 mL DMF in a microwave reactor vial equipped with a stirring bar. *N*,*N*-Diisopropylethylamine (DIPEA) (14.5 µL, 0.084 mmol, 3.0 equiv.) was added, and the mixture was stirred for 20 min at room temperature. [DFO*NH_2]TFA (25 mg, 0.028 mmol, 1 equiv.) and 1 mL DMF were added to the mixture, and the reaction was heated for 2 h at 50°C in a microwave reactor. RP-HPLC-MS confirmed that no starting material was left. Solvents were evaporated under high vacuum pressure. The residue was washed once with 7 mL ice-cold acetone by sonication, centrifugation (4700 g for 15 min at 4°C) and discarding the supernatant. The same washing procedure was repeated with 4 mL ice-cold MTBE. For purification via preparative HPLC, the crude product (30 mg) was dissolved in 5 mL DMSO, followed by the addition of 45 mL 26%-B. After purification (17 ml/min, A= mobile phase A=H_2O+0.1% TFA, mobile phase B=ACN+0.1% TFA, 28% B isocratic, 13.9 min product peak) the product DFO*PODS **6** was obtained as off-white solid after lyophilization (9% yield, 98% (HPLC)). The final product was characterized by RP-HPLC-MS, HR-MS, ^{1}H-, ^{13}C-, COSY-, HSQC- and HMBC-NMR.

RP-HPLC-MS: Gradient = 0.5-6.5 min 5-95% B, UV (λ=220nm) product peak at 4.08 min, m/z: [M+H]+ calcd for $C_{46}H_{74}N_{10}O_{16}S$ 1055.5; found 1055.7.

HR-MS (ESI-Q-Orbitrap) m/z: [M+H]+ calcd for $C_{46}H_{74}N_{10}O_{16}S$ 1055.5078; found 1055.5094.

^{1}H-NMR (500.10 MHz, DMSO) δ 9.74 – 9.50 (m, 4H), 8.03 (d, *J* = 8.7 Hz, 2H), 7.99 (m, 1H), 7.78 (m, 3H), 7.18 (d, *J* = 8.8 Hz, 2H), 4.29 (m, 2H), 3.70 (s, 3H), 3.46 – 3.43 (m, 8H), 3.07 – 3.04 (m, 2H), 2.99 (m, 6H), 2.58 – 2.55 (m, 8H), 2.26 (m, 6H), 1.96 (s, 3H), 1.53 – 1.46 (m, 8H), 1.38 (m, 7.4 Hz, 8H), 1.22 (m, 8H).

^{13}C-NMR (150.93 MHz, DMSO-d6) δ 171.97, 171.30, 170.13, 169.08, 165.77, 162.23, 161.67, 129.44, 115.52, 114.24, 64.67, 47.08, 46.78, 42.91, 38.43, 35.08, 29.88, 29.04, 28.83, 28.77, 27.57, 26.04, 23.50, 20.37.

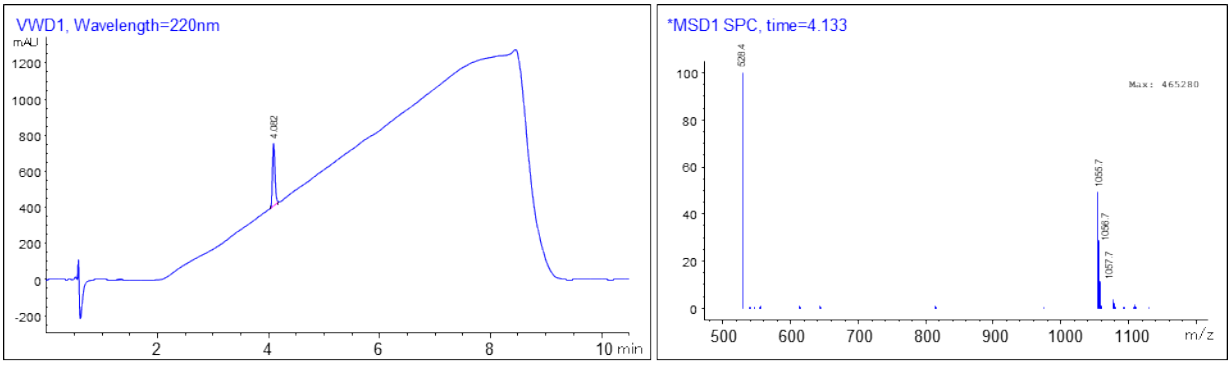

Figure S 7 RP-HPLC-MS chromatogram of DFO*PODS **6**. Gradient: 0.5-6.5 min 5-95% B. Left: UV-chromatogram (λ=220nm). Right: MS spectrum of product peak.

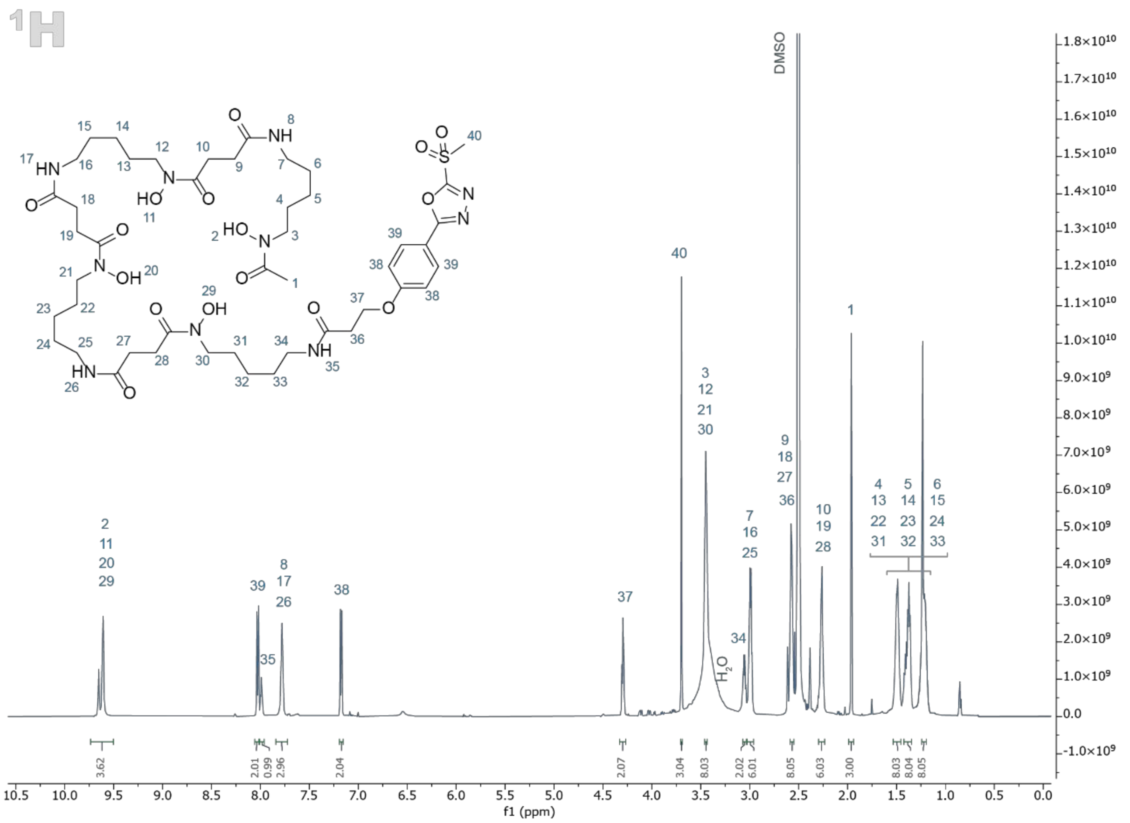

Figure S 8 ^{1}H-NMR (600.18 MHz, DMSO-d6) spectrum of DFO*PODS **6**. The signals at 1.23 and 0.85 ppm occurred after the compound was dissolved in DMSO while heating to 40°C (due to low solubility) in an 1.5 mL plastic tube. The signals also occurred when only heating DMSO in another tube in the same conditions. They probably result from plasticizer extracted from the tube.

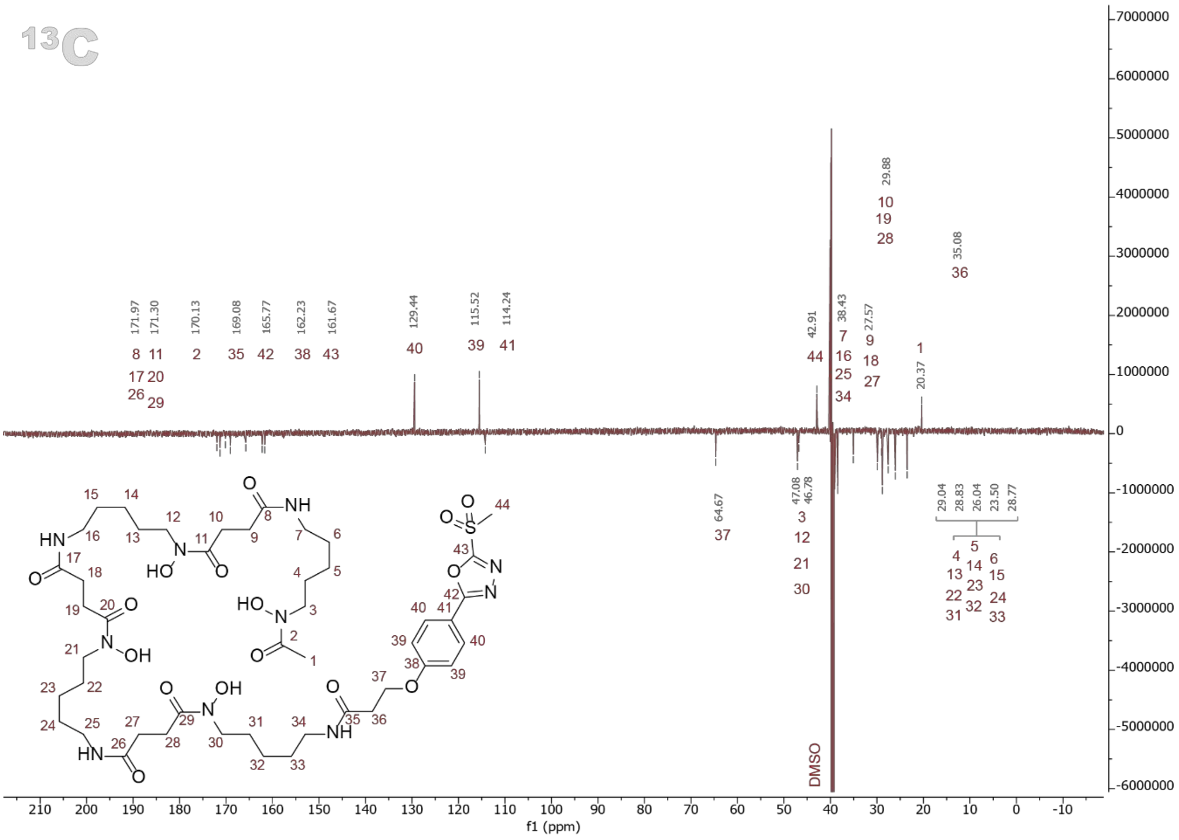

Figure S 9 ^{13}C-NMR (150.93 MHz, DMSO-d6) spectrum of DFO*PODS **6**.

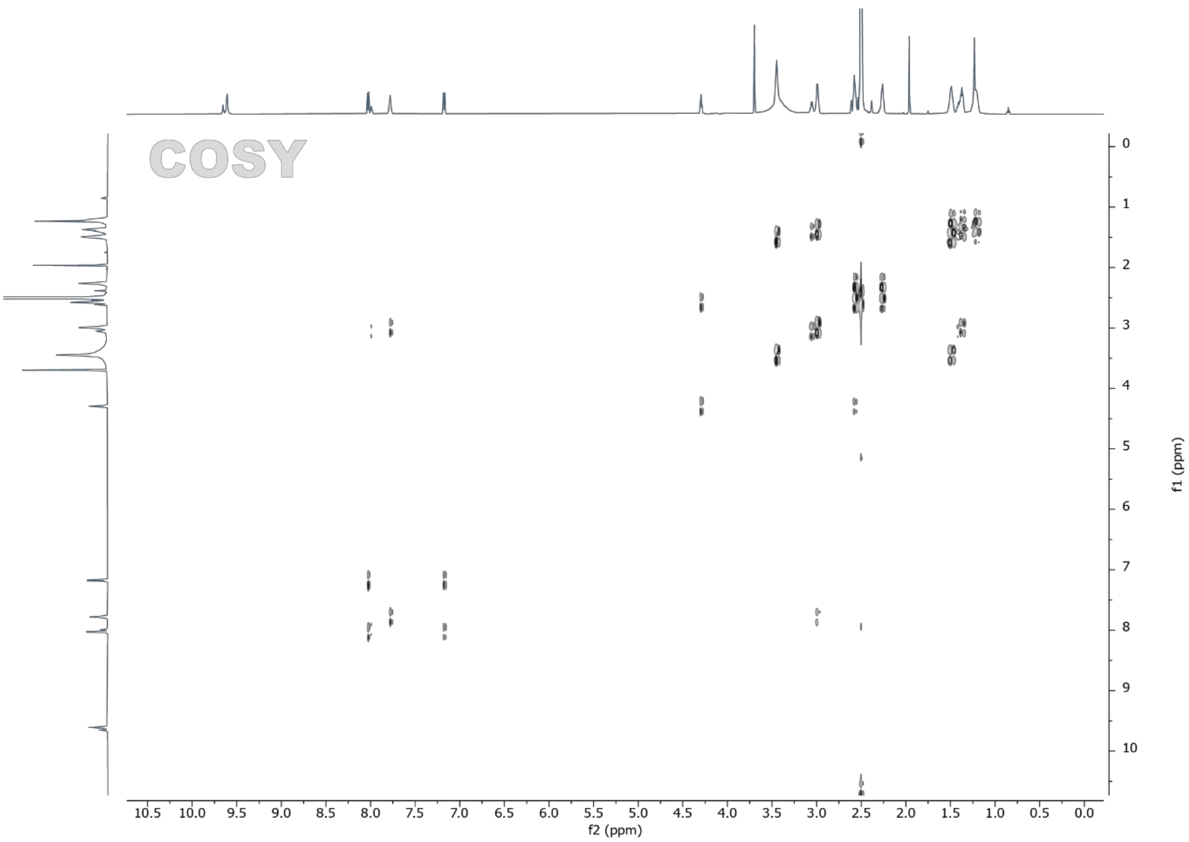

Figure S 10 COSY-NMR (DMSO-d6) spectrum of DFO*PODS **6**.

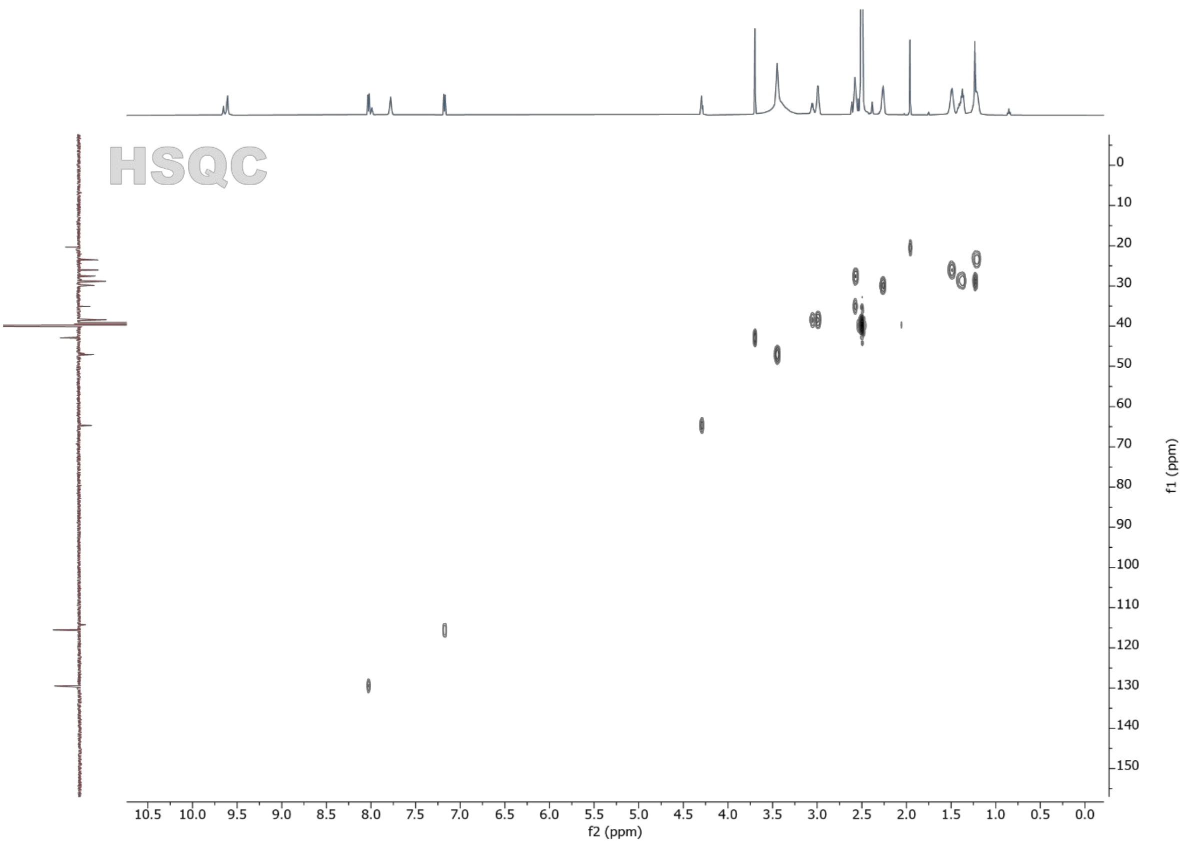

Figure S 11 HSQC-NMR (DMSO-d6) spectrum of DFO*PODS **6**.

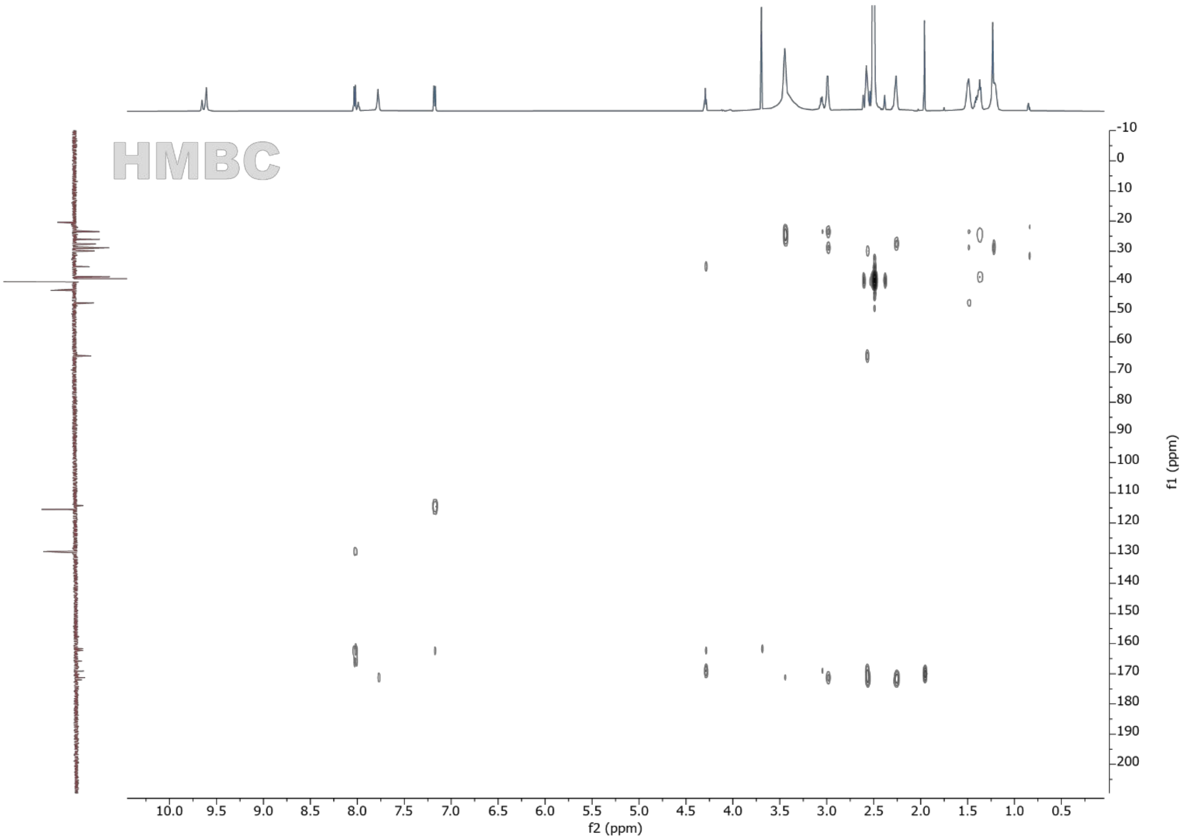

Figure S 12 HMBC-NMR (DMSO-d6) spectrum of DFO*PODS **6**.

*COOtBu-VK **S6**.*

3-Benzoylacrylic acid (220 mg, 1.21 mmol, 1 equiv.) was dissolved in 2.2 mL dry dimethoxyethane and *N*-methylmorpholine (136 µL, 1.21 mmol, 1 equiv.) was added. The solution was cooled to 0°C and stirred for 5 min, then isobutyl chloroformate (193 µL, 1.45 mmol, 1.2 equiv.) was added and stirring was continued for another 5 min. A solution of *tert*-butyl 3-aminopropanoate hydrochloride (232 mg, 1.21 mmol, 1 equiv.) and *N*-methylmorpholine (136 µL, 1.21 mmol, 1 equiv.) in 2.2 mL dry DMF was added and the mixture stirred for 10 min in the cold. Then, the mixture was warmed to room temperature, stirred for 30 min and 10 mL dH_2O were added. The pH was adjusted to 1 using 1 M HCl and the aqueous phase subsequently extracted 3x with 20 mL DCM. The combined organic phases were dried over Na_2SO_4 and concentrated *in vacuo*. The product COOtBu-VK **S6** was obtained as an orange solid (377 mg, 1.21 mmol, >99% yield). ^{1}H NMR (500 MHz, DMSO-d_6): δ 8.68 (t, *J* = 4.9 Hz, 1H), 8.01 (d, *J* = 7.7 Hz, 2H), 7.75 (d, *J* = 15.3 Hz, 1H), 7.70 (t, *J* = 7.2 Hz, 1H), 7.58 (t, *J* = 7.5 Hz, 2H), 6.98 (d, *J* = 15.4 Hz, 1H), 3.38 (q, *J* = 6.3 Hz, 2H), 2.44 (t, *J* = 6.6 Hz, 2H), 1.40 (s, 9H) ppm.

*COOH-VK **4**.*

COOtBu-VK **S6** (50 mg, 0.16 mmol, 1 equiv.) was dissolved in 2 mL DCM and TFA (641 µL, 8.24 mmol, 50 equiv.) was added. The mixture was stirred at room temperature for 1 h, then all volatiles were removed *in vacuo*. The residue was triturated in DCM, filtered and the filter washed 2x with DCM. The product COOH-VK **4** was obtained as a white solid (30 mg, 0.12 mmol, 74% yield). ^{1}H NMR (500 MHz, DMSO-d_6): δ 12.31 (bs, 1H), 8.78–8.59 (m, 1H), 8.01 (d, *J* = 7.5 Hz, 2H), 7.74 (d, *J* = 15.4 Hz, 1H), 7.70 (t, *J* = 7.3 Hz, 1H), 7.58 (t, *J* = 7.5 Hz, 2H), 6.98 (d, *J* = 15.3 Hz, 1H), 3.42–3.36 (m, 2H, overlap with water signal), 2.45 (t, *J* = 6.6 Hz, 2H) ppm. HRMS (ESI-Q-Orbitrap) pos. mode m/z: $[M + Na]^+$ calcd for $C_{13}H_{13}NO_4$ 270.0737; found 270.0737; neg. mode m/z: $[M – H]^–$ calcd for $C_{13}H_{13}NO_4$ 246.0772; found 246.0771.

*DFO*VK* **7**.

COOH-VK **4** (8.1 mg, 0.033 mmol, 1.2 equiv.) and HATU (10.4 mg, 0.027 mmol, 1 equiv.) were dissolved in 3 mL DMF in a microwave reactor vial equipped with a stirring bar. DIPEA (14 µL, 0.082 mmol, 3 equiv.) was added and the mixture was stirred for 20 min at room temperature. [DFO*NH_2]TFA (24 mg, 0.027 mmol, 1 equiv.) and 1.5 mL DMF were added to the mixture, and the reaction was heated for 1 h at 50°C in a microwave reactor. RP-HPLC-MS confirmed that no starting material was left. Solvents were evaporated under high vacuum pressure. The residue was washed once with ice-cold acetone by sonication, centrifugation (4700 g for 15 min at 4°C) and discarding the supernatant. The same washing procedure was repeated with 4 mL ice-cold MTBE. For purification via preparative HPLC, the crude product (25 mg) was almost completely dissolved in 5 mL DMSO and 45 mL 25% ACN/H_2O/0.1%TFA. After purification (17 ml/min, A= mobile phase A=H_2O+0.1% TFA, mobile phase B=ACN+0.1% TFA, 25% B isocratic, 11.7 min product peak) the product DFO*-VK **7** was obtained as off-white solid after lyophilization (27% yield, 99% (HPLC)). The final product was characterized by RP-HPLC-MS, HR-MS, ^{1}H-, ^{13}C-, COSY-, HSQC- and HMBC-NMR.

RP-HPLC-MS: Gradient = 0.5-6.5 min 5-95% B, UV (λ=220nm) product peak at 4.09 , m/z $[M+H]^+$ calcd for $C_{47}H_{75}N_9O_{14}$ 990.6; found 990.8.

HR-MS (ESI-Q-Orbitrap) m/z: $[M+H]^+$ calcd for $C_{47}H_{75}N_9O_{14}$ 990.5506; found 990.5511.

^{1}H-NMR (700.40 MHz, DMSO-d6) δ 9.62 (m, 4H), 8.65 (t, *J* = 5.8 Hz, 1H), 8.02 – 7.98 (m, 2H), 7.86 (t, *J* = 5.6 Hz, 1H), 7.77 (t, *J* = 5.5 Hz, 3H), 7.73 (d, *J* = 15.3 Hz, 1H), 7.71 – 7.68 (m, 1H), 7.59 – 7.56 (m, 2H), 6.98 (d, *J* = 15.3 Hz, 1H), 3.45 (m, 9H), 3.05 – 2.98 (m, 8H), 2.61 – 2.53 (m, 6H), 2.30 (t, *J* = 7.0 Hz, 2H), 2.26 (t, *J* = 7.5 Hz, 5H), 1.96 (s, 3H), 1.49 (m, 8H), 1.38 (m, 8H), 1.21 (m, 8H).

^{13}C-NMR (176.13 MHz, DMSO-d6) δ 189.89, 171.96, 171.30, 169.85, 163.39, 136.61, 136.50, 133.73, 131.80, 129.01, 128.62, 47.07, 46.78, 38.42, 38.40, 35.74, 35.04, 29.88, 28.81, 28.77, 27.56, 26.02, 23.51, 23.49, 20.34.

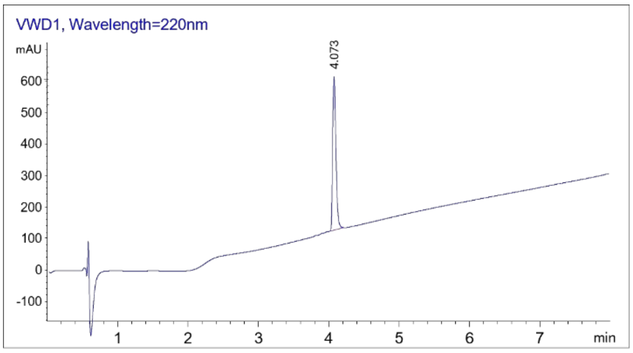

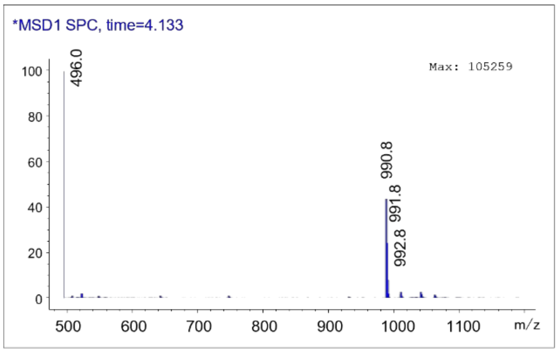

Figure S 13 RP-HPLC-MS chromatogram of DFO*-VK **7**. Gradient = 0.5-6.5 min 5-95% B. Left: UV-chromatogram (λ=220nm). Right: MS spectrum of product peak.

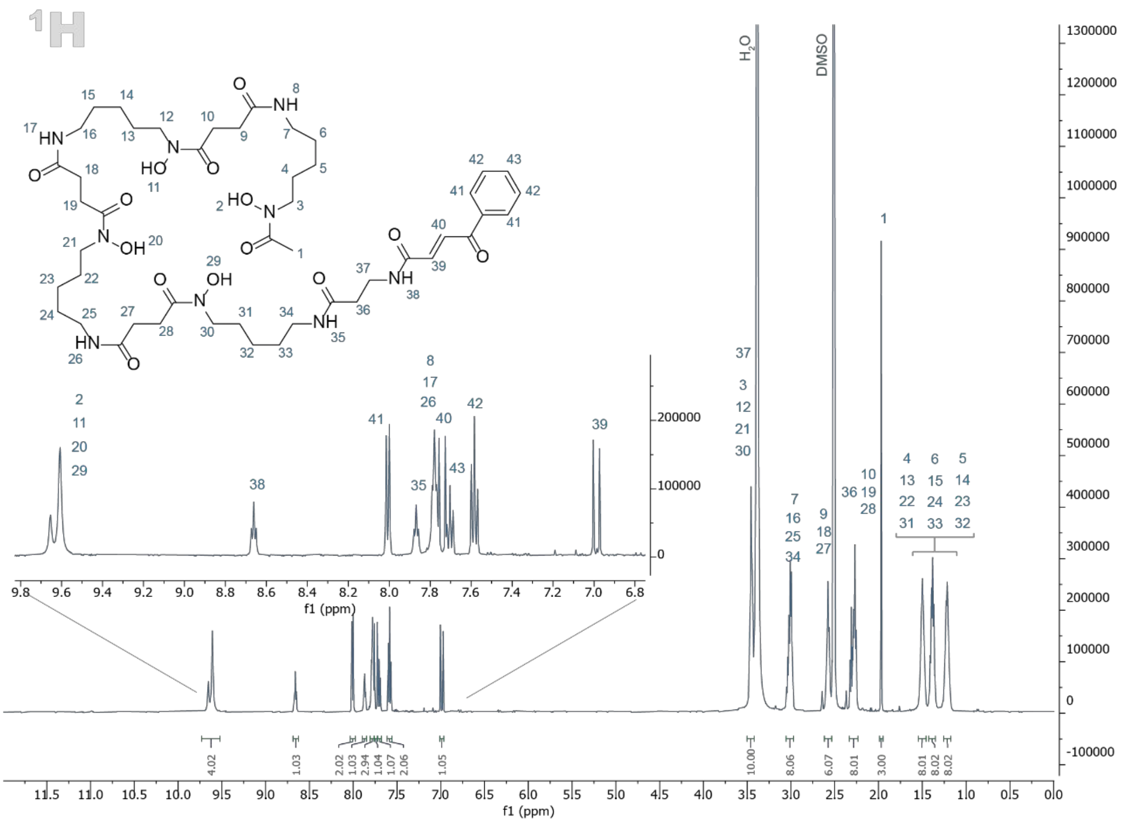

Figure S 14 ^{1}H-NMR (700.40 MHz, DMSO-d6) spectrum of DFO*VK **7**.

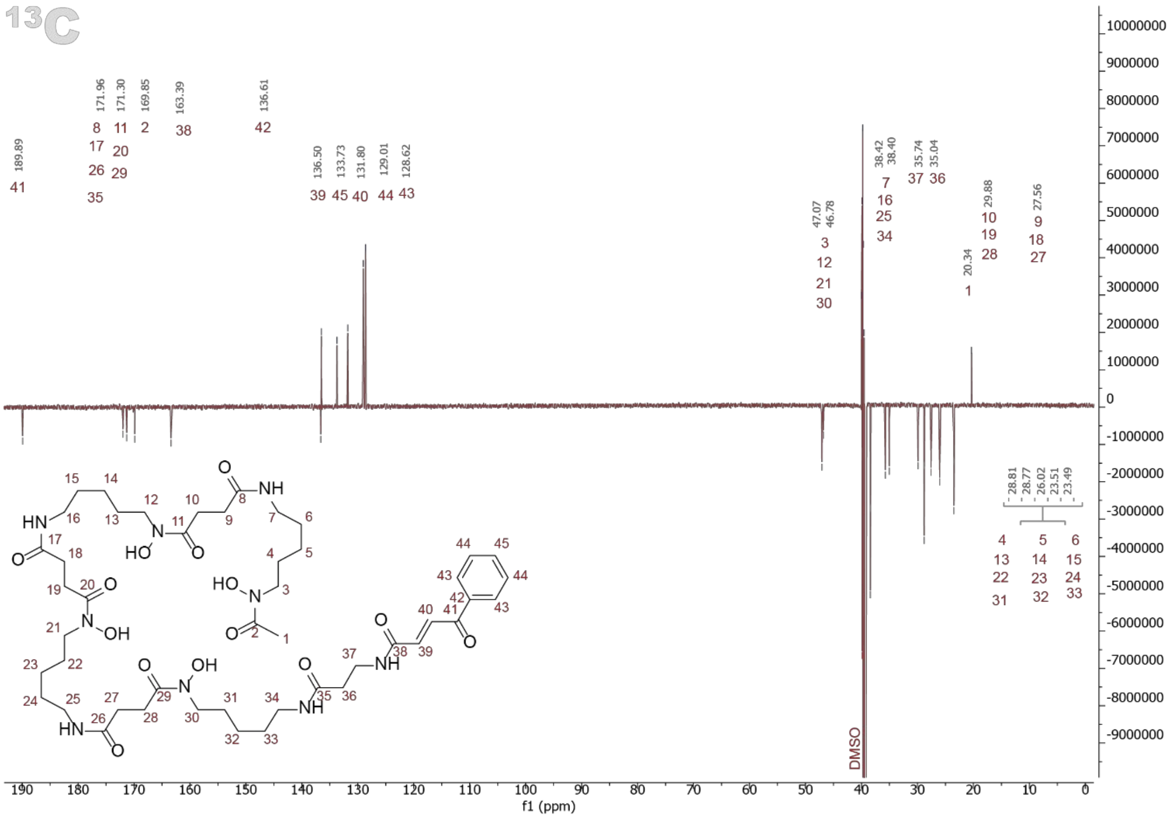

Figure S 15 ^{13}C-NMR (176.13 MHz, DMSO-d6) spectrum of DFO*VK **7**.

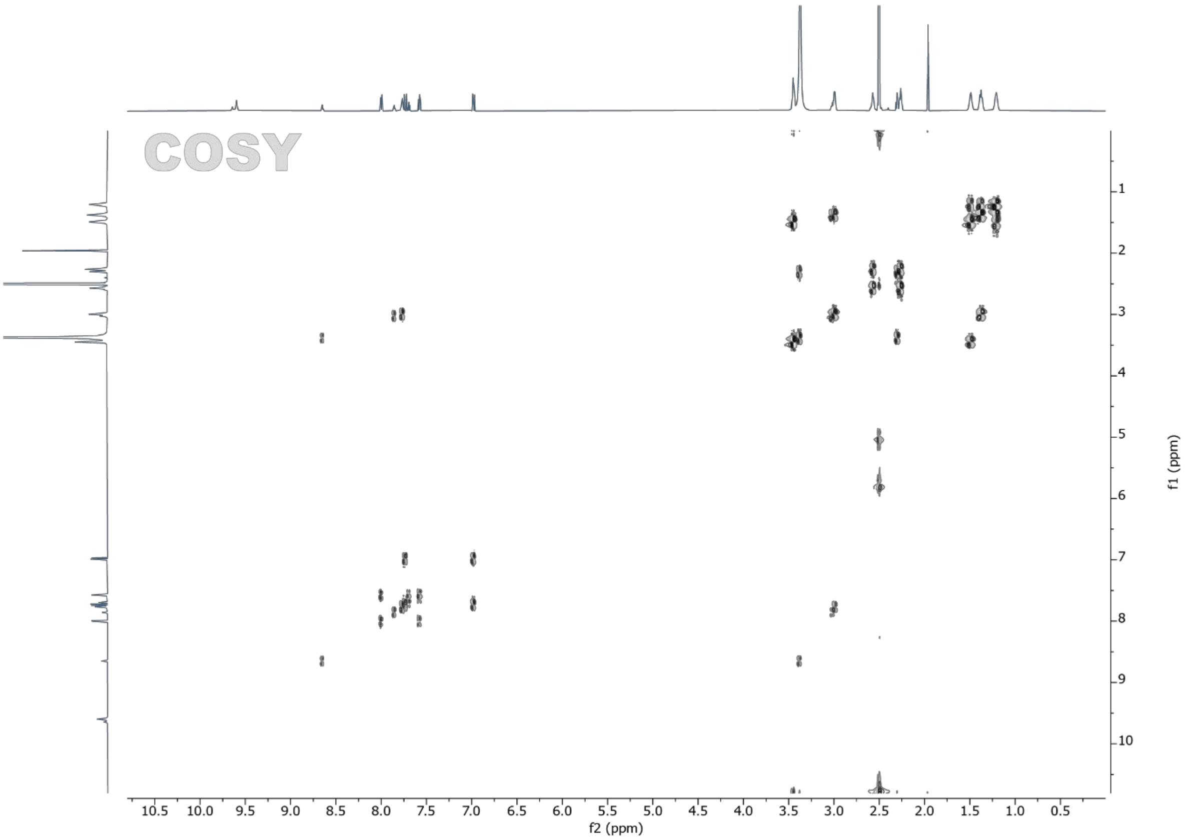

Figure S 16 COSY-NMR (DMSO-d6) spectrum of DFO*VK **7**.

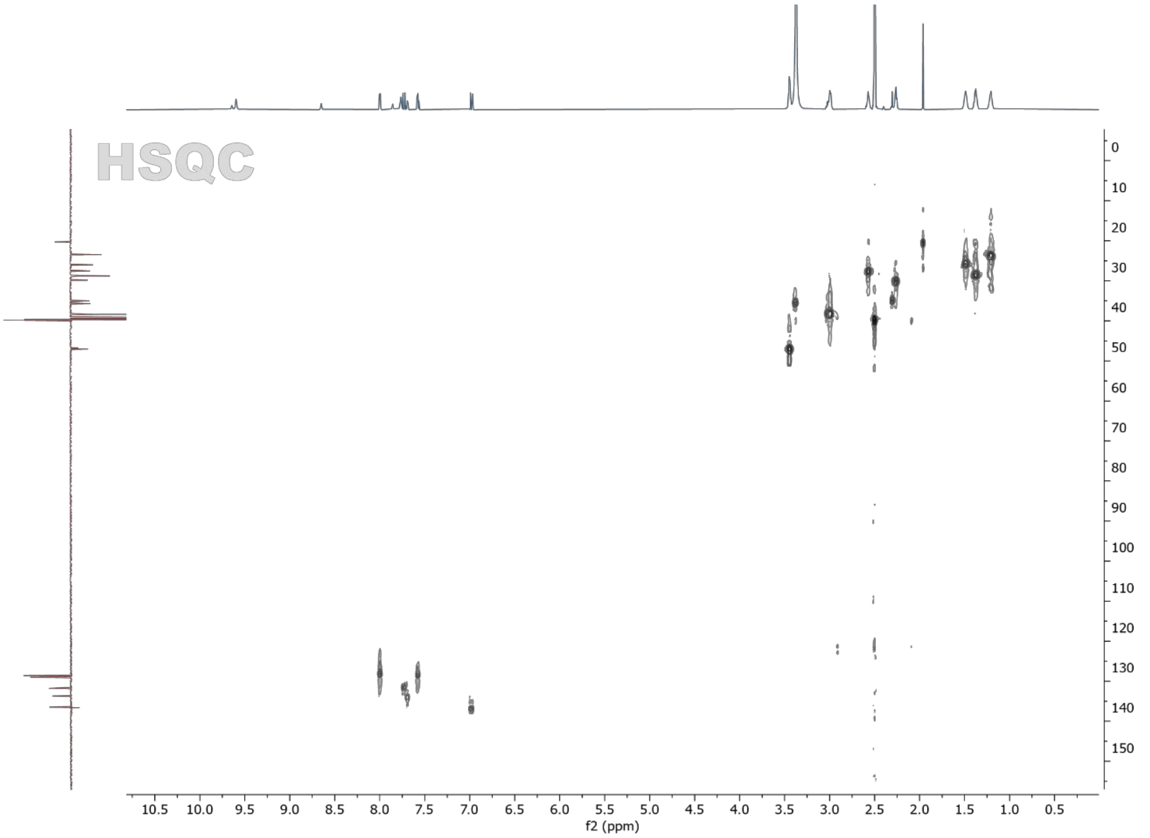

Figure S 17 HSQC-NMR (DMSO-d6) spectrum of DFO*VK **7**.

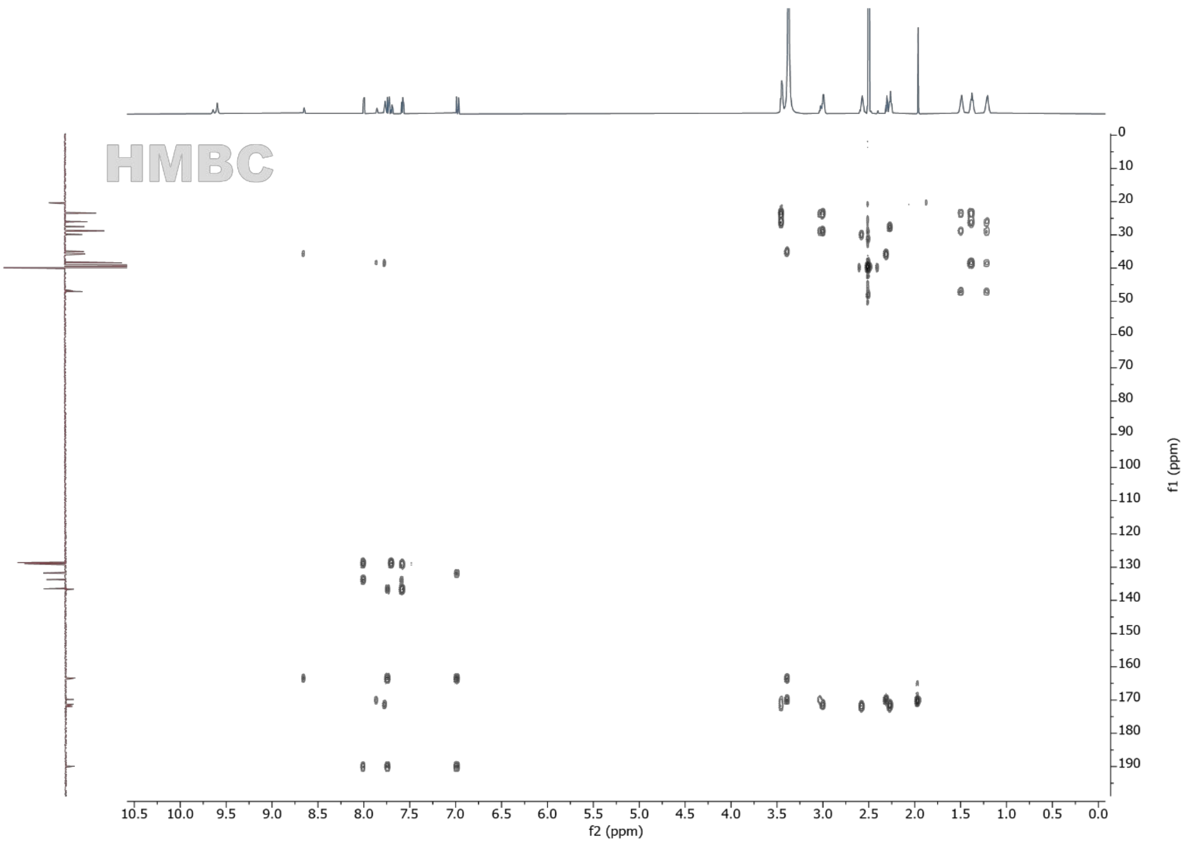

Figure S 18 HMBC-NMR (DMSO-d6) spectrum of DFO*VK **7**.

*Side-product DFO*shortVK* ***S7****.*

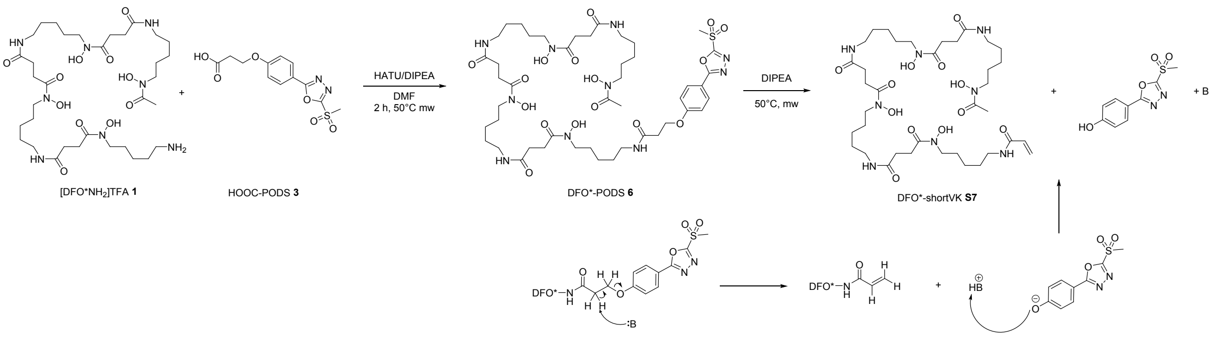

Scheme S 2 Mechanism hypothesized for the formation of the side product DFO*shortVK **S7**.

During the synthesis of **6** a prominent side product formed, indicated first by LCMS. It could be isolated in the same preparative HPLC purification runs as **6** (retention time = 6.1 min, (12% yield, 98% (HPLC)). The white solid was analyzed via RP-HPLC-MS, HR-MS, ^{1}H-, ^{13}C-, COSY-, HSQC- and HMBC-NMR and thereby identified as DFO*shortVK **S7**. We hypothesize that the PODS-linker acts as leaving group in a β-elimination enabled by the presence of DIPEA. The side-product DFO*shortVK **VII** was incubated with HSA likewise to the other BFCAs, but isotopic dilution assays revealed chelator-to-protein ratios <0.02. Therefore, this compound was not further investigated in this study.

RP-HPLC-MS: Gradient = 0.5-6.5 min 5-95% B, UV (λ=220nm) product peak at 3.55 min, m/z $[M+H]^+$ calcd for $C_{37}H_{66}N_8O_{12}$ 815.5; found 815.7.

HR-MS (ESI-TOF) m/z: $[M+H]^+$ calcd for $C_{37}H_{66}N_8O_{12}$ 815.4878; found 815.4879.

^{1}H-NMR (600.18 MHz, DMSO) δ 9.62 (m, 4H), 8.04 (s, 1H), 7.77 (m, 3H), 6.22 – 6.18 (dd, J = 17.1, 10.1 Hz, 1H), 6.07 – 6.04 (dd, J = 17.1, 2.2 Hz, 1H), 5.55 (dd, *J* = 10.1, 2.2 Hz, 1H), 3.48 – 3.43 (m, 8H), 3.10 (q, *J* = 6.7 Hz, 2H), 3.00 (m, 6H), 2.57 (m, 6H), 2.27 (m, 6H), 1.96 (s, 3H), 1.50 (m, 8H), 1.39 (m, 8H), 1.26 – 1.19 (m, 8H).

^{13}C-NMR (150.93 MHz, DMSO) δ 171.95, 171.27, 170.06, 164.42, 131.85, 124.75, 47.06, 46.76, 38.42, 38.40, 29.86, 28.80, 28.70, 27.53, 26.00, 23.51, 23.47, 20.33.

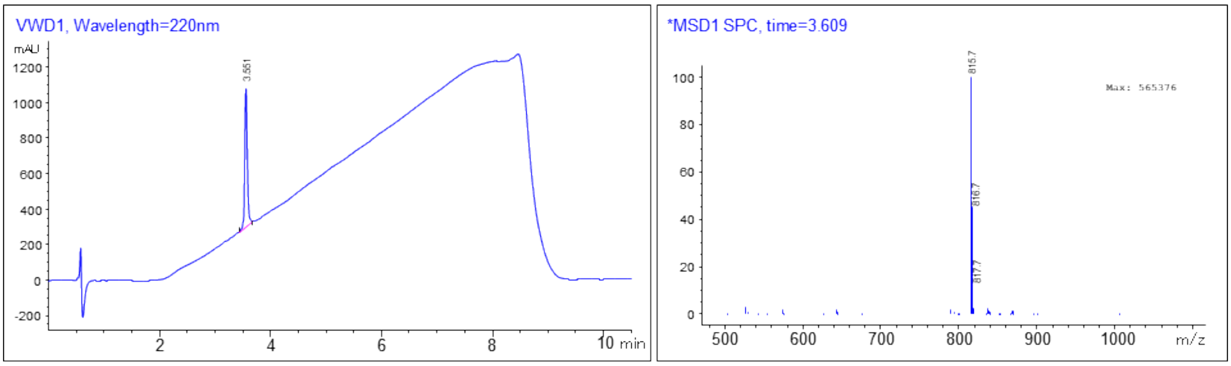

Figure S 19 RP-HPLC-MS chromatogram of DFO*shortVK **S7**. Gradient = 0.5-6.5 min 5-95% B. Left: UV-chromatogram (λ=220nm). Right: MS spectrum of product peak.

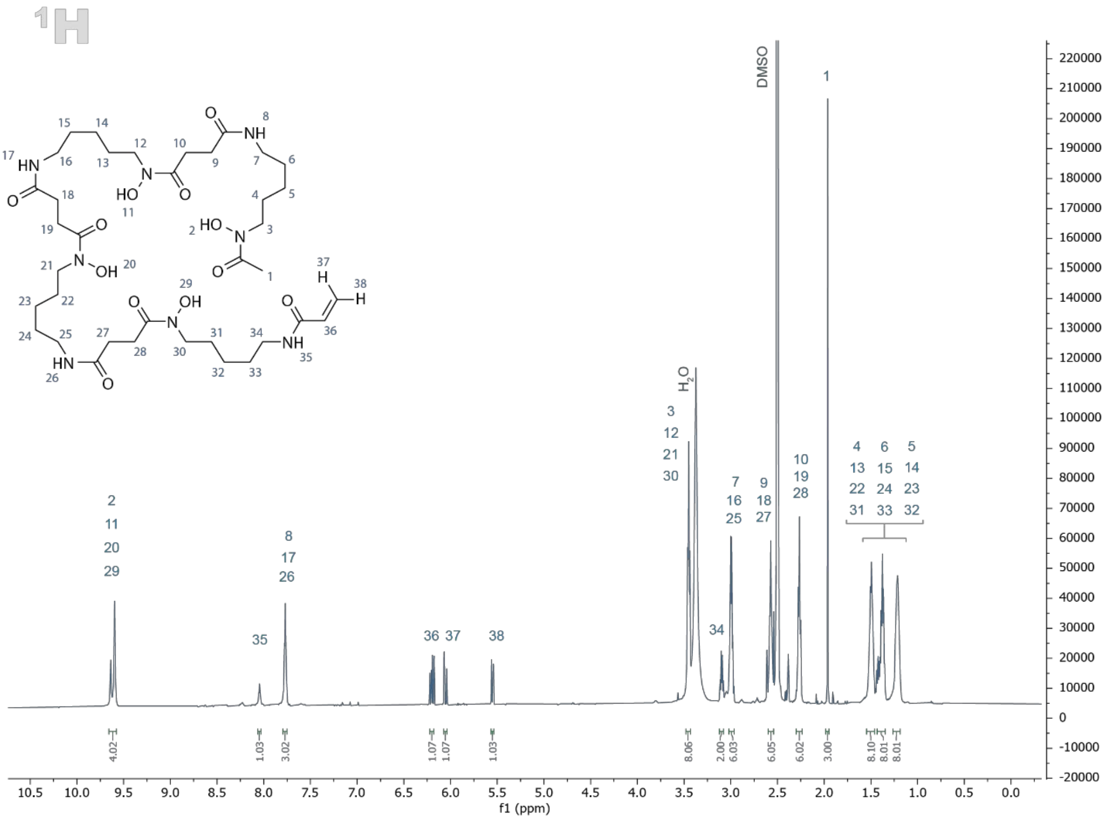

Figure S 20 ^{1}H-NMR (600.18 MHz, DMSO) spectrum of DFO*shortVK **S7**.

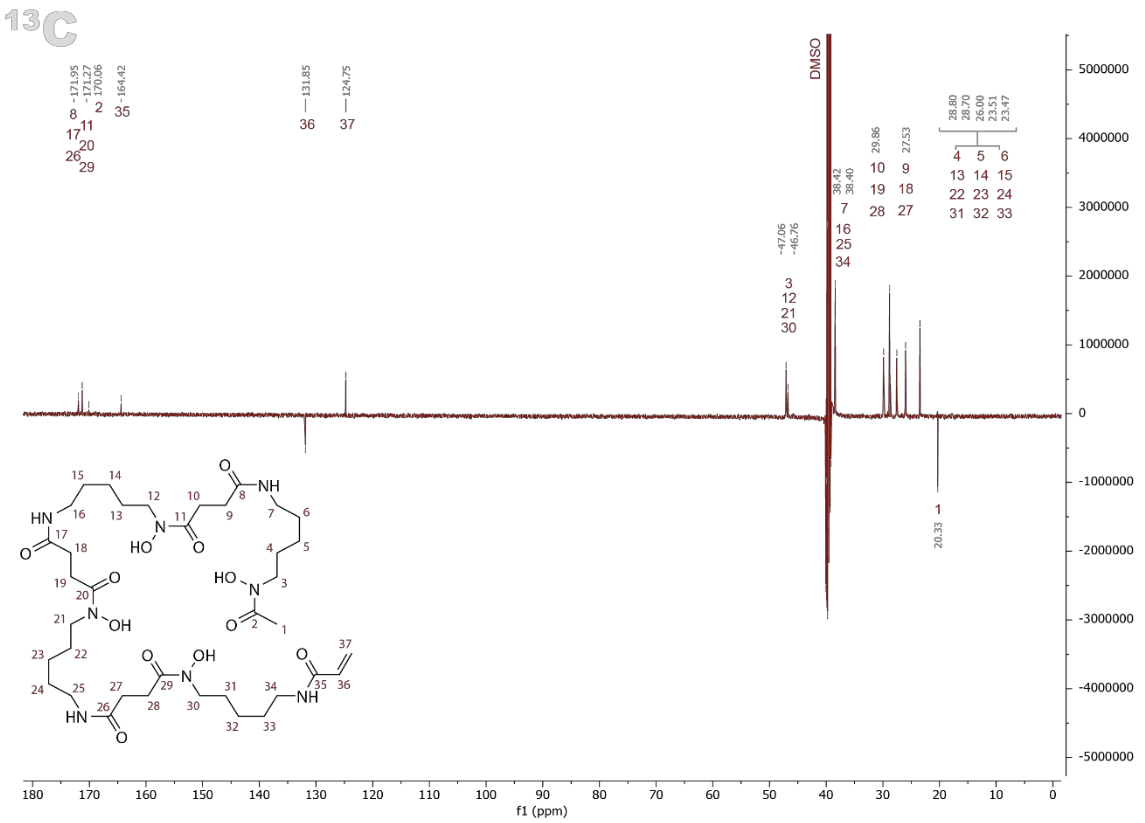

Figure S 21 ^{13}C-NMR (150.93 MHz, DMSO) spectrum of DFO*shortVK **S7**.

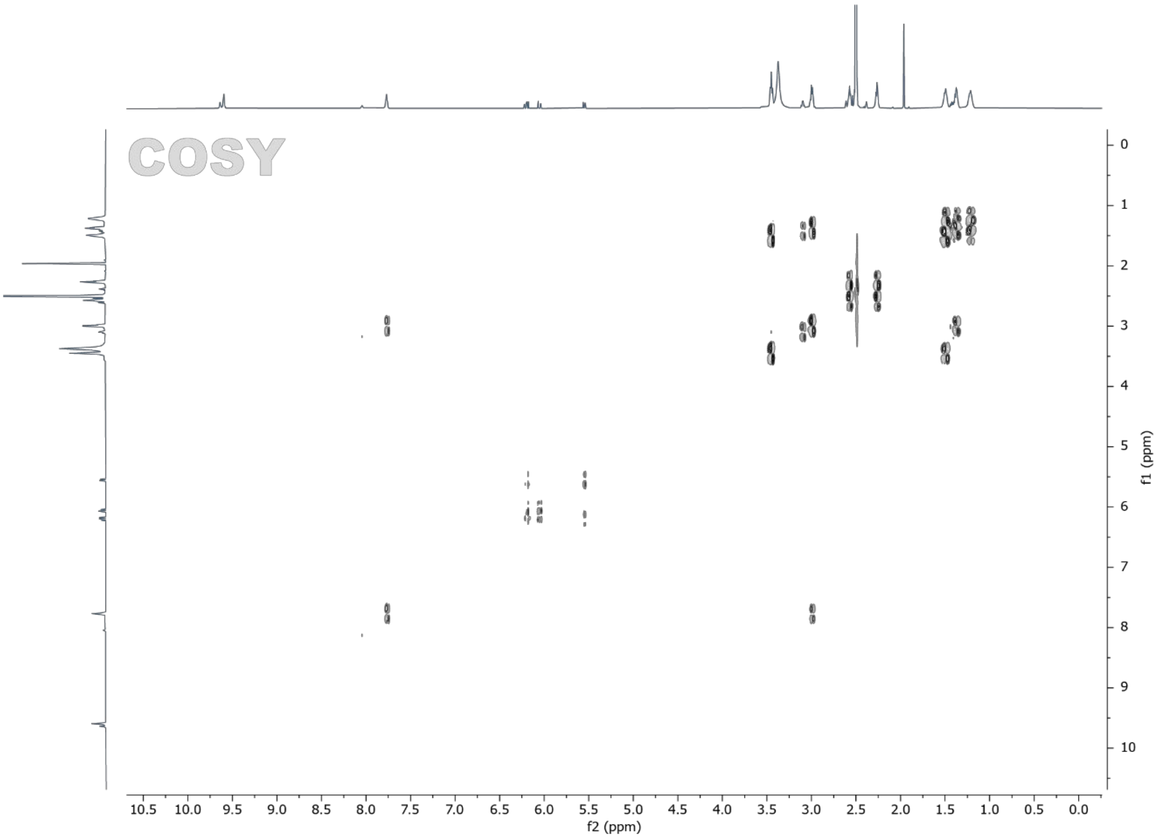

Figure S 22 COSY-NMR (DMSO-d6) spectrum of DFO*shortVK **S7**.

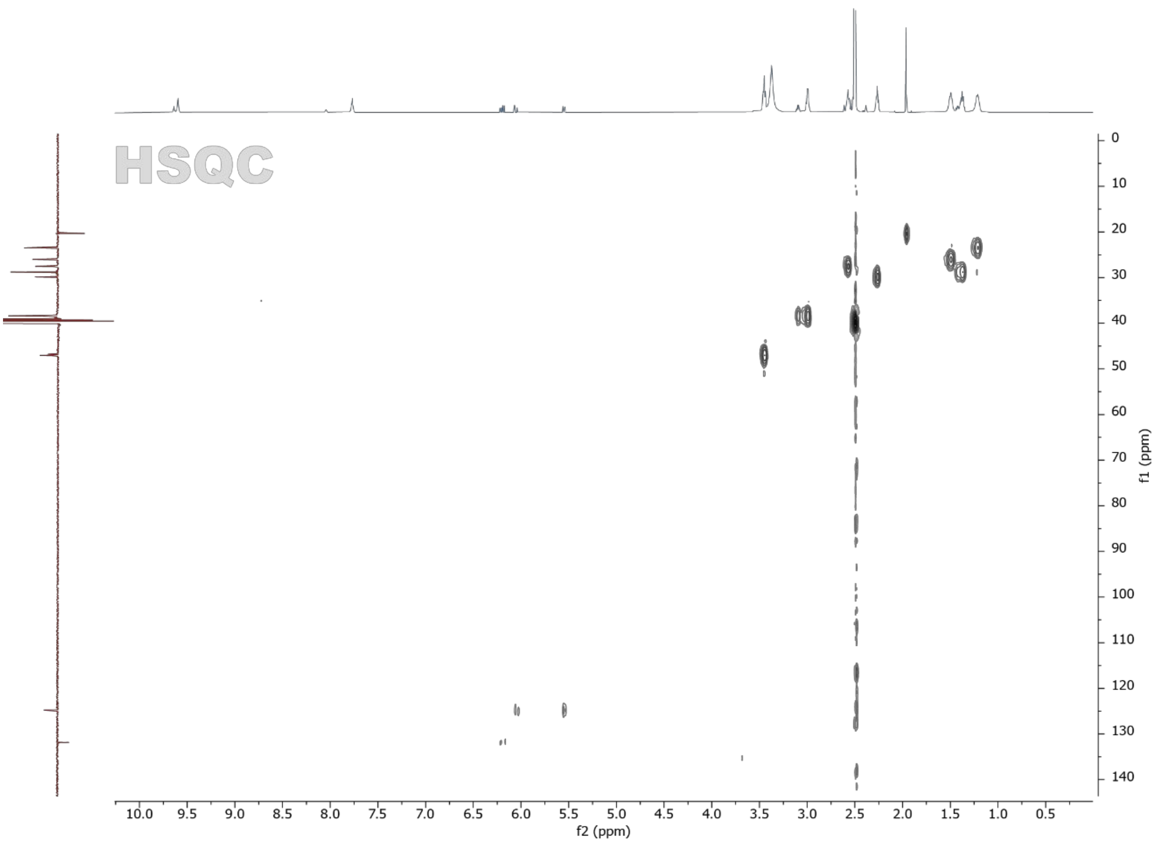

Figure S 23 HSQC-NMR (DMSO-d6) spectrum of DFO*shortVK **S7**.

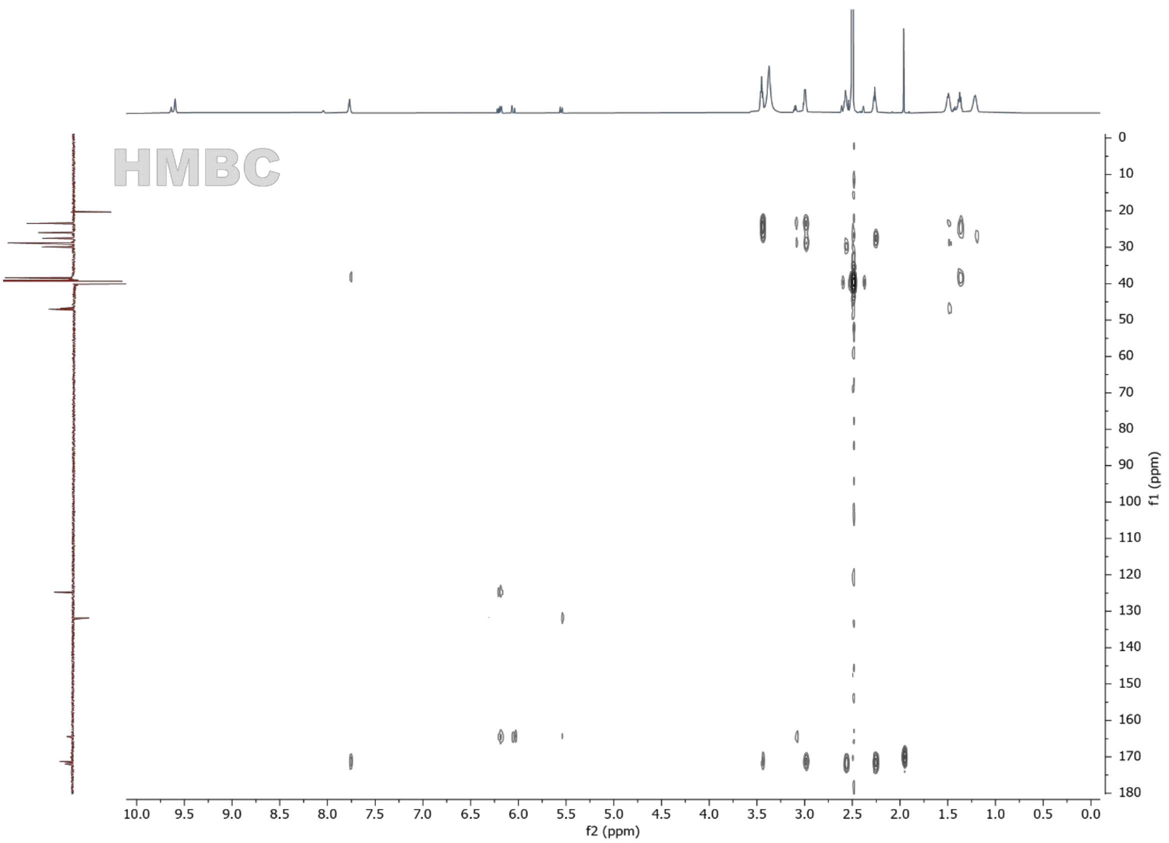

Figure S 24 HMBC-NMR (DMSO-d6) spectrum of DFO*shortVK **S7**.

Ac-QQCPF-NH2 **14.**

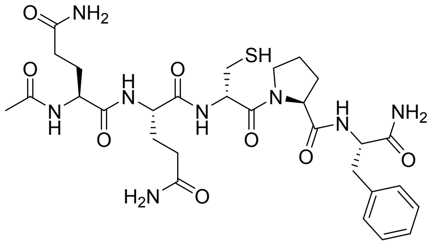

Chemical Formula: $C_{29}H_{42}N_8O_8S$
Molecular Weight: 662.76

The synthesis was performed automatically with the microwave-assisted peptide synthesizer Biotage® Initiator + Alstra™ by standard Fmoc/tBu chemistry. The reaction was carried out in a 10 mL vial at a scale of 0.075 mmol. The Rink amide MBHA (4-methylbenzylhydrylamine) resin (0.065 mmol/g loading, 100–200 mesh) was used as solid support. The resin was swollen in DMF at 70°C for 20 min. The cleavage of the Fmoc-protecting group was accomplished using 2 × 5 ml 20% piperidine in DMF at room temperature for 3 and 10 min reaction time, respectively. After the Fmoc-deprotection, the resin was washed with DMF (2 × 5 mL). The amino acids (5 equiv.) were prepared in 0.5 M solutions in DMF. HATU (5 equiv., 0.5 M in DMF) and DIPEA (10 equiv., 0.5 M in DMF) were used as coupling reagents. The first amino acid, Fmoc-Phe-OH, was coupled two times for 5 min at 75°C, followed by cleavage of the Fmoc group as described before. The second amino acid, Fmoc-Pro-OH, was coupled and cleaved the same way. Fmoc-Cys(Trt)-OH was coupled two times at room temperature for 60 min each, followed by Fmoc cleavage. Fmoc-Gln(Trt)-OH) was coupled for 5 min at 75°C, followed by cleavage of the Fmoc group, and repeated once more. Afterwards, the amine was capped using 67 equiv. of acetic anhydride (5 M in DMF) for 10 min at room temperature.
After the completion of the sequence, a washing step of the resin was carried out with DCM (3 × 3 mL). The deprotection of the peptides and cleavage from the resin was performed using 4 mL TFA/H_2O/TIPS/thioanisole/ethane-1,2-dithiol (82.5:5:5:5:2.5) for 3 h at room temperature on a shaker. The cleaved peptide was dried under a stream of argon, precipitated with ice-cold MTBE (3 mL), and centrifuged. After two additional washes with ice-cold MTBE followed by vortexing and sonication, and centrifugation, the crude product was isolated and analyzed by RP-HPLC-MS.
After purification via preparative HPLC (17 mL/min, A= mobile phase A=H_2O+0.1% TFA, mobile phase B=ACN+0.1% TFA, 0-3 min 17%B, -13 min 19%B, 13.1 - 18.1 min 95%B, 10.9 min product peak) the product Ac-QCCPF **14** was obtained as white solid after lyophilization (56% yield, 27.9 mg, 98% (HPLC)). The final product was characterized by RP-HPLC-MS and HR-MS.

RP-HPLC-MS: Gradient = 0.5-6.0 min 5-95% B, UV (λ=220nm) product peak at 3.52 min, m/z $[M+H]^+$ calcd for $C_{29}H_{42}N_8O_8S$ 663.3; found 663.2.

HR-MS (ESI-Q-Orbitrap) m/z: $[M+H]^+$ calcd for $C_{29}H_{42}N_8O_8S$ 663.2919; found 663.2902, $[M+Na]^+$ cacld for $C_{29}H_{41}N_8O_8SNa$ 685.2718; found 685.2739.

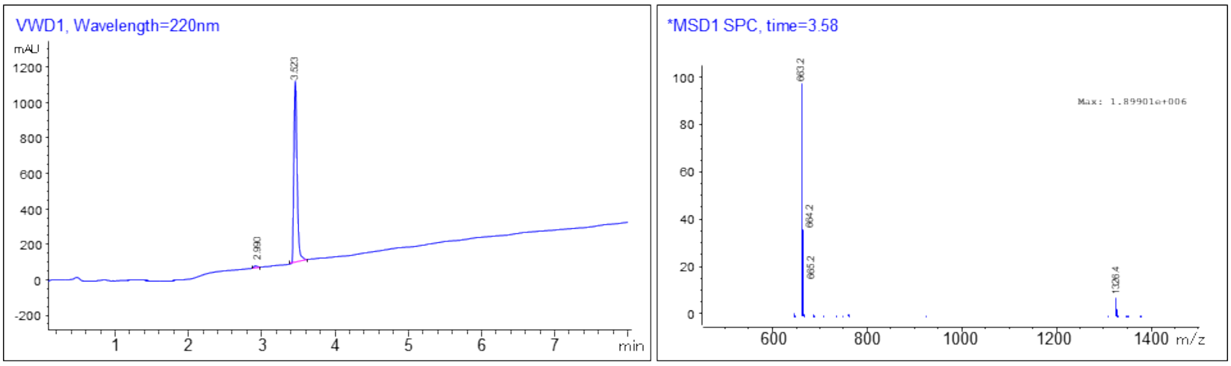

Figure S 25 RP-HPLC-MS chromatogram of Ac-QQCPF **14**. Gradient = 0.5-6.5 min 5-95% B. Left: UV-chromatogram (λ=220nm). Right: MS spectrum of product peak.

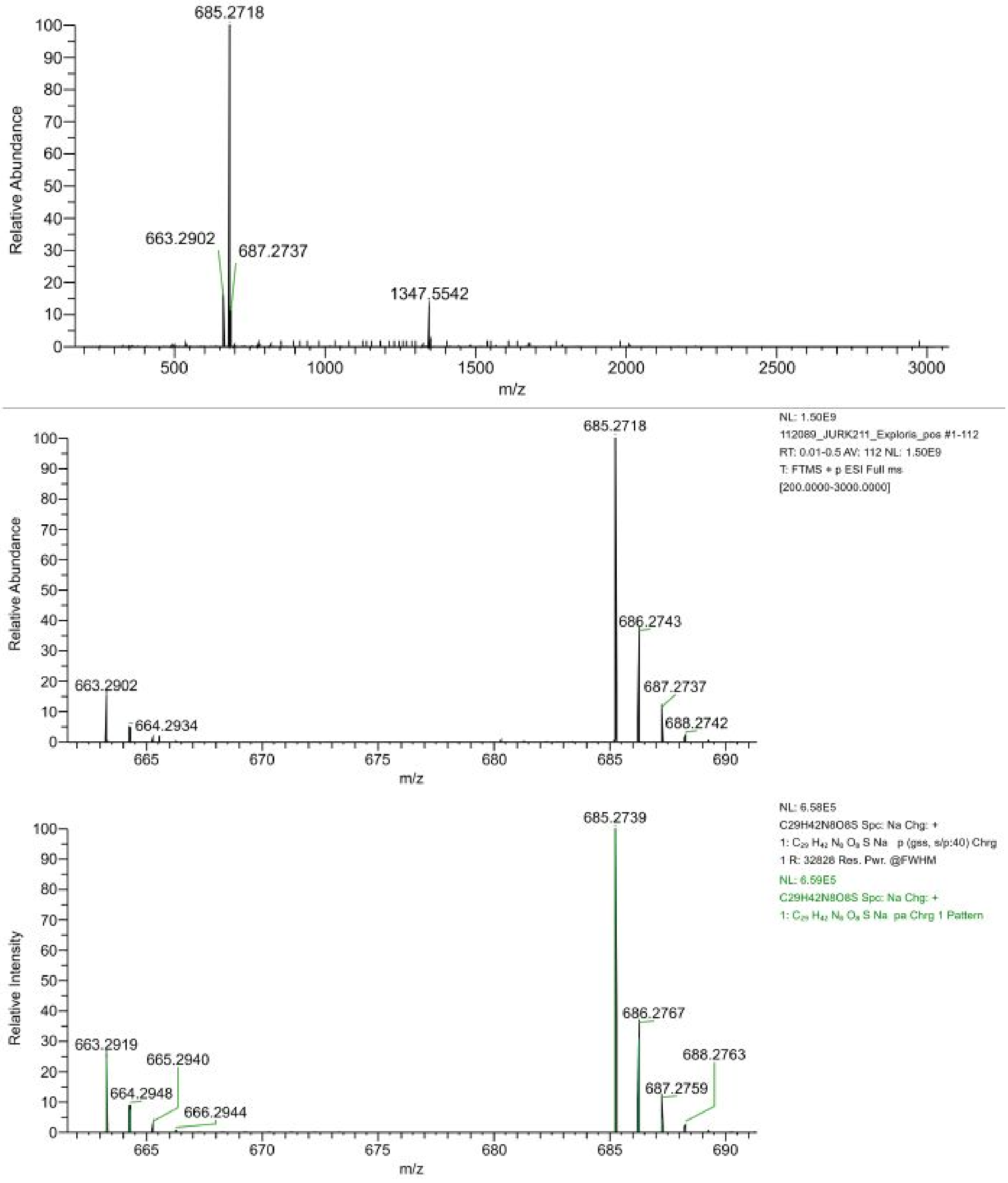

Figure S 26. HR-MS (ESI-TOF) spectrum of Ac-QCCPF **14**.

V. Kinetics

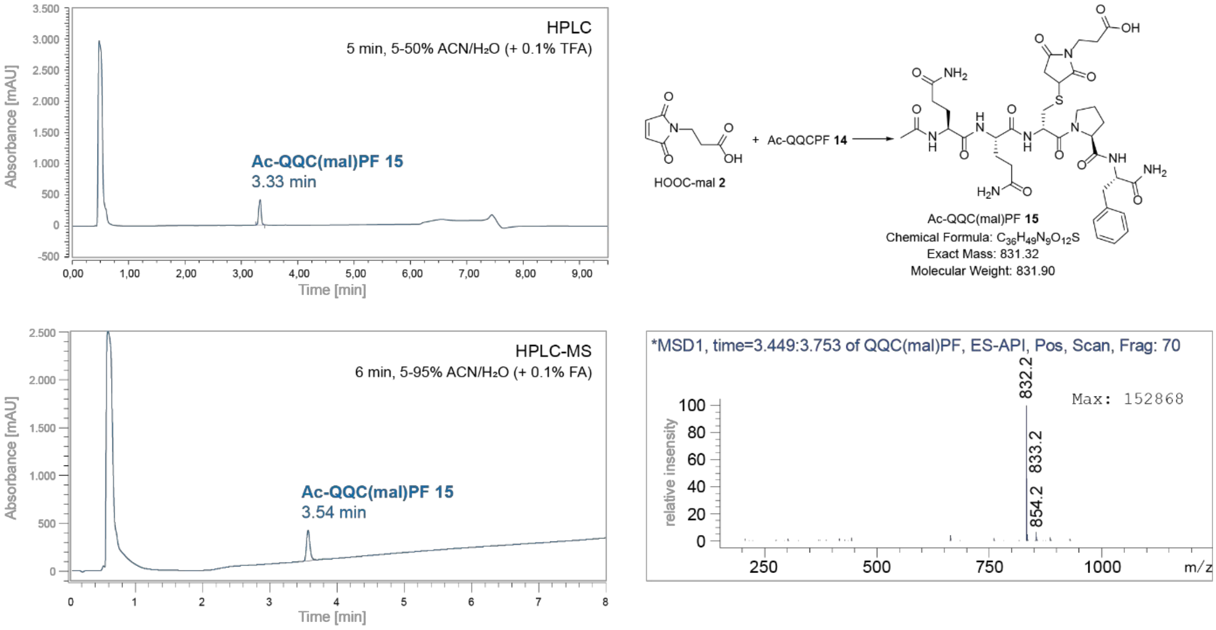

Figure S 27 HPLC and RP-HPLC-MS of QQC(mal)PF after 30 min of incubation in 50 mM NaOAc buffer (pH 5.5). The same sample was first injected into an HPLC and immediately afterwards into an RP-HPLC-MS. RP-HPLC-MS confirmed the product at 3.54 min retention time. (injection volume = 5 µL for both runs, both UV traces at 220nm)

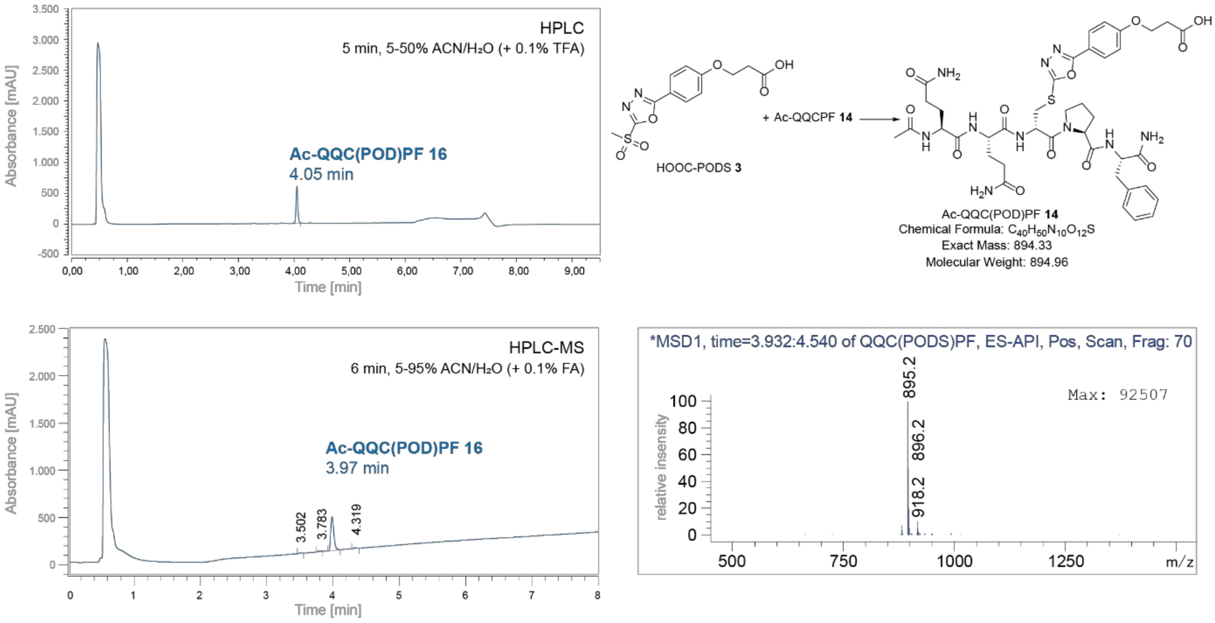

Figure S 28 HPLC and RP-HPLC-MS of QQC(PODS)PF after 60 min of incubation in 50 mM NaOAc buffer (pH 5.5). The same sample was first injected into an HPLC and immediately afterwards into an RP-HPLC-MS. RP-HPLC-MS confirmed the product at 4.07 min retention time. (injection volume = 5 µL for both runs, both UV traces at 220nm)

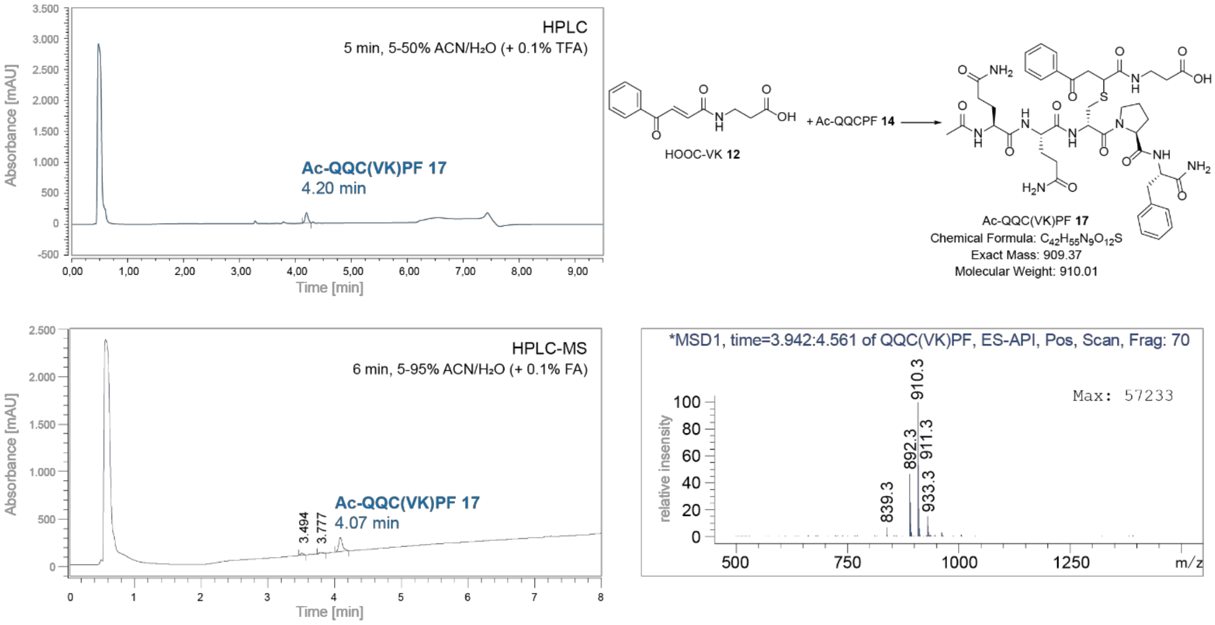

Figure S 29 HPLC and RP-HPLC-MS of QQC(mal)PF after 30 min of incubation in 50 mM NaOAc buffer (pH 5.5). The same sample was first injected into an HPLC and immediately afterwards into an RP-HPLC-MS. RP-HPLC-MS confirmed the product at 3.97 min retention time. (injection volume = 5 µL for both runs, both UV traces at 220nm)

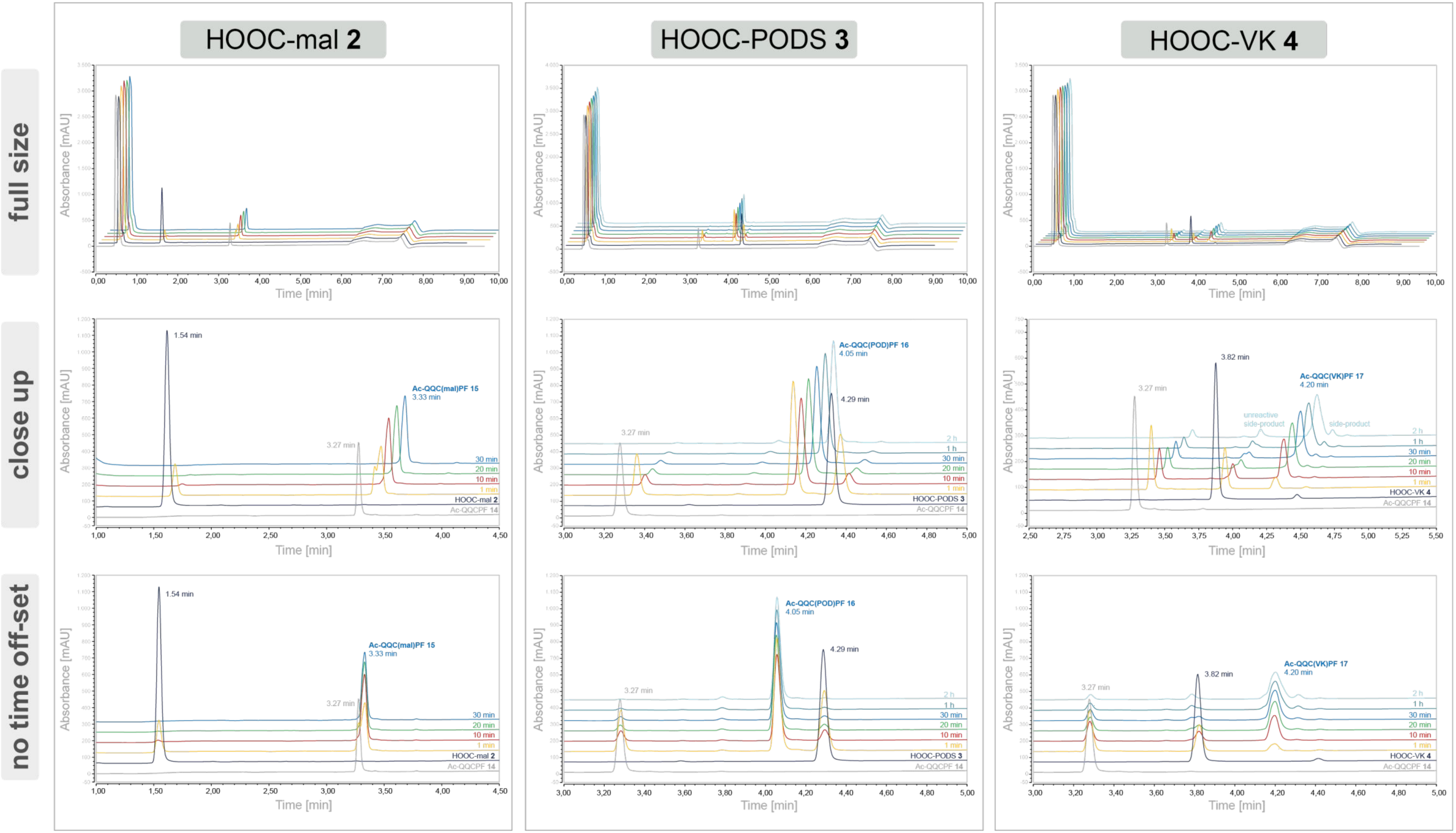

Figure S 30 Comparison of the reactions of mal-COOH **2**, PODS-COOH **3**, or VK-COOH **4** with the *N*-acetylated, Cys-containing amino acid sequence of HSA ($HSA^{32\text{-}36}$, Ac-QQCPF **14**) at equimolar incubation (0.25 mM) performed at 37°C. Stacked UV-traces (λ=220 nm) of the HPLC runs performed every 10 min. Each column of stacked chromatograms shows the same comparison from different angles.

VI. DTNB assay.

The DTNB assay was adapted for small sample volumes from a previously described procedure.[3] Briefly, 10 µL of protein sample was added to 100 µL 0.1 M NaH_2PO_4/1 mM EDTA buffer (pH 8) in an 1.5 mL low-bind reaction tube to yield a final protein concentration of 1 mM. 2 µL of freshly prepared DTNB stock solution (10 mM in H_2O) were added and the solution was incubated for 20 min. The absorbance was measured at $\lambda = 412$ nm using a microvolume UV/VIS spectrophotometer. The protein blank was measured using buffer and protein in the absence of reagent. The sample was measured 5 times, and the protein blank value was deducted from the mean value of the sample measurements. The thiol concentration was calculated from a cysteine standard curve (0.1 – 1.6 mM *N*-acetyl-*L*-cysteine). The standard curve was obtained via preparation of 5 defined concentrations equally to the sample preparation described for the protein sample. The results were expressed as [SH]/[HSA] = % free thiol. The modified method gave the same results as for the published procedure.

VII. Reduction of Cys34 of HSA.

TCEP was prepared as 50 mM stock solution in water. 1 equiv. of TCEP (0.30 mmol, 6 µL) was added to 20 mg of HSA (0.30 mmol, 282 µL, 1.07 mM in PBS) and incubated for 15 min at 20°C. HSA was purified in a first step via PD-10 column to 3 mL 0.9% NaCl, according to manufacturer's instructions. Subsequently, the product was washed three times via spin filtration (Amicon 4, 30 kDa MWCO, 4 000 g, 15 min, 20°C) using 0.9% NaCl.

VIII. Isotopic dilution assay.

In order to determine the chelator per protein ratio, an isotopic dilution assay was performed as described earlier, with adaptions for the radiolabeling with ^{89}Zr.[4] In brief, a zirconium stock solution was prepared ($Zr(IV)(acac)_4$, 752.5 µM in 1 M oxalic acid), of which 500 µL were spiked with ~10 MBq of $[^{89}Zr]Zr(oxalate)_2$ (BV Cyclotron VU Facility Amsterdam, Revvity). Then, 2 µL of the $[^{89}Zr]Zr$ stock solution were neutralized with 1 µL 2 M Na_2CO_3 and 10 µL of radiolabeling buffer were added. At last, 10 µL of a conjugate were added (~10 mg/mL, the exact concentration of each conjugate was determined via microvolume UV/VIS). The radiolabeling solutions were incubated for 1 h, stopped with 2 µL of 25 mg/mL EDTA, analyzed via TLC and the chelator per protein ratio was calculated as described earlier.

IX. Size exclusion chromatograms of DFO*-HSA derivatives

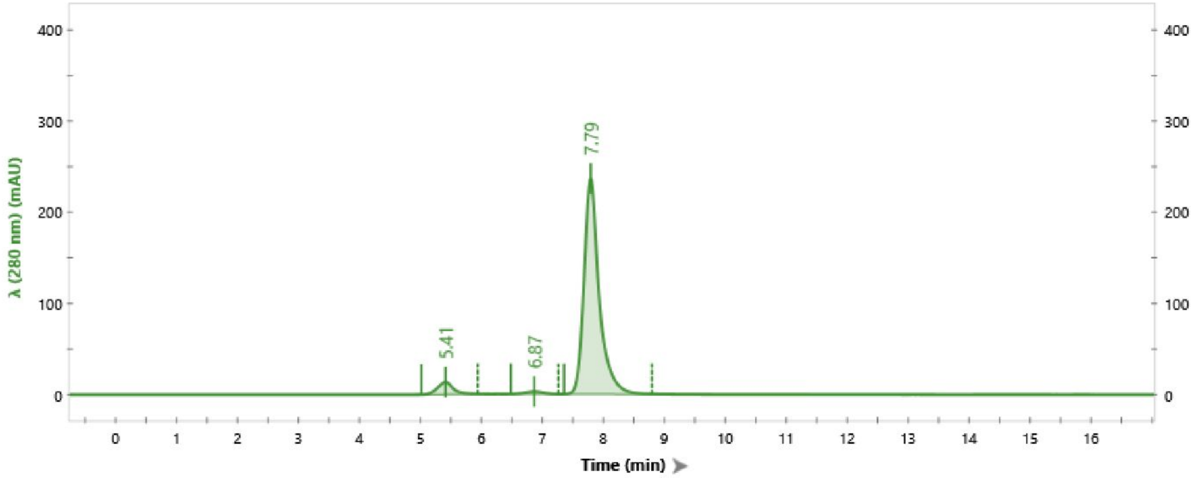

Figure S 31 SEC chromatogram (UV, λ=280 nm) of DFO*malHSA **8**.

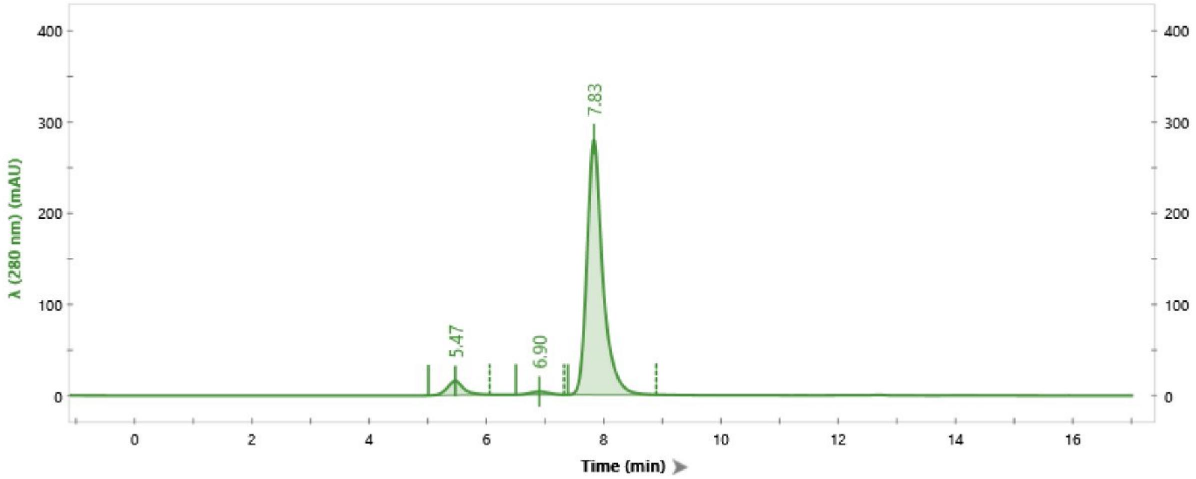

Figure S 32 SEC chromatogram (UV, λ=280 nm) of DFO*-POD-HSA **9**.

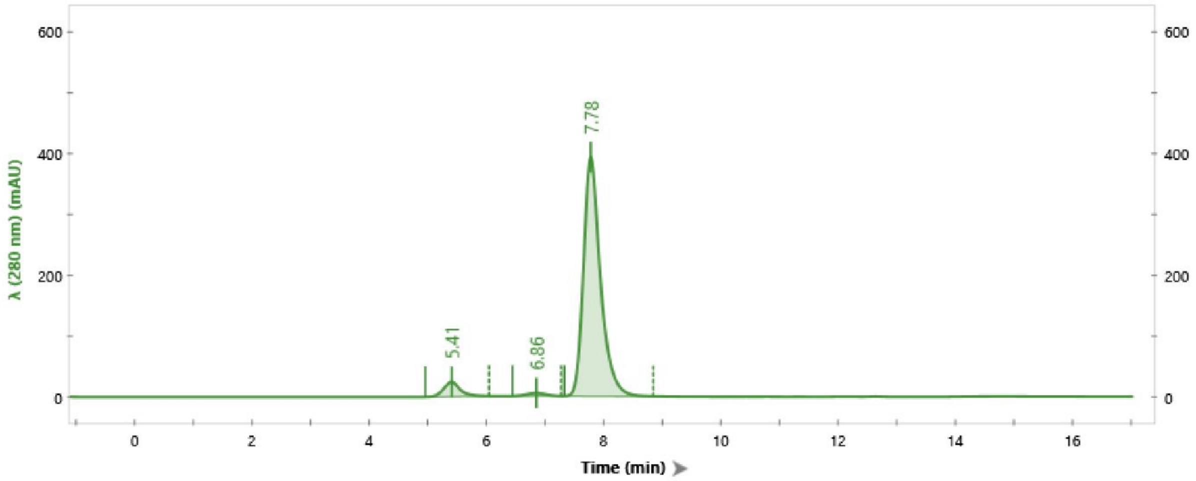

Figure S 33 SEC chromatogram (UV, λ=280 nm) of DFO*-VK **10**.

X. Additional data of isotopic dilution assay

pH	chelator [equiv.]	t [h]	available thiol per HSA**	chelator-to-protein ratio**		
				**8**	**9**	**10**
PBS (7.4)	1	3.5	0.24	-	0.01	0.17
PBS (7.4)	4	5.5	0.24	0.65*	0.12	0.46*
PBS (7.4)	4	5.5	0.53	0.65*	0.19	-

Table S 1 Additional data for bioconjugation reactions of **8**, **9** and **10** in NaOAc or PBS at pH 5.5 or 7.4, respectively, with obtained chelator-to-protein ratios relative to available thiols per HSA molecule. * Determined by DTNB assays. ** Determined by isotopic dilution assays. *** Value higher than available free thiols indicate unselective reaction of the BFCA with other amino acid residues.

XI. Schematic representation of the quality control methods

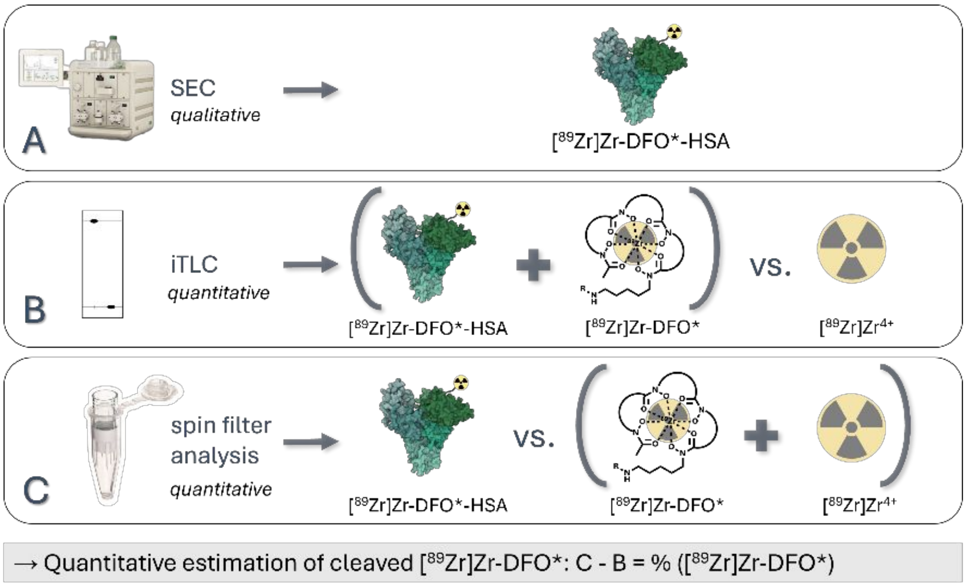

Figure S 34 Schematic representation of the quality control methods required for comprehensive characterization of HSA-based radiotracers. A) SEC for qualitative information about successful radiolabeling of the protein and detection of aggregates. B) iTLC for determination of the amount of free radionuclide ($[^{89}Zr]Zr^{4+}$) (r_f=1) vs. the fraction of radiolabeled chelator and protein ($[^{89}Zr]$Zr-DFO* and $[^{89}Zr]$Zr-DFO*-HSA, both r_f=0). C) Spin filtration analysis enabling the separation of low-molecular weight species (free $[^{89}Zr]Zr^{4+}$ and $[^{89}Zr]$Zr-DFO*) from the protein. The structure of HSA was rendered from PDB 1AO6[5] using Mol*[6].

XII. Size exclusion chromatograms of radiolabeled DFO*-HSA derivatives

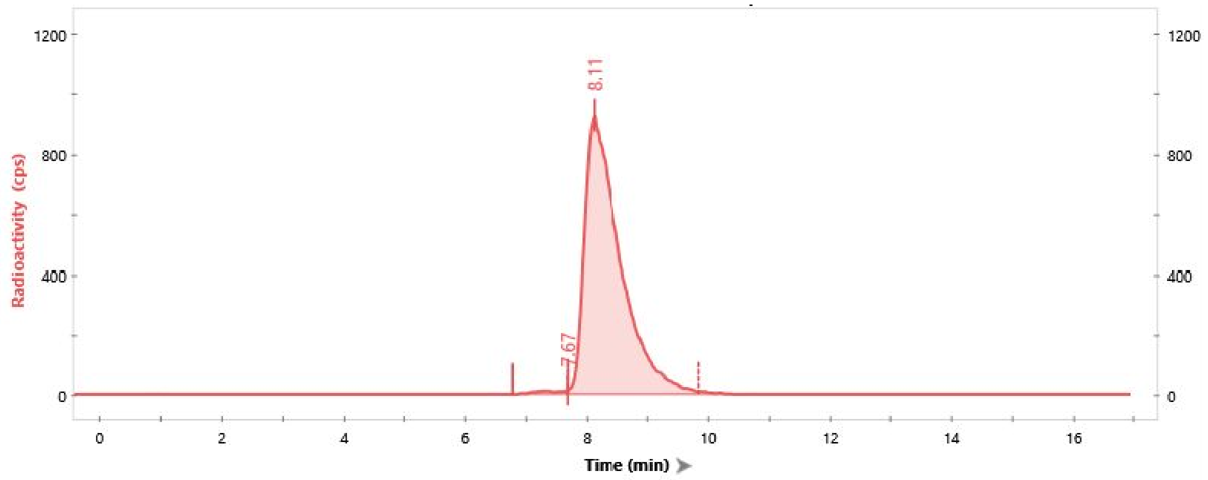

Figure S 35 SEC chromatogram (radio channel) of [^{89}Zr]Zr-DFO*malHSA.

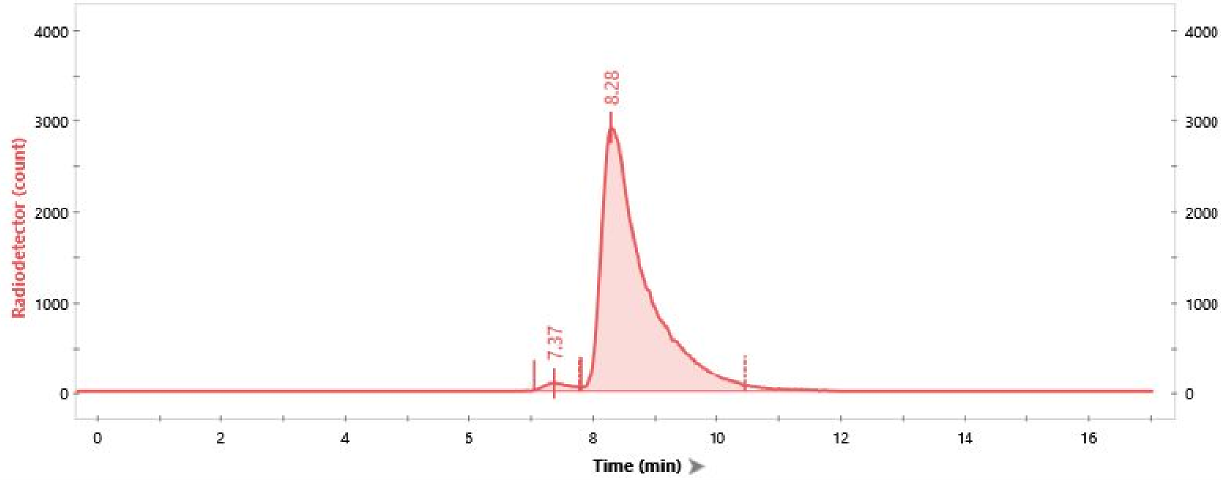

Figure S 36 SEC chromatogram (radio channel) of [^{89}Zr]Zr-DFO*-POD-HSA **18a**.

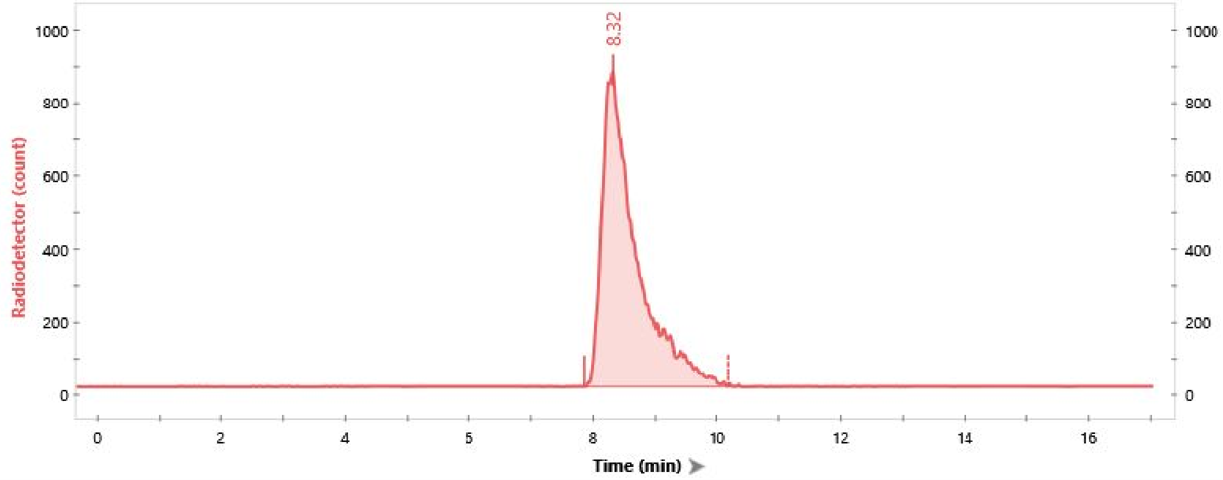

Figure S 37 SEC chromatogram (radio channel) of [^{89}Zr]Zr-DFO*-VK-HSA **19a**.

XIII. Cell culture.

The human colorectal cancer model SW480 and the murine (Balb/c) colorectal cancer model CT26 were used and grown, unless otherwise stated, in either Minimum Essential Medium (MEME) or Dulbecco's Modified Eagle Medium/Nutrient Mixture F-12 (DMEM/F-12, 1:1) medium, respectively. Both cell lines were purchased from American Type Culture Collection (ATCC, Rockville, MD, USA). The cells were grown in a humidified incubator (37 °C, 5% CO_2) in the above-mentioned medium containing 10% FCS (PAA, Linz, Austria). All cells were cultivated without the addition of antibiotics and were regularly checked for mycoplasma contamination.

SW480 cells were plated in 12-well plates ($2.5x10^5$ cells ml^{-1} $well^{-1}$) and left to recover for 24 h, followed by 24 h serum starvation by exchanging the FCS-containing medium with a serum-free RPMI medium. Before the addition of 100 µL radiotracer solution [^{89}Zr]Zr-DFO*-POD-HSA **12** (0.1, 1 and 10 nM in 0.9% NaCl), the cells were washed with Dulbecco′s Phosphate Buffered Saline (DPBS). Then, 900 µL RPMI-1640 medium were added to each well and the plates were incubated in triplicates for 3 h at 37 °C and 5% CO_2 atmosphere. The supernatant was collected in a tube together with the two times 1 mL DPBS used for washing the cells. The cells were then lysed with 1 mL 1 M NaOH solution at 37 °C. The solution was collected in a separate tube together with the two times 1 mL DPBS used for washing the wells, again. The separated fractions from cells and supernatant were measured in a γ-counter, the measured counts were decay and background corrected, and the percentage of internalized radioactivity was calculated as fraction of internalized versus total radioactivity.

XIV. Cellular uptake of radiolabeled DFO*-HSA derivatives

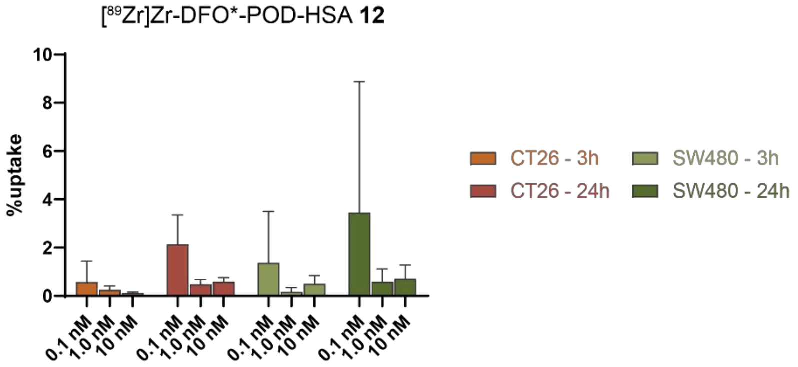

Figure S 38 In vitro uptake of of [^{89}Zr]Zr-DFO*-POD-HSA **12** in CT26 and SW480 cells at 3 different tracer concentrations (0.1, 1.0 and 10 nM). Uptake measured after 3 and 24 h of incubation at 37 °C and 5% CO_2. Experiments were performed in triplicates (n=3).

XV. Animal data

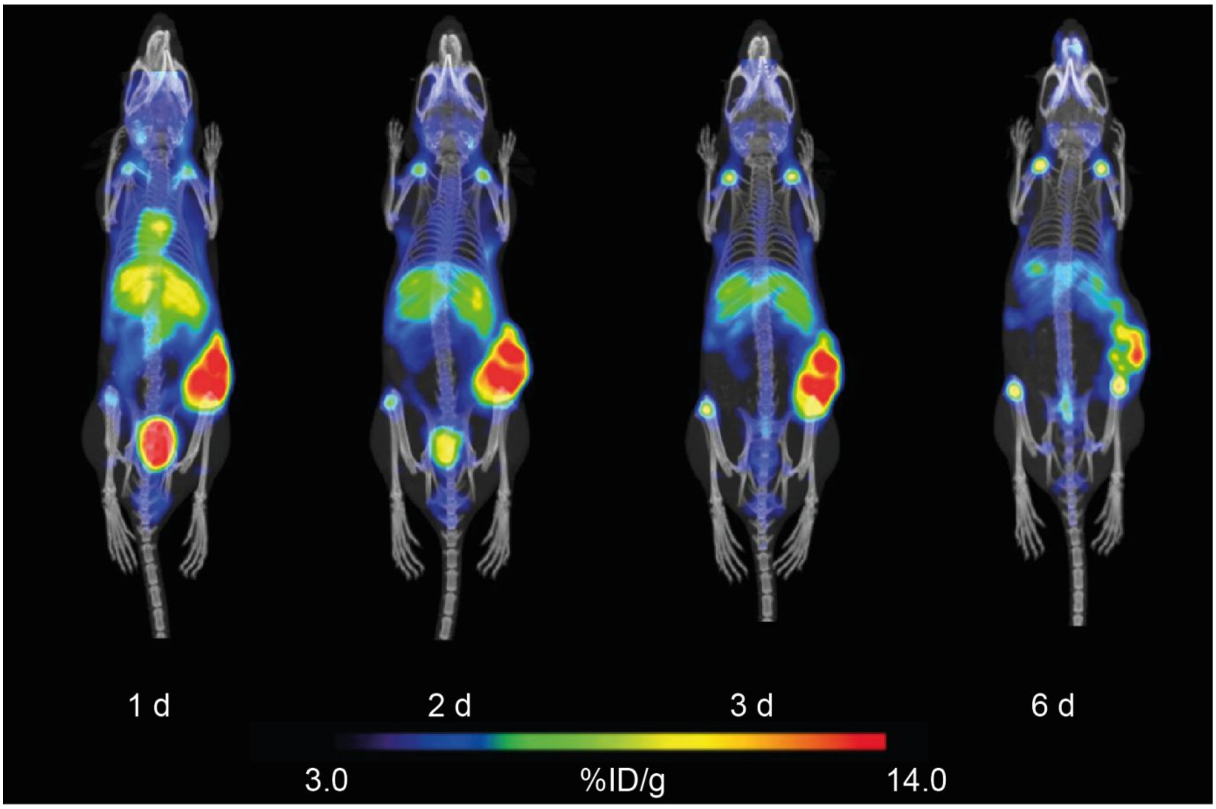

Figure S 39 Maximum intensity projection (MIP) PET images fused with CT of Balb/c mouse no. 1 bearing subcutaneous CT26 tumors are shown for 1, 2, 3 and 6 d p.i. 10.4 MBq [^{89}Zr]Zr-DFO*-POD-HSA **12.** PET data is decay corrected for the start of acquisition.

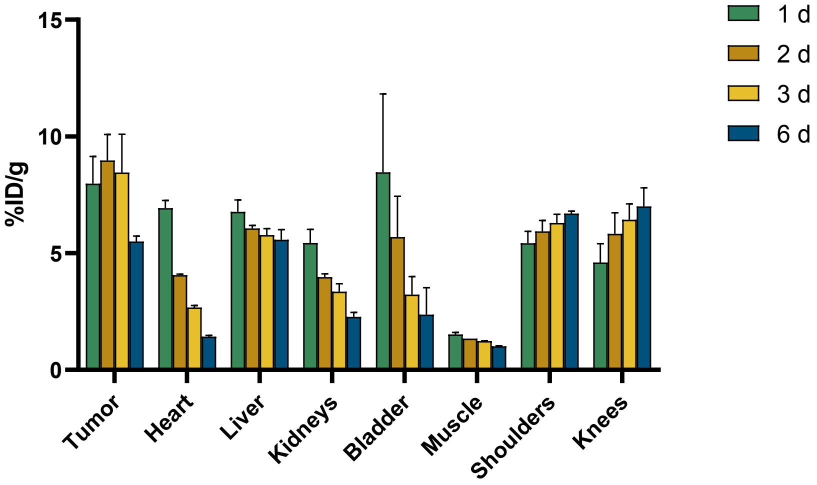

Figure S 40 PET image quantification of [^{89}Zr]Zr-DFO*-POD-HSA **12** for selected organs (ID%/g) at different time points (1, 2, 3 and 6 d (n=2 each)). Imaging data was corrected for the start of PET acquisition. %ID/g values are mean values of selected ROIs.

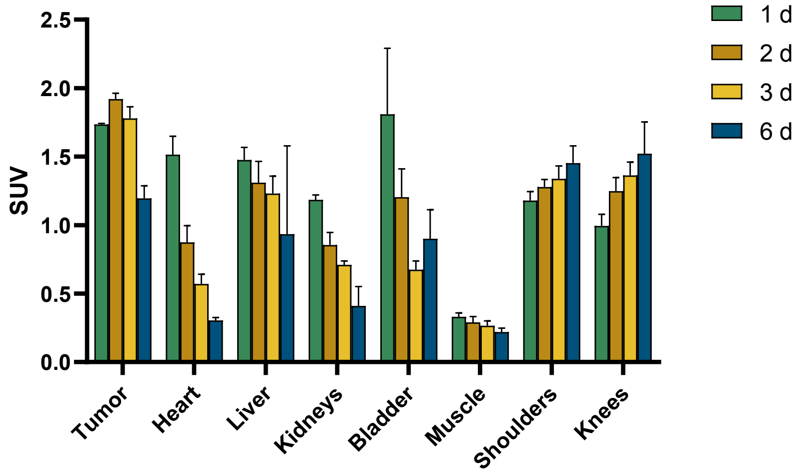

Figure S 41 Standardized uptake values (SUV) of [^{89}Zr]Zr-DFO*-POD-HSA **12** represent %ID/cc normalized to body weight at different time points (1, 2, 3 and 6 d (n=2 each)). Imaging data was corrected for the start of PET acquisition. SUV values are mean values of selected ROIs

	**24 h**					**48 h**					*24 vs 48 h*
	**11**		**12**		α	**11**		**12**		α	**12**
	%ID/g	n	%ID/g	n		%ID/g	n	%ID/g	n		α
Blood	8.2 ± 2.3	4	9.4 ± 1.3	4	ns	3.9 ± 0.3	5	4.4 ± 0.4	4	ns	**
Tumor	9.1 ± 1.8	4	17.1 ± 3.4	4	**	10.6 ± 3.0	4	13.8 ± 3.2	4	ns	ns
Heart	3.8 ± 0.3	4	5.3 ± 0.2	4	***	3.5 ± 0.4	5	4.3 ± 0.6	4	ns	ns
Lung	3.5 ± 0.3	3	5.9 ± 1.1	4	*	3.1 ± 0.3	5	3.5 ± 0.4	4	ns	ns
Liver	5.2 ± 0.5	4	8.7 ± 0.4	4	****	6.2 ± 0.5	5	8.7 ± 0.7	4	***	ns
Spleen	4.0 ± 0.3	4	6.1 ± 1.0	4	*	5.4 ± 2.3	5	6.6 ± 1.5	4	ns	ns
Kidney	5.8 ± 0.6	4	8.1 ± 0.4	4	***	5.3 ± 0.9	5	7.2 ± 0.4	4	**	ns
Stomach	1.3 ± 0.1	4	1.9 ± 0.2	4	**	1.1 ± 0.2	5	1.7 ± 0.3	4	*	ns
Pancreas	1.5 ± 0.2	4	2.3 ± 0.4	4	*	1.4 ± 0.2	5	2.0 ± 0.3	4	**	ns
Intestine	2.0 ± 0.5	4	2.1 ± 0.5	4	ns	1.6 ± 0.4	5	1.7 ± 0.1	4	ns	ns
Colon	1.4 ± 0.2	4	2.7 ± 0.3	4	***	1.3 ± 0.4	5	2.2 ± 0.3	4	*	ns
Brain	0.2 ± 0.0	4	0.4 ± 0.1	4	**	0.1 ± 0.0	5	0.2 ± 0.0	4	****	*
Eyes	0.8 ± 0.1	4	1.7 ± 0.3	4	**	0.7 ± 0.1	5	1.6 ± 0.4	4	**	ns
Muscle	0.8 ± 0.2	4	1.4 ± 0.2	4	*	0.6 ± 0.2	5	1.0 ± 0.0	4	**	ns
Femur	1.4 ± 0.2	4	2.6 ± 0.3	4	***	1.4 ± 0.3	5	3.1 ± 0.4	4	***	ns

Table S 2. Biodistribution data of [^{89}Zr]Zr-DFO*malHSA **11** and [^{89}Zr]Zr-DFO*-POD-HSA **12** in Balb/c mice bearing a sc CT26 tumor obtained at 24 and 48 h via gamma-counter measurement and calculation of %ID/g tissue. Decay correction was performed for the time of injection. Data for [^{89}Zr]Zr-DFO*malHSA **11** was taken from our previously published manuscript.[3] Statistical analysis was performed via t-test (comparison of different radiotracers) or paired t-test (comparison of different time points). ($p >= 0.05$ = ns, $p < 0.05$ = *, $p < 0.01$ = **, $p < 0.001$ = ***, $p < 0.0001$ = ****)

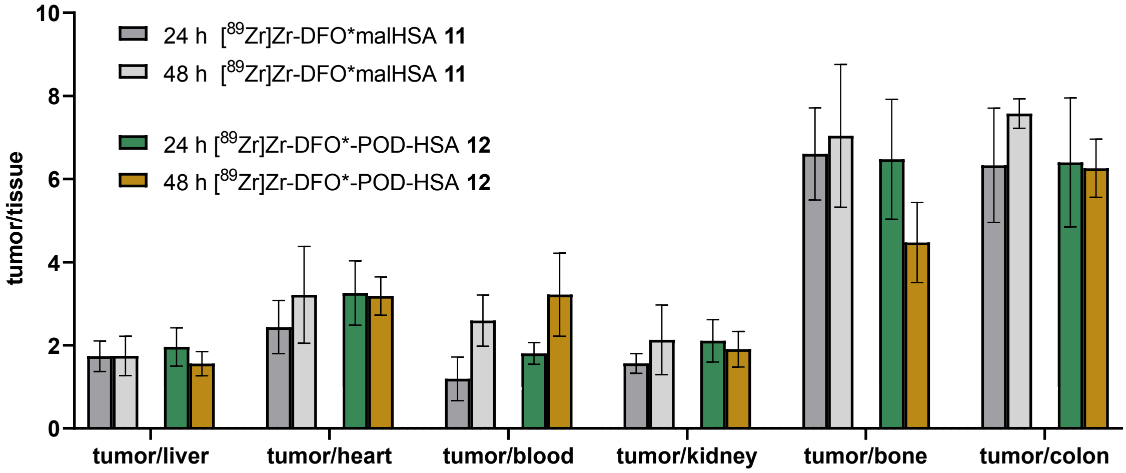

Figure S 42 Direct comparison of tumor/tissue ratios of [^{89}Zr]Zr-DFO*malHSA **11** and [^{89}Zr]Zr-DFO*-POD-HSA **12**.

Literature References

(1) Pace, C. N.; Vajdos, F.; Fee, L.; Grimsley, G.; Gray, T. How to Measure and Predict the Molar Absorption Coefficient of a Protein. *Protein Sci. Publ. Protein Soc.* **1995**, *4* (11), 2411–2423. https://doi.org/10.1002/pro.5560041120.

(2) Guarrochena, X.; Kronberger, J.; Tieber, M.; Ciesielski, P.; Mindt, T. L.; Feiner, I. V. J. Straightforward Synthesis of DFO* - An Octadentate Chelator for Zirconium-89. *ChemMedChem* **2024**, *19* (3), e202300495. https://doi.org/10.1002/cmdc.202300495.

(3) Kronberger, J.; Balber, T.; Schueffl, H.; Wahrmann, R.; Federa, A.; Gradl, M.; Brandt, M. R.; Wanek, T.; Mitterhauser, M.; Kowol, C. R.; Mindt, T. L.; Heffeter, P. Site-Selectively Functionalized Albumin with DFO*Maleimide for 89Zr-Radiolabeling Yields a Metabolically Stable PET Probe That Enables Late Time-Point Tumor Imaging in Mice. *J. Med. Chem.* **2025**. https://doi.org/10.1021/acs.jmedchem.5c00803.

(4) Meares, C. F.; McCall, M. J.; Reardan, D. T.; Goodwin, D. A.; Diamanti, C. I.; McTigue, M. Conjugation of Antibodies with Bifunctional Chelating Agents: Isothiocyanate and Bromoacetamide Reagents, Methods of Analysis, and Subsequent Addition of Metal Ions. *Anal. Biochem.* **1984**, *142* (1), 68–78. https://doi.org/10.1016/0003-2697(84)90517-7.

(5) Sugio, S.; Kashima, A.; Mochizuki, S.; Noda, M.; Kobayashi, K. Crystal Structure of Human Serum Albumin at 2.5 Å Resolution. *Protein Eng. Des. Sel.* **1999**, *12* (6), 439–446. https://doi.org/10.1093/protein/12.6.439.

(6) Sehnal, D.; Bittrich, S.; Deshpande, M.; Svobodová, R.; Berka, K.; Bazgier, V.; Velankar, S.; Burley, S. K.; Koča, J.; Rose, A. S. Mol* Viewer: Modern Web App for 3D Visualization and Analysis of Large Biomolecular Structures. *Nucleic Acids Res.* **2021**, *49* (W1), W431–W437. https://doi.org/10.1093/nar/gkab314.
